# Amide-to-Ester Substitution as a Strategy for Optimizing PROTAC Permeability and Cellular Activity

**DOI:** 10.1021/acs.jmedchem.1c01496

**Published:** 2021-12-09

**Authors:** Victoria G. Klein, Adam G. Bond, Conner Craigon, R. Scott Lokey, Alessio Ciulli

**Affiliations:** † Department of Chemistry and Biochemistry, University of California Santa Cruz, Santa Cruz, California 95064, United States; ‡ Division of Biological Chemistry and Drug Discovery, School of Life Sciences, University of Dundee, Dow Street, Dundee DD1 5EH, Scotland, U.K.

## Abstract

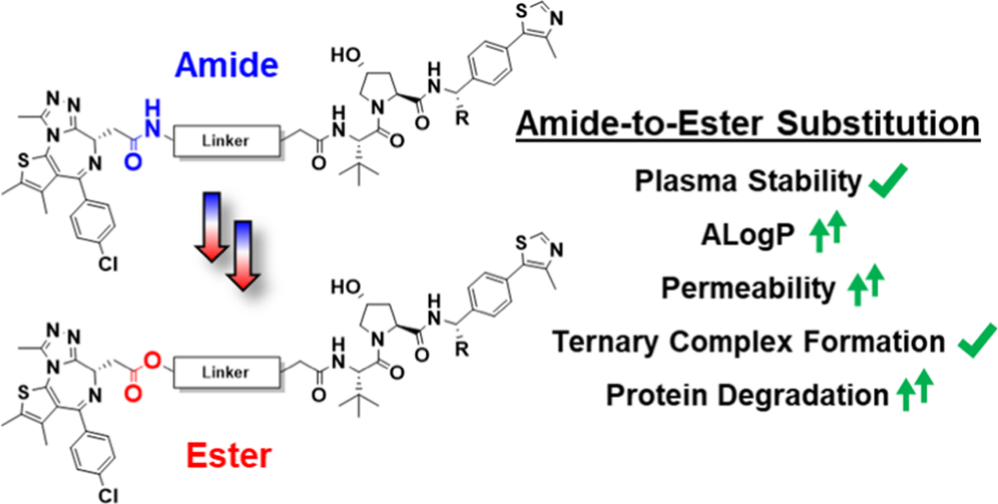

Criteria for predicting the druglike properties of “beyond Rule of 5” Proteolysis Targeting Chimeras (PROTAC) degraders are underdeveloped. PROTAC components are often combined via amide couplings due to their reliability. Amides, however, can give rise to poor absorption, distribution, metabolism, and excretion (ADME) properties. We hypothesized that a bioisosteric amide-to-ester substitution could lead to improvements in both physicochemical properties and bioactivity. Using model compounds, bearing either amides or esters, we identify parameters for optimal lipophilicity and permeability. We applied these learnings to design a set of novel amide-to-ester-substituted, VHL-based BET degraders with the goal to increase permeability. Our ester PROTACs retained intracellular stability, were overall more potent degraders than their amide counterparts, and showed an earlier onset of the hook effect. These enhancements were driven by greater cell permeability rather than improvements in ternary complex formation. This largely unexplored amide-to-ester substitution provides a simple strategy to enhance PROTAC permeability and bioactivity and may prove beneficial to other beyond Ro5 molecules.

## Introduction

Targeted protein degraders, also known as Proteolysis Targeting Chimeras (PROTACs), are becoming a widespread source of chemical probes and lead compounds that degrade rather than inhibit target proteins, providing a different drug modality with the potential to expand the “druggable” proteome.^1−7^ These chimeric molecules typically contain a protein-of-interest (POI)-targeting ligand (or warhead) and a ligand that binds to an E3 ligase connected by a linker.^8−10^ PROTAC-induced ternary complexes between the POI and E3 ligase are required for polyubiquitination and subsequent targeted degradation of the POI.^11^ PROTACs do not require full target occupancy because a single PROTAC molecule can induce degradation of more than one target protein molecule over time, thereby acting catalytically at substoichiometric target occupancy. These distinct features of PROTACs mode of action have been shown to result in increased target selectivity, higher potencies, and fewer off-target effects compared to small molecule inhibitors.^10,12−14^ Furthermore, unlike small molecule inhibitors, PROTACs can bind the target at any position, including nonfunctional binding sites.^10,15^ Notably, PROTACs have shown to be developable for use in humans, with several compounds reaching the clinic, including ARV-110 and ARV-471 that have recently progressed into phase II clinical trials for prostate and breast cancers, respectively, demonstrating both safety and efficacy in patients.^16−18^

While PROTACs harbor several advantages as a new modality within drug discovery, their bifunctional nature and chemical composition mean that they are inherently larger than the warhead ligands on which they are based. This makes PROTAC compounds go beyond the “Rule of 5” (bRo5) and can impose hurdles to their pharmaceutical development.^19−22^ Thus, efforts have been made recently to better understand the physicochemical properties and structure–property relationships of PROTACs to identify design parameters that may help guide development in this chemical space.^22−27^ An important pharmacokinetic hurdle for high-molecular-weight compounds tends to be permeability.^28,29^ Uptake into cells occurs in competition with efflux, which is also commonly a problem for large molecules.^30^ Indeed, recently, we and others have established that PROTACs can show potent cellular activity despite exhibiting very low permeabilities compared to their individual ligand components and to more conventional inhibitors.^31−33^ There is therefore a great interest to develop strategies for improving cell permeability and other physicochemical properties of PROTACs.

We wondered whether PROTAC degradation activity could be improved by increasing their cellular permeability. To this end, it is worth keeping in mind that requirements on cellular permeability are relaxed because, unlike inhibitors, PROTACs do not have to fully occupy the target binding site for the duration of their action. Indeed, the catalytic mode of action of PROTAC degraders via the formation of stable ternary complexes can compensate for lower membrane permeability, as we have shown for the archetypical BET degrader MZ1.^31,34^ However, optimal ternary complexes are often challenging to achieve without a “trial-and-error” approach involving the synthesis and testing of many compounds.^35,36^ Thus, we aimed to develop a set of simple parameters for PROTAC optimization, which could be applied during initial compound design or to existing PROTACs to improve bioactivity through increased membrane permeability.

In our previous work, we demonstrated that an amide-to-ester substitution at the *tert*-Leu of the von Hippel–Lindau (VHL)-recruiting ligand can increase membrane permeability.^31^ While effective, this ester modification yielded only a modest increase in permeability over their amide counterparts due to the relatively high steric shielding at this position from the β-branched amino acid side chain.^37^ We hypothesized that, alternatively, substituting the amide connecting the linker to the POI warhead for an ester would lead to a larger increase in permeability. Therefore, to build on our proof-of-concept study, we developed a systematic set of compounds to test this hypothesis across a wide range of lipophilicities (ALog *P*) and linker lengths. By applying the insights from these model compounds, we show that the correct combination of an amide-to-ester substitution and ALog *P* modulation dramatically increased the membrane permeability of known bromodomain and extra terminal (BET) protein targeting PROTACs, MZ1, and ARV-771.^3,38^ These subtle structural modifications have also led to an increased ability to degrade BET proteins and induce cytotoxicity, while maintaining both stable ternary complex formation and plasma stability.

## Results and Discussion

### Model Compound “Liposcan” Reveals Ideal Lipophilicity Range for Increased Permeability

**Figure 1:**
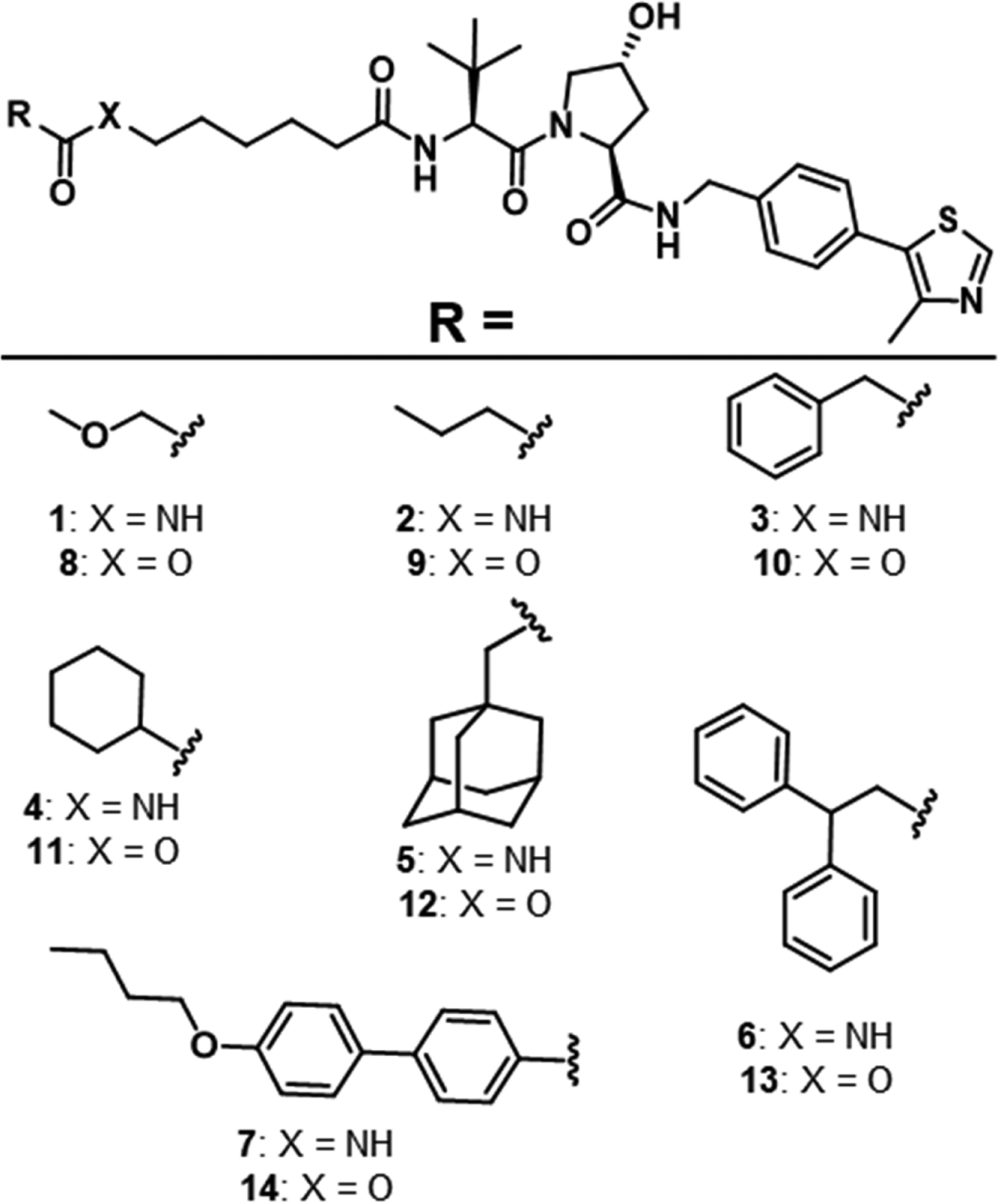
Liposcan model compound structures. Chemical structures of compounds organized by amide (**1–7**) and ester (**8–14**) matched pairs with warheads of varying lipophilicities.

It is important to consider lipophilicity during compound design to attain molecules with favorable absorption, distribution, metabolism, excretion, and toxicity (ADMET) properties.^39^ While suggested optimal lipophilicity ranges exist for typical small molecule drugs^40^ and bRo5 compounds,^41^ design parameters for ideal PROTAC lipophilicity remain unclear. We set out to perform a systematic investigation into the effect of lipophilicity on permeability for a set of seven VHL-based “PROTAC-like” model compounds (**1–7**; Figure 1). All compounds contained the VHL ligand VH032 as their E3 ligase-targeting ligand.^42^ We modulated the compounds’ lipophilicities using a variety of simple warheads as surrogates of POI ligands across a range of calculated lipophilicities (ALog *P*) from 1.2 to 6.0. As permeability can be strongly affected by molecular weight (MW) and the number of hydrogen bond donors (HBDs) and acceptors (HBAs), we kept these values in a relatively narrow range (MW = 600–800, HBD = 3–4, HBA = 6–8; SI Table 1). Furthermore, we used a short alkyl linker for compounds **1**–**7** to eliminate permeability-affecting intramolecular hydrogen bonds (IMHBs) that can be formed between poly(ethylene glycol) (PEG)-based linkers and amide −NHs in other parts of the molecule.^25,31^

**Figure 2:**
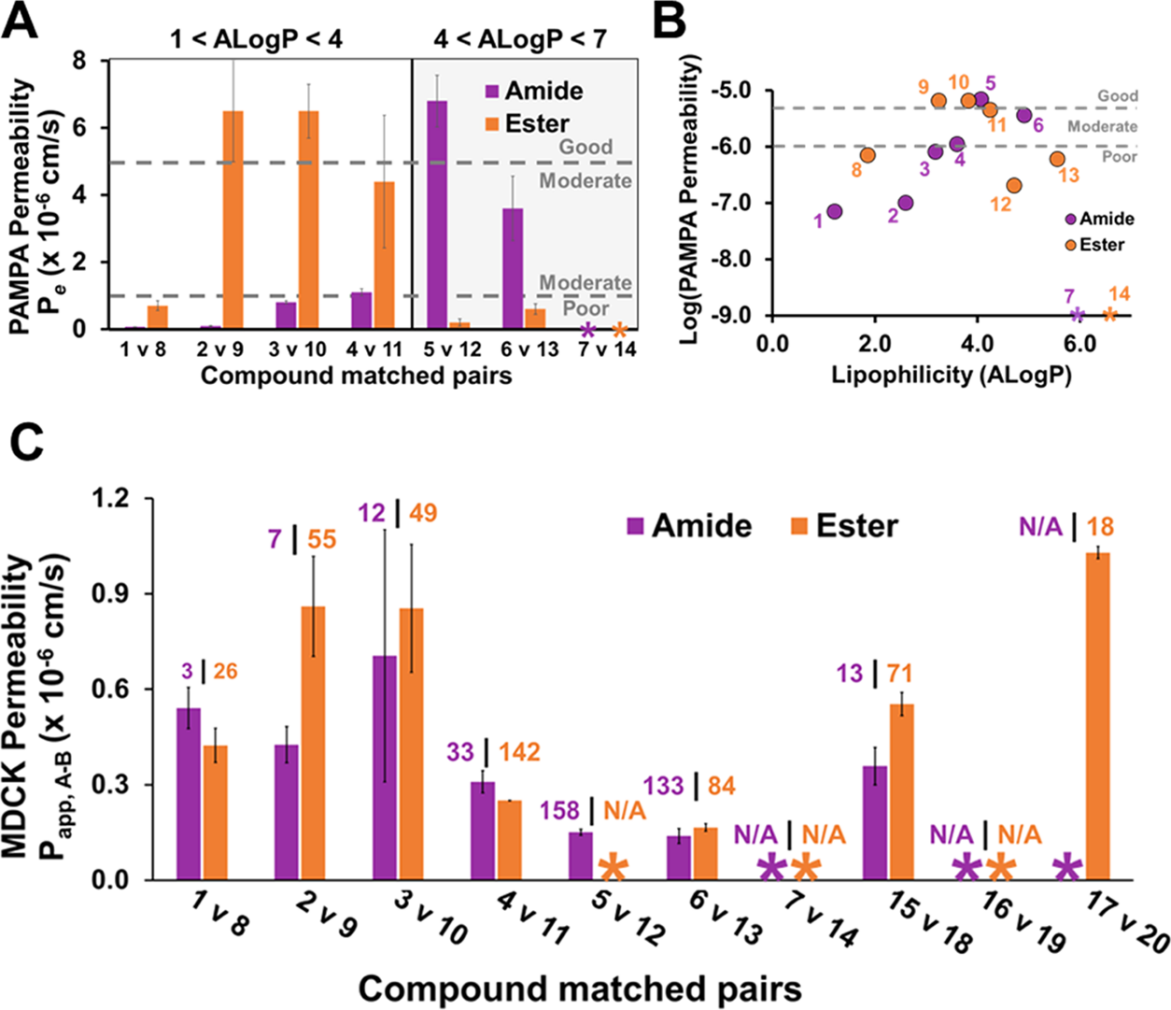
Liposcan model compound permeabilities. PAMPA permeabilities of model compounds organized by (A) amide (purple) and ester (orange) matched pair (error bars represent ± SD, *N* = 4) and (B) calculated lipophilicity (ALog *P*). Dashed gray lines represent categorical threshold for poor (*P*_e_ < 1 × 10^–6^ cm/s), moderate (1 × 10^–6^ cm/s < *P*_e_ < 5 × 10^–6^ cm/s), and good (*P*_e_ > 5 × 10^–6^ cm/s) membrane permeability. (C) MDR1-MDCK cell permeability of liposcan and linker scan model compounds by matched pair. The numbers above bars indicate the efflux ratio. *below limit of detection, N/A: efflux ratio could not be calculated. Error bars represent data range, *N* = 2.

We next investigated the effects of lipophilicity on membrane permeability using the parallel artificial membrane permeability assay (PAMPA), a high-throughput permeability assay that is generally well correlated to cell-based permeability measurements.^43^ Our group has shown that PAMPA is beneficial for studying compounds with low expected permeabilities due to the assay’s low limit of detection.^31^ Similar to other types of previously studied compounds,^41^ the permeabilities of the model compounds increased with ALog *P* up to an ALog *P* of around 4 (cf. **1–5**; Figure 2A,B and SI Table 1). Above an ALog *P* of 4, permeability decreased as ALog *P* increased (cf. **6–7**), with no detectable permeability for **7**, which had an ALog *P* of 6.0 (Figure 2A and Table 1). At these higher ALog *P* values (>4–5), compounds begin to lose aqueous solubility and become membrane retained, both of which can reduce passive membrane permeability.^44^ The data with this compound series suggest that PROTACs based on VH032 should be designed with an ALog *P* between 3 and 5 to bias them toward higher permeability, similar to other bRo5 compounds. Moreover, the relationship between lipophilicity and permeability offers a route to improve the permeability of PROTACs by making small structural modifications as needed to maintain ALog *P* within the optimal range.

**Table 1:**
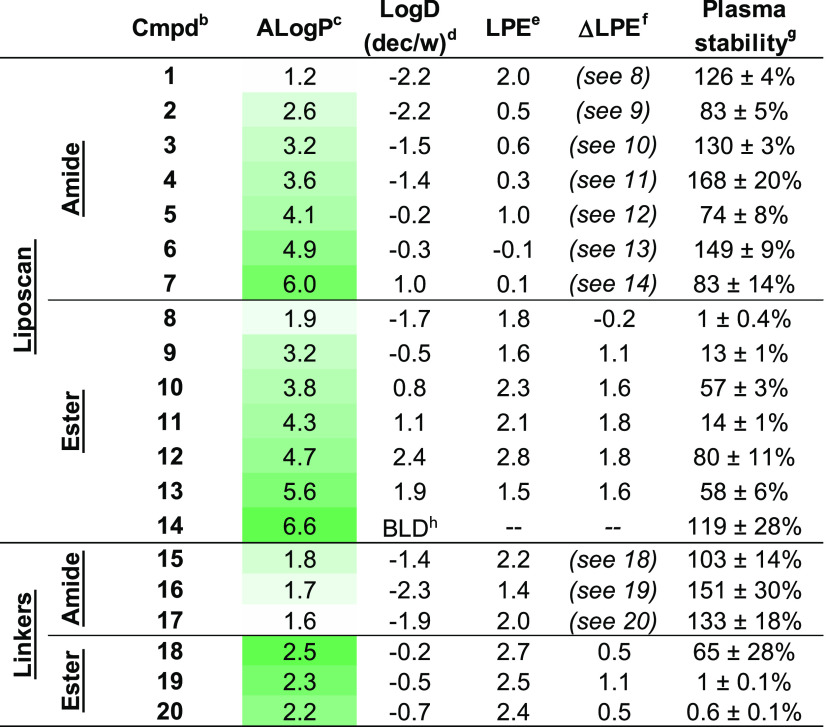
Physicochemical and ADME Properties of Model Compounds^a^

a Physicochemical properties including calculated lipophilicity (ALog *P*), experimental Log *D*_(dec/w)_, calculated LPE, and experimental plasma stability data of liposcan and linker scan model compounds for both amide and ester derivatives.

b Compound.

c Calculated lipophilicity.

d 1,9-Decadiene and PBS pH 7.4 shake flask partition coefficient.

e LPE = Log *D*_(dec/w)_ – 1.06(ALog *P*) + 5.47.

f ΔLPE = LPE_ester_ – LPE_amide_ by amide–ester matched pairs.

g % Compound remaining after 90 min in human plasma at 37 °C.

h Below limit of quantitation.

Recently, it has been shown that PROTACs can have a high efflux ratio in cell-based permeability assays.^32,33^ Therefore, we were interested in monitoring both the cell permeabilities and efflux ratios over this broad ALog *P* range. In bidirectional MDCK-MDR1 cells expressing human Pgp, amides **1**–**6** demonstrated generally low cell permeability, though these results were not strongly correlated to PAMPA or lipophilicity. As in PAMPA, **7** was below the limit of detection (Figure 2C and SI Table 2) in our MDCK assay. Additionally, amides **1**–**6** also had high efflux ratios, suggesting that they undergo active efflux.^45^ Interestingly, these efflux ratios were highly correlated to both lipophilicity and PAMPA permeability. Efflux ratios increased with lipophilicity up to an ALog *P* of around 4, peaking with **5**. As with PAMPA permeability, the efflux ratio decreased with increasing lipophilicity at ALog *P* values above 4 (SI Tables 1 and 2).

### Amide-to-Ester Substitutions Improve Membrane Permeability over a Broad ALog *P* Range

In addition to lipophilicity, the number of HBDs in compounds is a crucial determinant of permeability.^46,47^ Reducing the presence of solvent-exposed HBDs through *N*-methylation or occlusion from solvent by β-branching or other steric shielding are some of the strategies used to increase a compound’s membrane permeability.^37,48−50^ In a previous study, we demonstrated that substituting the *tert*-Leu amide of the VH032 ligand with an ester improved compound permeability by about 2-fold.^31^ We hypothesize that the relatively modest increase in permeability resulting from this amide-to-ester substitution was likely due to the partial shielding of the −NH from solvent by the adjacent β-branched α-carbon, limiting the permeability and reducing effects of this HBD.^31,37^ Additionally, substituting this amide (between the VH032 ligand and the linker) for an ester reduced its binding affinity toward the VHL protein.^31^ Therefore, we created a new set of compounds with an amide-to-ester substitution at the other end of the linker (adjacent to where a POI-ligand would be attached) in an effort to achieve a more significant increase in permeability while maintaining binding to the VHL E3 ligase.

This second set of ester-containing, liposcan compounds (**8**–**14**) had a similarly broad ALog *P* range of 1.9–6.6 and narrow ranges for MW, HBAs, and HBDs (Figure 1 and Table 1). These compounds were structurally identical to the previously described amides **1**–**7** except for an amide-to-ester substitution between the linker and the POI-ligand mimic, creating seven amide-to-ester matched pairs for permeability analysis. Over an ALog *P* range of 1–4, the esters, **8**–**14**, were 4- to 65-fold more permeable than their amide counterparts (Figure 2A and SI Table 1). Substituting an amide for an ester not only removes a HBD but also increases the ALog *P* on average by about 0.6. Both the reduction of HBDs and increased lipophilicity are likely responsible for the increased permeability within this ALog *P* range.^37,41,51^ However, as expected, esters with an ALog *P* > 4 were less permeable than their respective amide counterparts (Figure 2 and SI Table 1). This is likely due to the established inverse relationship between permeability and lipophilicity as the ALog *P* increases over 4 due to a decreased aqueous solubility and increased membrane retention of the compound.^52^ Furthermore, it is possible that the additional HBD present in the amide series conferred increased solubility over the ester derivatives. Similar to amide **7** (ALog *P* = 6), its ester counterpart, **14** (ALog *P* = 6.6), had no detectable permeability (Figure 2A and SI Table 1).

Notably, the esters achieved their peak permeability at a lower lipophilicity than the amides, at ALog *P* = 3.2 vs ALog *P* = 4.1, respectively (Figure 2B). This ability to achieve higher membrane permeability at lower lipophilicities has important implications for drug development, as increased lipophilicity has been linked to increased toxicity and decreased specificity in addition to other liabilities associated with diminished solubility.^53^ Though not as apparent as the PAMPA results, ester compounds had MDCK permeabilities that were also greater than or equal to their amide counterparts for the most part (Figure 2C). These MDCK cell and PAMPA permeabilities followed similar trends within the ester compound series, with **9** and **10** having the peak permeabilities in both assays (*P*_e_ = 6.5 × 10^–6^ cm/s; Figure 2 and SI Table 1). These two ester compounds (**9** and **10**) also had very high efflux ratios in the MDCK assay compared to their amide counterparts (**2** and **3**, respectively). High efflux likely contributes to the diminished improvement in the MDCK cell permeabilities of the esters relative to the amides, compared to those improvements observed in PAMPA. Much like the amide compounds, the ester series had a high efflux ratio that was similarly correlated to lipophilicity (SI Table 2). Overall, amide-to-ester substitution offers a highly effective strategy to improve PROTAC permeability over a wide range of lipophilicities.

### Amide-to-Ester Substitutions Increase Permeability for Several Linker Types

**Figure 3:**
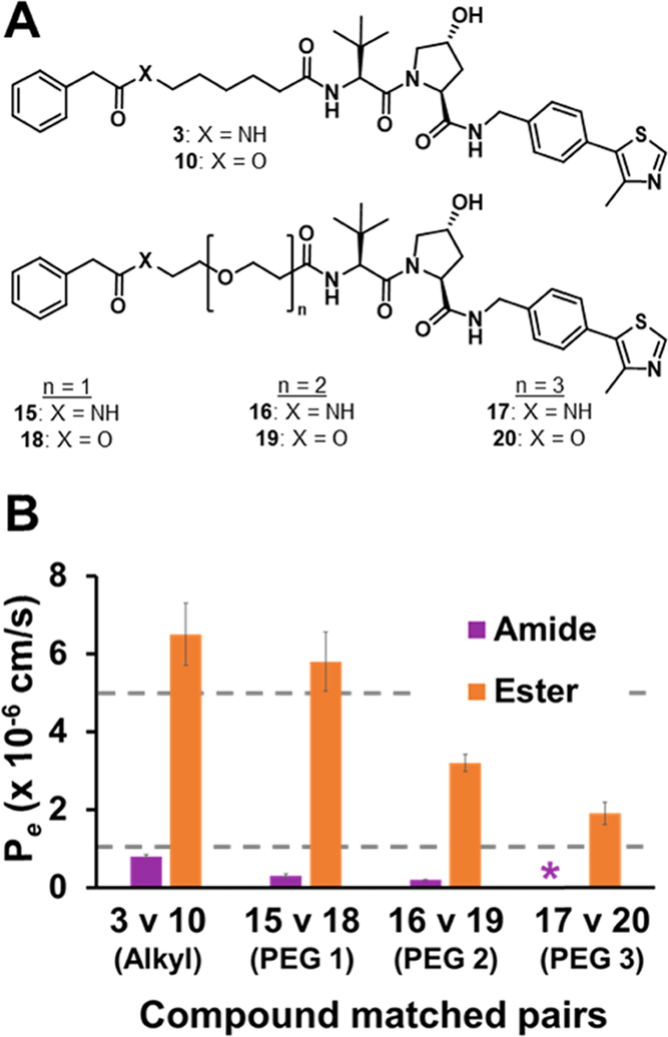
Linker scan model compound structures and permeabilities. (A) Chemical structures of linker scan model compounds and (B) PAMPA permeabilities of model compounds organized by amide (purple) and ester (orange) matched pair. Dashed gray lines represent categorical threshold for poor (*P_e_* < 1 × 10^–6^ cm/s), moderate (1 × 10^–6^ cm/s < *P*_e_ < 5 × 10^–6^ cm/s), and good (*P*_e_ > 5 × 10^–6^ cm/s) membrane permeabilities.

It has been suggested that short alkyl linkers may be better for PROTAC permeability, as they help minimize the already high topological polar surface area (TPSA) and the number of HBAs present.^32,33^ However, this hypothesis has not been fully tested. We have previously shown that the effect of the linker on PROTAC permeability can be confounded by hydrogen bonding and overall lipophilicity.^31^ For this study, we designed a systematic set of four compounds to assess the effects of linker length and composition on permeability by reducing the POI-ligand mimic to a simple benzyl group attached by an amide. The linkers varied from a short alkyl linker (**3**) to PEG-based linkers ranging from 1- to 3-PEG units in length (**15**–**17**, respectively) (Figure 3A and Table 1). The alkyl-linked compound **3** had the highest permeability. Permeability decreased with increasing PEG chain linker length, with **17** (3-PEG unit linker) showing no detectable permeability (Figure 3B and SI Table 1). This decrease in permeability is likely caused by a decrease in ALog *P* due to the increasing PEG chain length, consistent with the linear relationship between ALog *P* and permeability in this lipophilicity range (Table 1).

As an amide-to-ester substitution was found to improve the permeability of our first compound series of model compounds (**1**–**14**), we decided to make a second set of amide-to-ester compound matched pairs and synthesized esters **10** and **18**–**20** (Figure 3A). The esters all had detectable permeabilities that were 8- to 19-fold more permeable than their amide counterparts (Figure 3B and SI Table 1). Unlike the amides, which all had poor permeabilities (*P*_e_ < 1 × 10^–6^ cm/s), all of the esters had modest to good permeabilities (1 × 10^–6^ cm/s < *P*_e_ < 5 × 10^–6^ cm/s). Thus, an amide-to-ester substitution improves permeability and offers more flexibility in compound design as a wider range of ester linkers are more likely to be permeable than their amide counterparts. This design flexibility is crucial since small modifications to the linker can significantly affect PROTAC bioactivity, ternary complex formation, and subsequent targeted degradation.^3,54,55^

### PROTACs Exhibit Ligand-to-Linker Intramolecular Hydrogen Bonds

The characteristic structure of PROTACs, two small molecules connected by a flexible linker, lends itself to the formation of intramolecular hydrogen bonds (IMHBs). This important feature allows for polar atoms to be shuttled across the lipophilic cell membrane. It is difficult to determine the presence of IMHBs by inspecting the two-dimensional (2D) chemical structure alone. However, measuring the lipophilic permeability efficiency (LPE) of matched pairs can indicate differences in the number of exposed HBDs.^31,41^ LPE is a metric that balances aqueous solubility (calculated ALog *P*) and membrane partitioning (experimental Log *D*_(dec/w)_) to determine the efficiency with which a compound crosses a membrane at a given lipophilicity. Similar to the previously developed ΔLog *P* metric,^47,56−58^ LPE is particularly valuable in determining differences in solvent-exposed HBDs between compounds. Compounds with similar LPE values are likely to have the same number of solvent-exposed HBDs, while a ΔLPE of 1.8 suggests the difference of a single exposed HBD (compounds with higher LPE values have fewer exposed HBDs).^41^

For the majority of the liposcan compound pairs (**2**–**6** vs **9–13**, respectively), the ester compounds had higher LPEs than their counterpart amide compounds (Table 1). The ΔLPEs of between 1.1 and 1.8 suggest that the additional HBD in the amide compounds is partially to fully solvent-exposed. Interestingly, the amide compounds with an ether oxygen five atoms away from the amide −NH had similarly low ΔLPEs (cf. **1** vs **8**, **15** vs **18**, and **17** vs **20**; Table 1). Consistent with the previous work,^14,31,59^ this suggests that the amide −NH is making an IMHB with the ether oxygen in the PEG linker (**15** and **17**) or the OMe ether oxygen of a POI-ligand mimic (**1**). These results are also consistent with the recent work from Kihlberg et al., who used NMR to show IMHB between PROTAC warheads and the oxygen atoms in their PEG linkers.^25^ Therefore, while ester bonds and alkyl linkers are better for permeability, when used in combination, a PEG linker and amide bond could be used to shield the polarity of important HBDs that are crucial to the bioactivity or solubility of the overall molecule.

### Esters Maintain Plasma Stability and Binding to the VHL E3 Ligase

While amide-to-ester substitutions offer increased permeability, leading to increased flexibility in compound design, esters are also typically more susceptible to plasma-mediated hydrolysis, which can lead to low *in vivo* efficacy.^60^ For these amide-to-ester substitutions to be a viable option in drug development, it is crucial to compare the stability of ester and amide compounds. We incubated **1**–**20** in human plasma at 37 °C for 0, 15, 30, and 90 min to test this. Overall, the amides were more stable in plasma than their ester counterparts. This effect was more pronounced for compound pairs with smaller, sterically unhindered POI-ligand mimics, with ≤10% compound loss of the amide compounds at 90 min (**1–4**) compared to 60–90% compound loss at 90 min for their ester counterparts (**8–11**) (Table 1 and SI Figure 1). Esters **12–14** contained larger warheads with likely more steric shielding around the susceptible ester. These compounds had much lower compound loss after 90 min (4–10%; Table 1 and SI Figure 1). This reduced hydrolysis, evident with bulky substituents, suggests that amide-to-ester substitutions could be used to increase PROTAC permeability without affecting PROTAC *in vivo* or *in cellulo* activity as these larger substituents more closely represent typical POI ligands present in PROTACs.

Maintaining target binding affinity is another crucial feature to consider while optimizing PROTACs for improved permeability. As previously mentioned, an amide-to-ester substitution between the linker and the VHL ligand decreased binding affinity by about 2-fold.^31^ In this work, we used a similar fluorescence polarization (FP) competition binding assay to determine if the amide-to-ester substitution between the linker and the POI-ligand mimic was more tolerated when binding to the VHL protein. Using our second amide (**3**, **15**–**17**) and ester (**10**, **18**–**20**) series, composed of varying linker lengths, we found that both amides and esters had FP-derived dissociation constants (*K*_d_) that were broadly comparable to each other at each linker length (SI Figure 2). The amides appeared to show slightly better binding at each linker length compared with their ester counterparts, yet the *K*_d_ values were roughly within the error of each pair. Interestingly, changes in linker length had a more pronounced effect on VHL binding than the amide-to-ester modification. The two compounds with alkyl linkers, **3** and **10**, had *K*_d_ values (119 and 136 nM, respectively) more similar to VH032 alone (113 nM). Binding affinity was slightly reduced for all compounds containing PEG-based linkers, with 1-PEG and 2-PEG units (amides **15–16***K*_d_ ≈ 170 nM and esters **18–19***K*_d_ ≈ 200 nM, respectively) showing comparable binding affinity. The longer 3-PEG unit compounds (**17** and **20**) showed slight recovery, with *K*_d_ values (138 and 144 nM, respectively) closer to their alkyl chain counterparts **3** and **10** (119 and 136 nM, respectively) for both amide and ester compounds (SI Figure 2). However, the *K*_d_ values for all compounds in this linker series (either amide or ester) were within 2-fold of the VH032 ligand alone. Encouragingly, this suggests that, for a given linker, an amide-to-ester substitution away from the E3 ligand will have little to no effect on E3 binary binding.

### Applying Model Compound Findings to a PROTAC Library

With this model toolkit for improving PROTAC permeability in hand, we were curious to determine if we could apply these insights to improve PROTAC permeability and, as a result, degradation activity. To test this idea, we decided to study two previously published and structurally similar BET-targeting PROTACs, MZ1 (**21**)^3^ and ARV-771 (**22**).^38^ MZ1 is composed of a pan-selective triazolothienodiazepine BET inhibitor, (+)-JQ1,^61^ connected to the VHL ligand VH032 via a 3-PEG-based linker. ARV-771 uses the same BET-targeting ligand but differs from MZ1 by having a slightly shorter, more lipophilic linker (minus CH_2_-O) and containing an extra chiral methyl group at the benzyl position of the VHL ligand. Because both esters and amides at the linkage point of JQ1 are equally effective at binding to BET bromodomains,^61−63^ we reasoned that MZ1 and ARV-771 would provide an ideal model system to study the effect of the amide-to-ester substitution without interfering with binary POI binding affinity. Using a combination of amide-to-ester substitutions between JQ1 and the linker, and subtle modifications to linker length and composition, we designed and synthesized compounds **23**–**28** (Table 2) with a goal to improve the degrader activity through increased permeability.

**Table 2:**
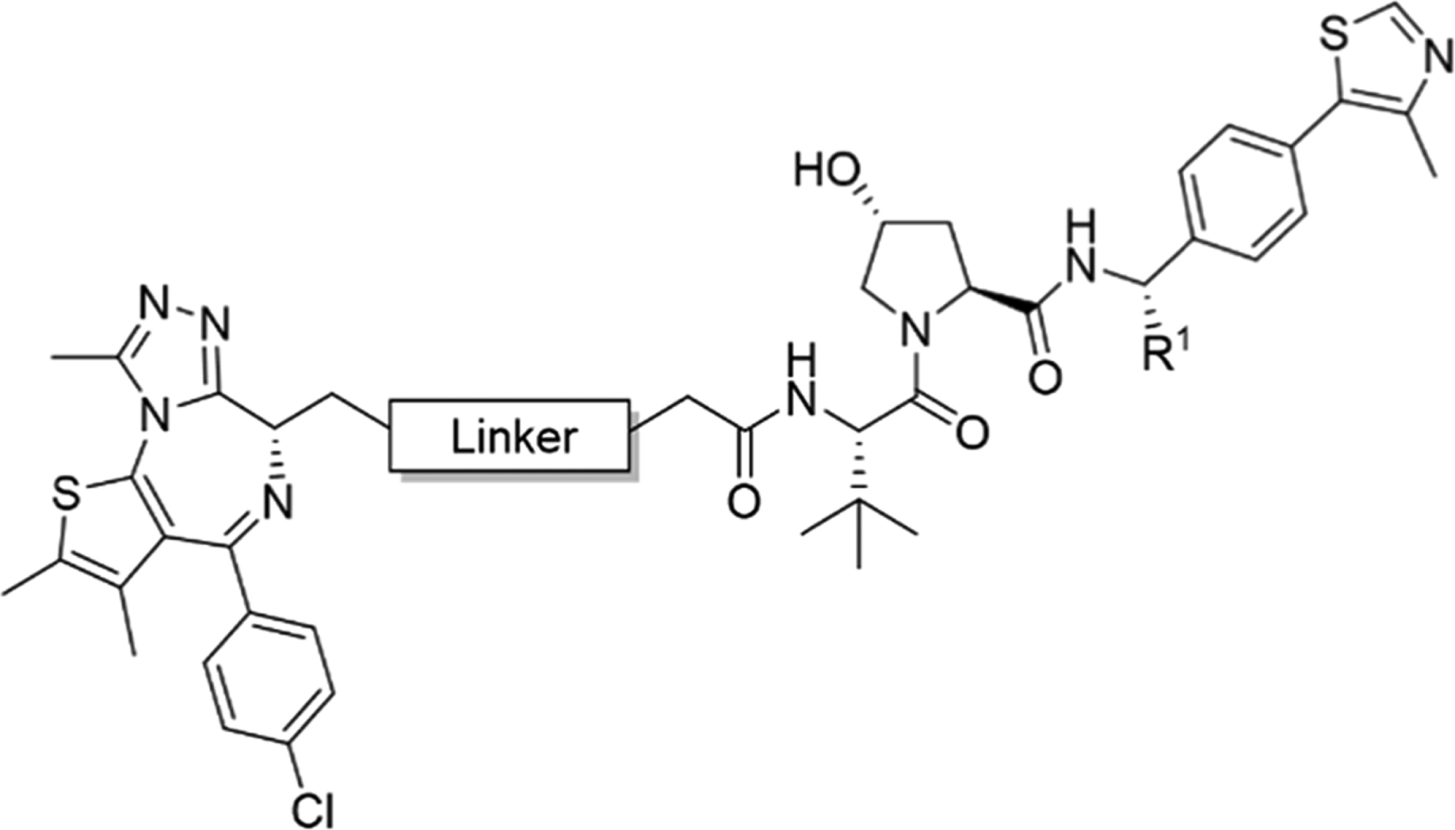

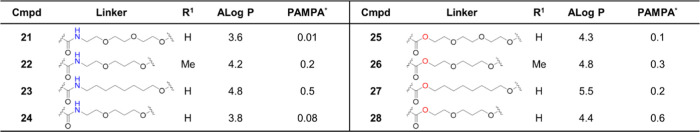
PROTAC Toolbox^a^

a Chemical structures, calculated lipophilicity (ALog *P*), and PAMPA permeabilities for **21–28** including existing BET degraders, MZ1 (**21**) and ARV-771 (**22**). *PAMPA *P*_e_ values are ×10^–6^ cm/s.

### Improving PROTAC Permeability Increases PROTAC Bioactivity

**Figure 4:**
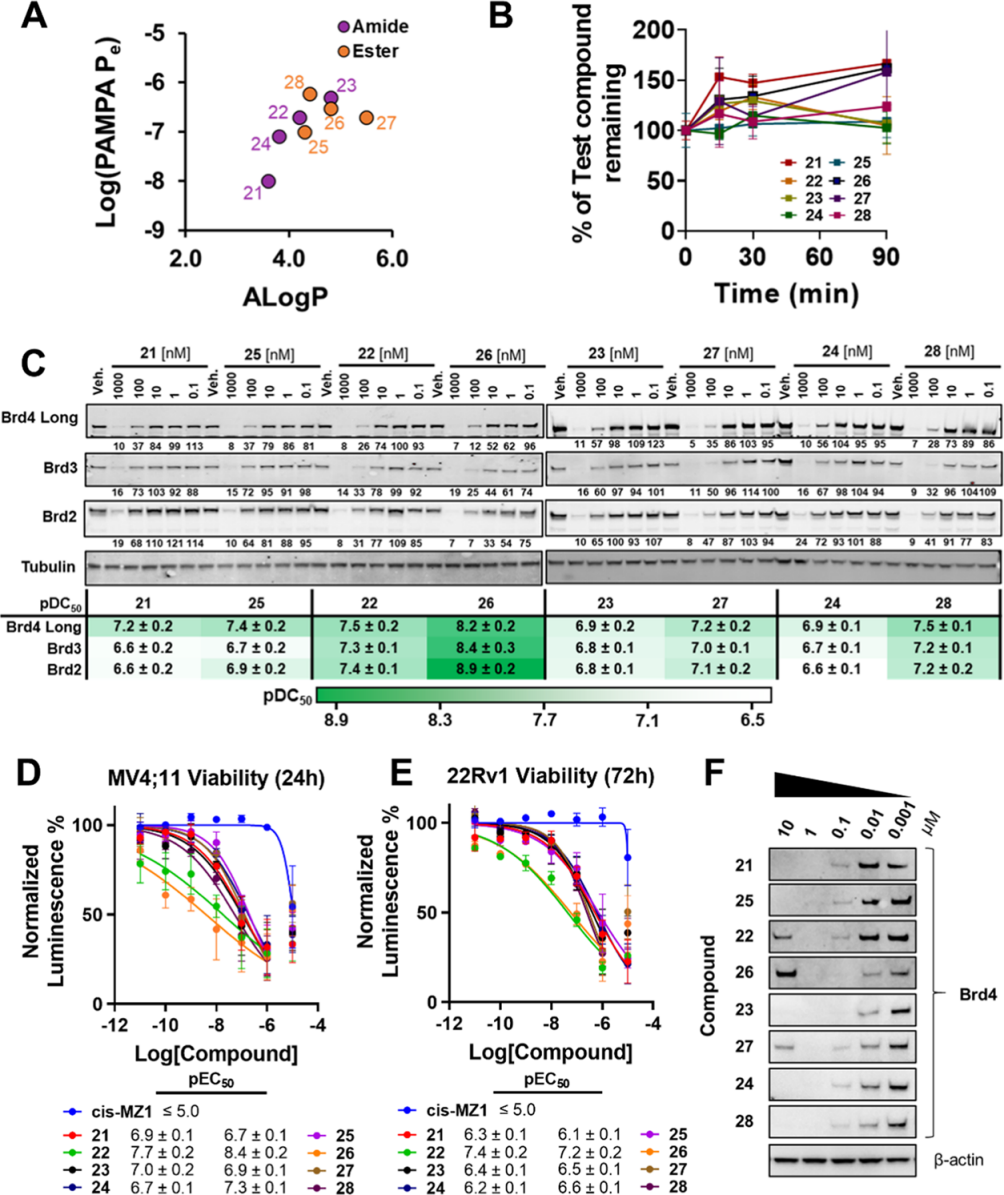
PROTAC permeability, stability, and cellular activity. (A) Permeabilities of PROTACs **21**–**28** compared with calculated lipophilicity (ALog *P*); (B) percent of PROTAC remaining after 0, 10, 30, and 90 min in human plasma at 37 °C, normalized to the 0 min time point. (C) Cellular activity of PROTACs **21**–**28**. Western blot data for BET protein levels monitored from 1 μM to 100 pM compound treatment over 4 h in HEK293 cells. Bands were normalized to vehicle control (dimethyl sulfoxide (DMSO)) and tubulin. pDC_50_ values (±S.E.) are mean from three independent experiments. (D, E) Antiproliferation of PROTACs **21**–**28** and nondegrader control cis-MZ1. MV4;11 (D) and 22Rv1 (E) cells were treated with varying concentrations of compounds and, after 24 and 72 h, respectively, were subject to CellTiter-Glo cell viability assay. pEC_50_ values (±S.E.) are mean from *N* = 3 for MV4;11 and *N* = 2 for 22Rv1. (F) Hook effect shown from Western blot data for Brd4 protein levels monitored from 10 μM to 1 nM compound treatment over 4 h in HEK293 cells, *N* = 1.

Overall, the PAMPA permeabilities of our new PROTAC series followed the same trends shown by our model compounds. In three of the four amide-to-ester matched pairs (MZ1 (**21**) and OMZ1 (**25**), ARV-771 (**22**) and OARV-771 (**26**), and AB2 (**24**) and OAB2 (**28**)), the amide-to-ester substitution led to an increase in permeability by 10-, 1.5- and 7.5-fold, respectively (Table 2, Figure 4A, and SI Table 3). As expected, substituting the amide in AB1 (**23**) for an ester in OAB1 (**27**) caused a 2.5-fold reduction in permeability in the last matched pair. This decrease in permeability is due to the increased lipophilicity of **27**, ALog *P* = 5.5, pushing the ester into the insoluble ALog *P* regime. Compound **28** had the highest PAMPA permeability (0.6 × 10^–6^ cm/s), with an ALog *P* of 4.4. PROTACs with ALog *P* values > 4.4 started to show a decrease in PAMPA permeability (Figure 4A). Compounds **22** and **26** contained an extra chiral methyl group at the benzyl position of the VH032 ligand. The effects of this additional methyl group on permeability can be seen when comparing two alternative matched pairs within the amide series, **22** vs **24**, and within the ester series, **26** vs **28**. In the amide pairing, an additional methyl group increases PAMPA permeability by 2.5-fold, whereas, in the case of the ester pairing, permeability decreases by 2-fold. Again, these trends are likely the result of the well-established “inverted-parabola” relationship between ALog *P* and permeability.^64−66^ As MDCK permeability measurements are less sensitive to poor compound solubility than PAMPA permeability at high lipophilicities,^41,64^ we attempted to collect MDCK cell permeabilities for these PROTAC compounds starting with **21** and **25**. However, both compounds were below the limit of detection in the apical to basal permeation (SI Table 2). Thus, we did not pursue MDCK permeabilities on the remaining PROTACs. Taken together, our permeability data are consistent with effects caused by increasing lipophilicity and reducing PEG-like character of the PROTAC linker, producing similar trends in permeability, as described for the model compounds above. Furthermore, all eight PROTACs were stable in plasma after 90 min, with no detectable reduction in PROTAC levels (Figure 4B). This suggests that a rigid and sterically bulky POI-ligand, like JQ1, provides sufficient protection from ester hydrolysis, as also suggested by the model compounds.

Next, we evaluated the cellular activities of all eight PROTACs in HEK293 cells to obtain a degradation (DC_50_) profile for BET proteins, Brd4, Brd3, and Brd2 (Figure 4C, SI Table 5, and SI Figures 3 and 4). Notably, the moderate improvements in permeability seen when substituting the amide in known degraders **21** and **22** for an ester in **25** and **26** translated into overall improvements in bioactivity. Compound **25** showed a 1.5- to 2-fold increase in degradation potency over **21** for both Brd4 (DC_50_ = 44 vs 60 nM, respectively) and Brd2 (DC_50_ = 133 vs 230 nM, respectively), while **25** showed near-equipotent degradation compared to **21** for Brd3 (DC_50_ = 221 vs 239 nM, respectively). Strikingly, **26** showed to be the most potent degrader out of this series, with a 5.5-fold more potent degradation of Brd4 compared to its amide counterpart, **22** (DC_50_ = 6 vs 33 nM, respectively), a 42-fold increase for Brd2 (DC_50_ = 1 vs 42 nM, respectively) and a 12-fold increase for Brd3 (DC_50_ = 4 vs 47 nM, respectively). Similarly, **27** gave a 2-fold increase in degradation potency over its amide counterpart, **23**, for both Brd4 (DC_50_ = 133 vs 57 nM, respectively) and Brd2 (DC_50_ = 87 vs 166 nM, respectively), and also, a slight 1.5-fold increase with Brd3 (DC_50_ = 107 vs 158 nM, respectively). Finally, **28** showed a 4-fold increase in degradation potency against Brd4 when compared to its amide counterpart, **24** (DC_50_ 31 vs 125 nM, respectively), a 4-fold increase with Brd2 (DC_50_ = 68 vs 273 nM, respectively), and a 3.2-fold increase with Brd3 (DC_50_ = 68 vs 273 nM, respectively) (Figure 4C, SI Table 5, and SI Figures 3 and 4). Together, the cellular degradation data demonstrate that the amide-to-ester substitution has a beneficial effect on PROTAC activity.

We and others have shown that the improved PROTAC-induced degradation of BET proteins translates to enhanced effects on the viability of BET-dependent cancer cell lines.^38,67^ We therefore evaluated the cytotoxicity of our PROTAC series by assessing the viability of BET-sensitive cancer cell lines MV4;11 (acute myeloid leukemia) (Figure 4D and SI Table 5) and 22Rv1 (human prostate carcinoma) (Figure 4E and SI Table 5). All PROTACs exhibited a marked antiproliferative effect on each cell line, consistent with their activity as degraders. Compounds **22** and **26** gave the most pronounced effect, with EC_50_ values of 18 and 4 nM in MV4;11, respectively, and 44 and 58 nM in 22Rv1, respectively. Notably, out of the nonmethylated VH032-based PROTACs, **28** was the most effective, with EC_50_ values of 53 and 250 nM for MV4;11 and 22Rv1, respectively. This compound had the highest PAMPA permeability (*P*_e_ = 0.6 × 10^–6^ cm/s; Table 2 and SI Table 3), suggesting that it permeates membranes more effectively and is thus able to start the catalytic cycle of ternary complex formation, ubiquitination, and degradation at lower compound dose, leading to increased cell antiproliferation. Furthermore, it is because of this catalytic activity at substoichiometric concentrations that even a modest improvement in permeability can significantly increase a PROTAC’s degradation activity and cytotoxicity.

Interestingly, in MV4;11 cells, all of the PROTACs became less effective at higher concentrations (10 μM) (Figure 4D). This was ascribed to be due to the “hook effect”,^68^ a well-known phenomenon displayed by bifunctional PROTAC degraders, where at high concentrations, unproductive binary complexes of PROTAC:E3 ligase and of PROTAC:POI outcompete ternary complex formation (POI:PROTAC:E3 ligase). Moreover, in 22Rv1 cells, all PROTACs, with the exceptions of **21**, **24**, and **25**, exhibited a hook effect to varying degrees (Figure 4E). Interestingly, **22**, **23**, **26**, **27**, and **28** all have PAMPA permeabilities ≥0.2 × 10^–6^ cm/s and generally higher ALog *P* values than **21**, **24**, and **25**. This suggests that **22**, **23**, **26**, **27**, and **28** likely enter the cell more efficiently, leading to higher intracellular PROTAC concentrations and thus more pronounced hook effects. On broader terms, this correlation between PAMPA permeabilities and the hook effect further supports that the PAMPA permeabilities measured with our PROTAC series translate into relevant trends in their cellular activity profiles.

To further evaluate the observed hook effect seen in the cell viability assay, we decided to orthogonally investigate this in cell degradation assays by Western blot, assessing Brd4 protein levels in HEK293 cells starting with a 10 μM treatment of PROTAC (Figure 4F). Strikingly, ester compounds **26** and **27**, and also amide **22**, exhibited a hook effect at 10 μM. Compounds **22** and **26** both possess an extra methyl on the VH032 ligand, which enhances binary binding affinity to VHL. A possible explanation for the observed onset of the hook effect in **22** and **26** is that their stronger binding to VHL contributes to the binary complex being more effective at outcompeting the ternary complex formation. Alternatively, this could also be attributed to the increased lipophilicity and permeability conferred by the added methyl group (cf. **22** vs **24** and **26** vs **28**; Table 2 and SI Table 3). Similarly, **26** appears to hook to a greater extent than its amide counterpart **22**, suggesting that intracellular concentrations of **26** are higher, most likely due to the increase in lipophilicity and PAMPA permeability (cf. Table 2, Figure 4A, and SI Table 3). Interestingly, **27** is the only nonmethylated VH032-based compound that exhibited the hook effect. This could be due to **27** being the most lipophilic compound out of the series (ALog *P* = 5.5).

### Improved Potency Is Due to Improved Permeability Rather Than Improvements to Ternary Complex Formation

**Figure 5:**
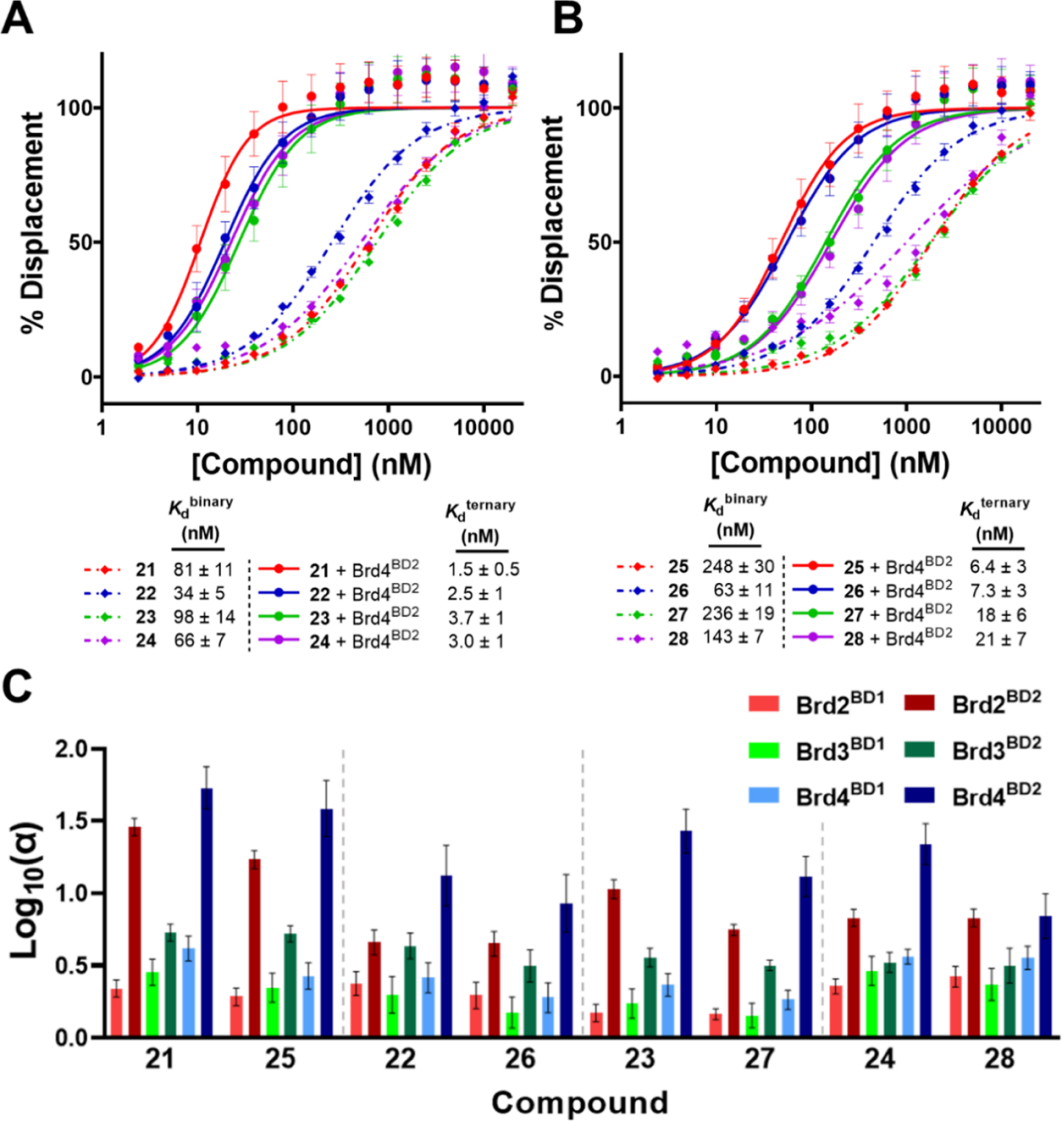
Fluorescence polarization (FP) of PROTAC binding. Binary and ternary complex formation FP data for amide (A) and ester (B) PROTACs to VHL alone (diamonds and dashed line) or preincubated Brd4^BD2^ with PROTAC to VHL (circles and solid line). *K*_d_ values are mean ± standard error of the mean (SEM) from *N* = 5–6 for binary binding to VHL and *N* = 3 for ternary binding. Left-shift between binary and ternary data indicates positive cooperativity. FP binding data for the remaining five BET proteins can be found in the Supporting Information. (C) Cooperativity (α) is plotted as Log_10_(α) (± propagated uncertainty). Gray dashed lines separate amide-to-ester matched pairs.

We have previously shown that the PROTAC MZ1 forms highly cooperative, stable, and long-lived ternary complexes with BET bromodomains/MZ1 and displays a preference for second bromodomains (BD2s) over first bromodomains (BD1s), particularly for Brd4^BD2^, and these biophysical characteristics of the ternary complex underpin a high level of target ubiquitination and drive potent and fast degradation activity of MZ1 with Brd4.^14,34,69^ We thus wondered to what extent the improvements in cellular activity that we observed with our set of PROTACs might be contributed from the more favorable ternary complex formation. To address this question, we biophysically characterized all compounds in our PROTAC series by measuring both binary binding to VHL and ternary complex formation between VHL, PROTAC, and both BD1 and BD2 bromodomains of Brd4, Brd3, and Brd2 (Figure 5 and SI Table 4). We measured cooperativity across the entire set of eight PROTACs vs six bromodomains (48 combinations). We used a competitive FP assay in which a fluorescently labeled HIF-1α peptide probe bound to VHL is displaced by titrating either PROTAC alone (for binary binding) or PROTACs preincubated with individual BET bromodomains (for ternary complex binding). This allows us to calculate the cooperativity (α) of ternary complex formation (α = *K*_d_^binary^/*K*_d_^ternary^; Figure 5C).^14,34,67,70,71^

Strikingly, in all amide-to-ester matched pairs, the esters were 2- to 3-fold weaker at binding to VHL, with the largest difference being between **21** (*K*_d_ = 81 nM) and **25** (*K*_d_ = 248 nM). As expected, due to the additional benzyl methyl group present in the VH032 ligand,^72^ PROTACs **22** and **26** showed the strongest binary binding to VHL (*K*_d_ = 34 and 63 nM, respectively). Additionally, all esters were also 2- to 7-fold weaker than their amide counterparts at binding VHL when prebound to each individual BET bromodomain, as measured by their ternary *K*_d_ values (cf. Figure 5B vs 5A). One hypothesis for this decreased binding affinity is the formation of a new IMHB between the new HBA present in the ester group and a HBD of an amide in the VH032 ligand. This could cause the rigid and relatively bulky JQ1 ligand to sit on top of the VHL protein, potentially causing unfavorable clashes and requiring an energetic penalty to allow binding to VHL. This is somewhat evident when switching from a PEG linker in **25** to a more alkyl linker in **27**, which has negligible effects on VHL binding; however, shortening the linker in **28** gave a 1.75-fold increase in affinity. The shorter linker decreases flexibility for the molecule to fold and form new IMHBs. Recently, others have shown how VHL targeting PROTACs can fold and form IMHBs in different solutions to change their TPSA.^25^ Further structural studies are warranted to fully assess this phenomenon in the PROTACs presented here.

In all cases, PROTACs formed preferential and more positively cooperative ternary complexes with the second bromodomain (BD2) of each BET protein over the first bromodomain (BD1), consistent with what is observed with MZ1 (see refs (14, 34) and data herein; cf. **21**). All PROTACs displayed cooperative ternary complexes with all BET BDs (α > 1; Figure 5C and SI Table 4). Interestingly, all esters, albeit retaining positive cooperativity with each BET BD, were slightly less cooperative than their amide counterparts. Ternary complexes between Brd4^BD2^, PROTAC, and VHL formed the strongest and most cooperative ternary complex, as in the case of MZ1 (**21**).^14,34^ This can be seen by a left-shift in the FP displacement curve when the bromodomain is present (Figure 5A,B). Interestingly, **21**, **25**, **23**, and **27**, which all have the same linker length, follow the same intra-BET bromodomain cooperativity profile (Figure 5C), suggesting that these compounds form similar ternary complexes to one another. In contrast, compounds **22**, **26**, **24**, and **28**, which all contain the same, shorter linker, were found to be less discriminatory between individual bromodomains. Based on these results, we conclude that these four compounds likely form ternary complexes that, while similar to one another, are significantly different from the structurally resolved complex of MZ1 (**21**).^14^ Importantly, within these sets of compounds with the same linker length, each amide-to-ester matched pair showed an identical intra-BET selectivity profile, strongly suggesting that each pair forms a highly similar ternary complex.

Noticeably, MZ1 (**21**) formed the strongest Brd4^BD2^:PROTAC:VHL complex (*K*_d_^ternary^ = 1.5 nM, α = 54), values comparable to earlier work by Roy et al. (α = 55),^34^ with **28** forming the weakest (*K*_d_^ternary^ = 21 nM, α = 7) out of the series. When comparing MZ1 (**21**) and ARV-771 (**22**), **22** showed near-equipotent ternary binding to **21** (*K*_d_^ternary^ = 2.5 nM). However, the complex induced by **22** was 4-fold less cooperative (α = 13) than the complex induced by **21** (α = 54). Despite this reduced cooperativity, **22** was a more potent degrader across all three BET domains and was more cytotoxic in both cell lines than **21** (Figure 4C–E and SI Table 5). However, due to the expected differences in ternary complexes formed by **22** and **21**, it is difficult to dissect whether the major factor in the improved cellular activity of **22** over **21** is a 2-fold higher VHL binding affinity (*K*_d_^binary^ = 34 and 81 nM, respectively) or the 20-fold increase in membrane permeability of **22** over **21** (*P*_e_ = 0.2 × 10^–6^ cm/s and 0.01 × 10^–6^ cm/s, respectively; Table 2 and SI Table 3). Either way, this does suggest that some combination of increased VHL binding and membrane permeability can compensate for reduced cooperativity, albeit at the expense of an earlier onset of the hook effect.

The similar biophysical profiles of the amide–ester matched pairs allow a more robust assessment of the contribution of cell permeability to PROTAC degradation activity for these compounds. Analysis of these matched pairs makes it clear that increased protein degradation for these compounds must be driven by increased cell permeability rather than ternary complex formation. For example, the ester matched pair of MZ1 (cf. **25** and **21**), **25**, was found to be 1.5-fold more potent than **21** at degrading Brd4 (Figure 4C and SI Table 5), despite **25** having 3-fold weaker binary and ternary affinities to VHL ± Brd4^BD2^ than **21** and also forming a less cooperative complex (α = 39 vs 54, respectively). Therefore, it is evident that the moderate increase in potency is derived from the 10-fold increase in membrane permeability of **25** compared to that of **21** (Figures 4 and 5). Similarly, the ester matched pair of ARV-771 (cf. **26** and **22**), **26**, displayed a 5.5-fold increase in Brd4 degradation potency relative to **22**, despite having 2- to 3-fold weaker binary and ternary affinities to VHL ± Brd4^BD2^ than **22** and also forming a less cooperative ternary complex (α = 8.5 vs 13, respectively). Finally, ester **28** not only has the highest PAMPA permeability (*P*_e_ = 0.6 × 10^–6^ cm/s) of the entire PROTAC series and is 7.5-fold more permeable than its amide counterpart, **24**, but **28** also displays the most potent degradation of Brd4 in cells when compared with the other VH032-based PROTACs within the series (**21**, **25**, **23**, **27**, and **24**). This is despite **28** having the lowest ternary affinity and cooperativity with Brd4^BD2^ (*K*_d_^ternary^ = 21 nM, α = 7).

These observations suggest that the improvements in protein degradation with these compounds must be driven by cell permeability rather than ternary complex formation. Indeed, for each matched pair, the ester is more permeable than the parent amide: **25** is more lipophilic and 10-fold more permeable than **21**; **26** is more lipophilic and 1.5-fold more permeable than **22**; and **28** has the highest PAMPA permeability (*P*_e_ = 0.6 × 10^–6^ cm/s) of the entire PROTAC series and is 7.5-fold more permeable than **24** (Table 2, Figure 4A, and SI Table 3). These results also highlight the utility of PAMPA to ascertain biologically meaningful permeability differences among PROTACs, even among compounds whose absolute permeabilities are very low (<10^–6^ cm/s). Taking these data altogether, it is evident that the greater activity of ester PROTACs relative to their amide counterparts is being influenced by their ability to permeate into cells more efficiently, thus, initiating the ternary complex-driven catalytic knockdown of target BET proteins at overall lower doses, resulting in more potent degrader compounds.

## Conclusions

Composed of two binders and a linker, bifunctional PROTAC degraders typically fall in the bRo5 space.^26^ Chemists have recently been pushed to shift away from designing molecules in the traditional Ro5 chemical space^46,73^ as more bRo5 compounds are shown to be cell-active, and developable *in vivo*, including being orally bioavailable.^28,30^ However, having general guidelines for physicochemical design parameters for bRo5 compounds like PROTACs is critical to improving their chances to be useful cellular probes and to be developable as drugs.^53^ In response to this pressing need, some have attempted to improve permeability, solubility, and efflux ratios through linker modifications.^71^ In contrast, others have also attempted to reduce the number of amide bonds (HBDs) to improve the physicochemical properties of PROTACs.^74^ We and others have attempted to develop systematic studies of PROTAC physicochemical properties and new methods to study these properties.^22,25,31−33^

In this study, we have shown that PAMPA is a reliable predictor of PROTAC permeability that translates relatively well into their cellular activity profiles. This has allowed us to develop strategies for improving PROTAC potency by improving permeability despite the previously suggested propensity of PROTACs to actively undergo efflux. We used a systematic investigation of linker lengths and lipophilicity combined with amide-to-ester substitutions to improve the permeability of “PROTAC-like” model compounds. We demonstrated that the PROTACs studied herein achieve the highest permeability at moderate lipophilicities (3–5) and that, within this range, increasing the lipophilicity of a compound leads to increased permeability, as has been seen with other beyond Ro5 compounds.^41^ Designing compounds in this range (which we have found to contain more permeable compounds) is also likely to reduce toxicity.^53^ We also demonstrate that amide-to-ester substitutions can increase PROTAC permeability in this ALog *P* range as well. Therefore, ester-containing compounds in this lipophilicity range are likely to have better overall pharmacokinetic properties than amide compounds or those with higher lipophilicities. Finally, though esters are more prone to hydrolysis and therefore tend to be less stable in plasma, we discovered that adding steric bulk to the chemical space surrounding the area (i.e., near the warhead) drastically reduces compound degradation in the plasma. Therefore, amide-to-ester substitutions remain a viable option for PROTAC pharmacokinetic improvement, leading to more compounds reaching their intracellular target. In each amide-to-ester PROTAC matched pair, we have demonstrated that this simple functional group conversion can lead to significant increases in PROTAC bioactivity, despite esters showing weaker binding affinity than their amide counterparts. We therefore provide what are, to the best of our knowledge, unprecedented examples of optimizing PROTAC degradation activity through systematic and rational improvements in compound cell permeability. It is clear that the increase in lipophilicity and permeability shown by the esters and linker-modified compounds relative to amides has a positive effect on cellular activity and should be considered when designing future degraders while attempting to retain favorable productive ternary complex formation. Amide-to-ester substitution thus provides a simple and convenient bioisosteric replacement that we anticipate will find wide utility as an attractive strategy for the development and optimization of PROTACs, as well as other emerging beyond Ro5 compounds of chemically induced proximity.^75−77^

## Experimental Section

### Chemistry General

Unless otherwise stated, purchased solvents and reagents were used without further modification. Solvents were purchased from Fisher Scientific. General reagents were purchased from Fisher Scientific except for the following: HATU (Chem-Impex or Combi-Blocks), amino acids and linkers (Combi-Blocks or Oakwood), and SynPhase polystyrene lanterns (Mimotopes). Purity is greater than 95% for all compounds tested biologically. Model compounds were purified on a Biotage Isolera Prime with a SNAP Ultra C18 25 g column using a gradient of 10–100% acetonitrile in water with 0.1% trifluoroacetic acid (TFA) at a flow rate of 25 mL/min. PROTAC intermediates were purified by flash column chromatography using a Teledyne Isco Combiflash Rf or Rf200i with Normal Phase RediSep Rf Disposable Columns or with Reverse Phase RediSep Rf Gold C18 Reusable Columns. Final PROTAC compounds were purified by high-performance liquid chromatography (HPLC) using a Gilson Preparative HPLC System equipped with a Waters XBridge C18 column (100 mm × 19 mm; 5 μm particle size) using a gradient from 5 to 95% of acetonitrile in water containing 0.1% formic over 10 min at a flow rate of 25 mL/min unless stated otherwise.

A Thermo Scientific Ultimate 3000 UPLC system and Thermo Scientific Orbitrap VelosPro mass spectrometer were used to run liquid chromatography-mass spectrometry (LC/MS)-based assays (PAMPA and Log *D*_(dec/w)_) eluting with 5–95% ACN in H_2_O with 0.1% formic acid. This system was fitted with a Thermo Hypersil GOLD C18 (30 mm × 2.1 mm, 1.9 μm particle size) column. LC/MS purity traces of the model compounds were collected using a Thermo Finnigan Surveyor HPLC system and Thermo Fisher Scientific Finnigan LTQ mass spectrometer. These samples were eluted with 10–100% ACN in H_2_O with 0.1% formic acid on an Agilent Poroshell 120 EC-C18 (30 mm × 2.1 mm, 2.7 μm particle size). NMR samples on model compounds were collected on a Bruker 500 MHz NMR with a 5 mm BBO Smart Probe in deuterate chloroform unless otherwise stated. For the PROTAC compounds and synthetic intermediates, compound characterization using NMR was performed on either Bruker 500 Ultrashield or Bruker Ascend 400 spectrometers. The proton (^1^H) and carbon (^13^C) reference solvents used were as follows: d1-chloroform–CDCl_3_ (δH = 7.26 ppm/δC = 77.15 ppm). Signal patterns are described as singlet (s), doublet (d), triplet (t), quartet (q), quintet (quint.), multiplet (m), broad (br.), or a combination of the listed splitting patterns. Coupling constants (*J*) are measured in Hertz (Hz). NMR spectra for all compounds were processed using Bruker TopSpin 4.1.0.

For PROTAC intermediates and final PROTAC compounds, reactions were monitored using an Agilent Technologies 1200 series analytical HPLC connected to an Agilent Technologies 6130 quadrupole LC/MS containing an Agilent diode array detector and a Waters XBridge C18 column (50 mm × 2.1 mm, 3.5 μm particle size). Samples were eluted with a 3 min gradient of 5–95% acetonitrile: water containing 0.1% formic acid at a flow rate of 0.7 mL/min. High-resolution mass spectrometry (HRMS) data were performed on a Bruker MicrOTOF II focus ESI Mass Spectrometer connected in parallel to a Dionex Ultimate 3000 RSLC system with a diode array detector and a Waters XBridge C18 column (50 mm × 2.1 mm, 3.5 μm particle size). Samples were eluted with a 6 min gradient of 5–95% acetonitrile: water containing 0.1% formic acid at a flow rate of 0.6 mL/min.

Synthesis of new compounds and their intermediates is described in the Chemistry General section. VH032,^42^ VH032-NH_3_Cl,^42^ Me-VH032-NH_3_Cl,^72^ MZ1 (**22**),^3^ and cis-MZ1^3^ were all synthesized using literature procedures. Synthesis of ARV-771^38^ was adapted from the literature procedures—new intermediates are characterized in the Chemistry General section. Enantiopure (+)-JQ1 as tBu ester was purchased from Advanced ChemBlocks Inc. (Cat. ID L14965), which was hydrolyzed with TFA to yield (+)-JQ1 carboxylic acid in quantitative yields.

#### Procedure for Loading SynPhase Polystyrene l-Series Lanterns

To load the lanterns, (9*H*-fluoren-9-yl)methyl(2*S*,4*R*)-4-hydroxy-2-((4-(4-methylthiazol-5-yl)benzyl)carbamoyl)pyrrolidine-1-carboxylate (**29**) was synthesized and conjugated onto SynPhase polystyrene l-series lanterns (alkyl-tethered diisopropylarylsilane linker, 22 μM, catalogue # MIL10431000) following previously published protocols to generate compound **30**.^31,42,78^ Compound **31** was synthesized according to Klein et al.^31^ on SynPhase lanterns using Fmoc deprotection and an addition of Fmoc-Tle-OH with HATU and *N*,*N*-diisopropylethylamine (DIPEA) in *N*,*N*-dimethylformamide (DMF).

#### Solid Phase Synthesis of Compounds **1**–**7**

Compounds **1–7** were synthesized using solid phase synthesis on SynPhase polystyrene lanterns. The procedure is described for synthesis on one lantern each for **1–7**. A lantern with **31** was Fmoc-deprotected using 2 mL of a solution of 2% piperidine and 2% DBU in DMF for 15 min at room temperature. The deprotection solution was drained, and the lantern was rinsed 3× with 2 mL of DMF and 3× with 2 mL of dichloromethane (DCM, 30 s per wash). Next, a solution of Fmoc-6-aminohexanoic acid (31 mg, 0.088 mmol, 4 equiv), HATU (34 mg, 0.088 mmol, 4 equiv), and DIPEA (613 μL, 0.176 mmol, 8 equiv) in 3 mL of DMF was added to the lantern. The reaction was mixed on a linear shaker at room temperature for 4–16 h. The reaction mixture was drained, and the lantern was rinsed 3× with 2 mL of DMF and 3× with 2 mL of DCM (30 s per wash). The lantern was Fmoc-deprotected using 2 mL of a solution of 2% piperidine and 2% DBU in DMF for 15 min at room temperature. The deprotection solution was drained, and the lantern was rinsed 3× with 2 mL of DMF and 3× with 2 mL of DCM (30 s per wash). Then, a solution of the capping agent (listed below) (0.088 mmol, 4 equiv), HATU (34 mg, 0.088 mmol, 4 equiv), and DIPEA (613 μL, 0.176 mmol, 8 equiv) in 3 mL of DMF was added to the lantern. The reaction was mixed on a linear shaker for 4–16 h at room temperature. Capping agents: **1** (methoxyacetic acid, 6.8 μL), **2** (butyric acid, 8.0 μL), **3** (phenylacetic acid, 12 mg), **4** (cyclohexanecarboxylic acid, 11.3 mg), **5** (2-(adamantan-1-yl)acetic acid, 17.1 mg), **6** (3,3-diphenylpropionic acid, 19.9 mg), and **7** (4-butoxy-4′-biphenylcarboxylic acid, 23.8 mg). The reaction mixture was drained, and the lantern was rinsed 3× with 2 mL of DMF and 3× with 2 mL of DCM (30 s per wash). Compounds **1–7** were cleaved from the lantern with 5% HF/pyridine in tetrahydrofuran (THF) and quenched following previously published procedures with methoxytrimethylsilane.^78^ The quenched cleavage solution was evaporated under reduced pressure, and the compounds were purified on a Biotage Isolera Prime flash chromatography system with a 30 g C18 column eluting with 10–100% ACN in H_2_O, both with 0.1% TFA. Sample identify was confirmed by LC/MS.

#### Solid Phase Synthesis of Compounds **8**–**14**

Compounds **8–14** were synthesized using solid phase synthesis on SynPhase polystyrene lanterns. The procedure is described for synthesis on one lantern. A lantern with **31** was Fmoc-deprotected using 2 mL of a solution of 2% piperidine and 2% DBU in DMF for 15 min at room temperature. The deprotection solution was drained, and the lantern was rinsed 3× with 2 mL of DMF and 3× with 2 mL of DCM (30 s per wash). Next, a solution of 6-hydroxy-hexanoic acid (11.6 mg, 0.088 mmol, 4 equiv), HATU (34 mg, 0.088 mmol, 4 equiv), and DIPEA (613 μL, 0.176 mmol, 8 equiv) in 3 mL of DMF was added to the lantern. The reaction was mixed on a linear shaker at room temperature for 4–16 h. The reaction mixture was drained, and the lantern was rinsed 3× with 2 mL of DMF and 3× with 2 mL of DCM (30 s per wash). Then, a solution of the capping agent (listed below) (0.22 mmol, 10 equiv), *N*,*N*′-diisopropylcarbodiimide (DIC, 34 μL, 0.22 mmol, 10 equiv), and 4-dimethylaminopyridine (DMAP, 0.7 mg, 0.0055 mmol, 0.25 equiv) in 2 mL of dry DCM was added to the lantern. The reaction was mixed on a linear shaker for 4–16 h at room temperature. Capping agents: **8** (methoxyacetic acid, 6.8 μL), **9** (butyric acid, 8.0 μL), **10** (phenylacetic acid, 12 mg), **11** (cyclohexanecarboxylic acid, 11.3 mg), **12** (2-(adamantan-1-yl)acetic acid, 17.1 mg), **13** (3,3-diphenylpropionic acid, 19.9 mg), and **14** (4-butoxy-4′-biphenylcarboxylic acid, 23.8 mg). The reaction mixture was drained, and the lantern was rinsed 3× with 2 mL of DMF and 3× with 2 mL of DCM (30 s per wash). Compounds **1–7** were cleaved from the lantern with 5% HF/pyridine in THF and quenched following previously published procedures with methoxytrimethylsilane.^78^ The quenched cleavage solution was evaporated under reduced pressure, and the compounds were purified on a Biotage Isolera Prime flash chromatography system with a 30 g C18 column eluting with 10–100% ACN in H_2_O, both with 0.1% TFA. Sample identify was confirmed by LC/MS.

#### Solid Phase Synthesis of Compounds **15**–**17**

Compounds **15–17** were synthesized using solid phase synthesis on SynPhase polystyrene lanterns. The procedure is described for synthesis on one lantern. A lantern with **31** was Fmoc-deprotected using 2 mL of a solution of 2% piperidine and 2% DBU in DMF for 15 min at room temperature. The deprotection solution was drained, and the lantern was rinsed 3× with 2 mL of DMF and 3× with 2 mL of DCM (30 s per wash). Next, a solution of a linker (0.088 mmol, 4 equiv), HATU (34 mg, 0.088 mmol, 4 equiv), and DIPEA (613 μL, 0.176 mmol, 8 equiv) in 3 mL of DMF was added to the lantern. Linkers were as follows: **15** (Fmoc-6-amino-4-oxahexanoic acid, 31 mg), **16** (Fmoc-9-amino-4,7-dioxanonanoic acid, 35 mg), and **17** (Fmoc-12-amino-4,7,10-trioxadodecanoic acid, 39 mg). The reaction was mixed on a linear shaker at room temperature for 4–16 h. The reaction mixture was drained, and the lantern was rinsed 3× with 2 mL of DMF and 3× with 2 mL of DCM (30 s per wash). The lantern was Fmoc-deprotected using 2 mL of a solution of 2% piperidine and 2% DBU in DMF for 15 min at room temperature. The deprotection solution was drained, and the lantern was rinsed 3× with 2 mL of DMF and 3× with 2 mL of DCM (30 s per wash). Then, a solution of phenylacetic acid (12 mg, 0.088 mmol, 4 equiv), HATU (34 mg, 0.088 mmol, 4 equiv), and DIPEA (613 μL, 0.176 mmol, 8 equiv) in 3 mL of DMF was added to the lantern. The reaction was mixed on a linear shaker for 4–16 h at room temperature. The reaction mixture was drained, and the lantern was rinsed 3× with 2 mL of DMF and 3× with 2 mL of DCM (30 s per wash). Compounds **15–17** were cleaved from the lantern with 5% HF/pyridine in THF and quenched following previously published procedures with methoxytrimethylsilane.^78^ The quenched cleavage solution was evaporated under reduced pressure, and the compounds were purified on a Biotage Isolera Prime flash chromatography system with a 30 g C18 column eluting with 10–100% ACN in H_2_O, both with 0.1% TFA. Sample identify was confirmed by LC/MS.

#### Solid Phase Synthesis of Compounds **18**–**20**

Compounds 1**8–20** were synthesized using solid phase synthesis on SynPhase polystyrene lanterns. The procedure is described for synthesis on one lantern. A lantern with **31** was Fmoc-deprotected using 2 mL of a solution of 2% piperidine and 2% DBU in DMF for 15 min at room temperature. The deprotection solution was drained, and the lantern was rinsed 3× with 2 mL of DMF and 3× with 2 mL of DCM (30 s per wash). Next, a solution of a linker (0.088 mmol, 4 equiv), HATU (34 mg, 0.088 mmol, 4 equiv), and DIPEA (613 μL, 0.176 mmol, 8 equiv) in 3 mL of DMF was added to the lantern. Linkers were as follows: **18** (3-(2-hydroxyethoxy)propanoic acid, 12 mg), **19** (3-[2-(2-hydroxyethoxy)ethoxy]propanoic acid, 16 mg), and **20** (3-(2-[2-(2-hydroxyethoxy)ethoxy]ethoxy)propanoic acid, 20 mg). The reaction was mixed on a linear shaker at room temperature for 4–16 h. The reaction mixture was drained, and the lantern was rinsed 3× with 2 mL of DMF and 3× with 2 mL of DCM (30 s per wash). Then, a solution of phenylacetic acid (30 mg, 0.22 mmol, 10 equiv) (0.22 mmol, 10 equiv), DIC (34 μL, 0.22 mmol, 10 equiv), and DMAP (0.7 mg, 0.0055 mmol, 0.25 equiv) in 2 mL of dry DCM was added to the lantern. The reaction was mixed on a linear shaker for 4–16 h at room temperature. The reaction mixture was drained, and the lantern was rinsed 3× with 2 mL of DMF and 3× with 2 mL of DCM (30 s per wash). Compounds **18–20** were cleaved from the lantern with 5% HF/pyridine in THF and quenched following previously published procedures with methoxytrimethylsilane.^78^ The quenched cleavage solution was evaporated under reduced pressure, and the compounds were purified on a Biotage Isolera Prime flash chromatography system with a 30 g C18 column eluting with 10–100% ACN in H_2_O, both with 0.1% TFA. Sample identify was confirmed by LC/MS.

#### General Solution Phase Procedure A

Azides **45**, **47**, or **49** (1 equiv) were dissolved in MeOH (37 mL/mmol), and a catalytic amount of Pd/C (30% wt/wt) was added. The reaction was left to stir under an atmosphere of hydrogen until no starting material remained by LC-MS analysis. The reaction was then filtered through poly(tetrafluoroethylene) (PTFE) syringe filters, and the filtrate was concentrated *in vacuo* to leave crude amines as colorless oils. Crude amines were dissolved in DMF (6.8 mL/mmol) and added to a solution containing (+)-JQ1 carboxylic acid (1 equiv), DIPEA (2 equiv), and HATU (1.5 equiv) in DMF (27 mL/mmol). This was left to stir at r.t. for 16 h. Reactions were purified without workup by HPLC using a linear gradient from 25 to 95% MeCN in 0.1% formic acid in water over 10 min to afford amide PROTACs, ARV-771 (**22**), **23**, or **24**.

#### General Solution Phase Procedure B

(+)-JQ1 Carboxylic acid (1 equiv) was dissolved in anhydrous DCM (9.4 mL/mmol) under an atmosphere of N_2_. Neat SOCl_2_ (15 equiv) was then added and left to stir at r.t. Conversion to the acid chloride was monitored by LC-MS by dissolving a sample in MeOH and observing the mass of the methyl ester of JQ1 (calcd for C_20_H_20_ClN_4_O_2_S [M + H]^+^ 415.9). Complete conversion was observed after 1.5 h, and the mixture was concentrated *in vacuo*. The residue (1.2 equiv) was redissolved in anhydrous DCM (9.6 mL/mmol), added to N_2_-purged flasks containing alcohols, **51**–**54** (1 equiv), and left to stir at r.t. for 16 h. The mixtures were then concentrated *in vacuo*, and the residues were purified with HPLC using a linear gradient from 25 to 95% MeCN in 0.1% formic acid in water over 10 min to afford ester PROTACs, **25**–**28**.

#### General Solution Phase Procedure C

Diols 3,7-dioxa-1,9-nonanediol or **33** (1 equiv) were dissolved in anhydrous THF (2.6 mL/mmol) under an atmosphere of N_2_. Imidazole (1 equiv) was added as a solid, and the flask was flushed with N_2_. TBDPSCl (1 equiv) was then added dropwise, and the reaction was left to stir at r.t. for 16 h. Et_2_O (20 mL) and water (20 mL) were then added, and the organic layer was separated. The aqueous layer was then extracted with Et_2_O (3 × 20 mL), and the combined organic layers were dried with MgSO_4_, filtered, and concentrated *in vacuo*. The residue was purified by flash column chromatography (24 g silica column) using a linear gradient from 20 to 100% EtOAc in heptane to afford **35** or **36** as colorless oils.

#### General Solution Phase Procedure D

Diols 3,7-dioxa-1,9-nonanediol or **33** (1 equiv) were dissolved in anhydrous THF (2.5 mL/mmol) and cooled to 0 °C. TEA (1 equiv) was then added followed by the addition of *p*-TsCl (1 equiv). The reaction was then heated to 70 °C and stirred for 16 h. Thin-layer chromatography (TLC) confirmed that the *p*-TsCl had been consumed. The mixture was cooled and concentrated *in vacuo* and left under vacuum for 2 h. The residue was then dissolved in EtOH (2.5 mL/mmol) followed by the addition of NaN_3_ (1.5 equiv). This was then carefully refluxed for 24 h. The mixture was concentrated *in vacuo* and redissolved in methyl *tert*-butyl ether (MTBE) and brine. The aqueous phase was separated and washed with EtOAc (3 × 15 mL). The combined MTBE and EtOAc organic layers were dried with MgSO_4_, filtered, and concentrated *in vacuo*. The residue was purified by flash column chromatography (24 g silica column) using a linear gradient from 0 to 10% MeOH in DCM to afford **37** or **38** as colorless/pale yellow oils.

#### General Solution Phase Procedure E

Alcohols **34**–**38** (1 equiv) were dissolved in MeCN (10 mL/mmol) and water (1 mL/mmol). DAIB (2.2 equiv) and TEMPO (0.22 equiv) were then added, and the mixture was left to stir at r.t. for 24 h. The mixture was then concentrated *in vacuo*. Reactions starting with compounds **34**, **37**, or **38** were then basified with 2 M NaOH solution and washed with MTBE. The aqueous layer was subsequently acidified with 2 M HCl and extracted with DCM (×3). The DCM layers were dried with MgSO_4_, filtered, and concentrated *in vacuo* to afford crude carboxylic acids **39**, **42**, or **43** as colorless oils without the need for purification. Reactions starting with compounds **35** or **36** were purified without workup by reverse phase flash column chromatography (50 g C18 gold column) using a linear gradient from 40% to 100% MeCN in 0.1% formic acid in water to afford carboxylic acids, **40** or **41**, as colorless oils.

#### General Solution Phase Procedure F

To solutions of carboxylic acids **39**–**43** (1.2 equiv) in DMF (1 mL/mmol), DIPEA (4 equiv) was added followed by HATU (1.5 equiv) and left to stir at r.t. for 10 min. VHL-NH_3_Cl or ME-VHL-NH_3_Cl (1 equiv) was dissolved in DMF (4.6 mL/mmol) and then added to the flasks containing **44** and **47**–**50** or **45** and **46**, respectively, and left to stir at r.t. for 1–2 h (LC-MS control). Reactions starting with compounds **39**–**41** were purified without workup by reverse phase flash column chromatography (15.5 g C18 gold column) using a linear gradient from 40 to 100% MeCN in 0.1% formic acid in water to afford TBDPS-protected alcohols, **44**, **46**, **48**, or **50**, as colorless oils. Reactions starting with compounds **42** or **43** were purified without workup by reverse phase flash column chromatography (15.5 g C18 gold column) using a linear gradient from 5 to 100% MeCN in 0.1% formic acid in water to afford azides, **45**, **47**, or **49**, as colorless oils.

#### General Solution Phase Procedure G

Compounds **44**, **46**, **48**, or **50** were dissolved in THF (166 mL/mmol). A solution of 1.0 M tetra-*n*-butylammonium fluoride (TBAF) in THF (3 equiv) was added, and the reaction was left to stir at r.t. for 16 h. Full conversion to the free alcohol was observed by LC-MS. The mixtures were concentrated *in vacuo*. The reaction with **44** was purified directly as described below. The residues from **46**, **48**, and **50** were redissolved in Et_2_O (5 mL), and saturated NH_4_Cl (10 mL) solution was added; this was stirred for 10 min. The aqueous layer was then extracted with Et_2_O (4 × 10 mL), and the combined organic layers were dried with Na_2_SO_4_, filtered, and concentrated *in vacuo*. The residues were purified by reverse phase flash column chromatography (15.5 g C18 gold column) using a linear gradient from 5 to 100% MeCN in 0.1% formic acid in water to afford alcohols **51**–**54** as colorless oils.

#### (2*S*,4*R*)-4-Hydroxy-1-((*S*)-2-(6-(2-methoxyacetamido)hexanamido)-3,3-dimethylbutanoyl)-*N*-(4-(4-methylthiazol-5-yl)benzyl)pyrrolidine-2-carboxamide (VK-P01) (**1**)

Yield: 3.5 mg (26%); ^1^H NMR (CDCl_3_, 500 MHz) δ 8.82 (s, 1H), 7.43–7.34 (m, 4H), 6.59 (s, 1H), 6.15 (d, *J* = 8.7 Hz, 1H), 4.78 (td, *J* = 8.0, 4.3 Hz, 1H), 4.61 (dt, *J* = 14.9, 6.3 Hz, 1H), 4.52 (dd, *J* = 18.6, 8.7 Hz, 1H), 4.41–4.33 (m, 1H), 4.15 (d, *J* = 11.2 Hz, 1H), 3.98–3.83 (m, 2H), 3.63 (ddd, *J* = 11.2, 7.3, 3.6 Hz, 1H), 3.43 (d, *J* = 9.1 Hz, 3H), 3.29 (q, *J* = 6.8 Hz, 1H), 2.69–2.56 (m, 1H), 2.56 (s, 3H), 2.23 (dtt, *J* = 22.0, 14.4, 7.5 Hz, 2H), 1.66 (ddq, *J* = 28.3, 13.9, 7.1 Hz, 1H), 1.52 (p, *J* = 7.6, 7.2 Hz, 1H), 1.34 (s, 1H), 1.38–1.28 (m, 1H), 1.28 (s, 1H), 0.97 (d, *J* = 6.5 Hz, 8H). LC-MS *m*/*z* calcd for C_31_H_45_N_5_O_6_S [M + H]^+^: 616.3169, found: 616.93.

#### (2*S*,4*R*)-1-((*S*)-2-(6-Butyramidohexanamido)-3,3-dimethylbutanoyl)-4-hydroxy-*N*-(4-(4-methylthiazol-5-yl)benzyl)pyrrolidine-2-carboxamide (VK-P02) (**2**)

Yield: 3.6 mg (27%); ^1^H NMR (CDCl_3_, 500 MHz) δ 8.81 (s, 1H), 7.37 (s, 3H), 7.36 (d, *J* = 6.6 Hz, 2H), 6.14 (d, *J* = 8.8 Hz, 1H), 5.60 (s, 1H), 4.79–4.71 (m, 1H), 4.59 (ddd, *J* = 15.0, 6.7, 2.7 Hz, 1H), 4.54 (s, 1H), 4.50 (dd, *J* = 16.9, 8.7 Hz, 1H), 4.35 (ddd, *J* = 14.8, 9.6, 5.2 Hz, 1H), 4.15 (t, *J* = 12.1 Hz, 1H), 3.63–3.56 (m, 1H), 3.22 (dt, *J* = 7.9, 6.3 Hz, 1H), 2.75 (s, 7H), 2.61–2.51 (m, 1H), 2.54 (s, 3H), 2.29–2.09 (m, 4H), 2.16 (s, 1H), 1.72–1.54 (m, 3H), 1.47 (p, *J* = 7.1 Hz, 2H), 1.34–1.24 (m, 2H), 0.96–0.89 (m, 12H). LC-MS *m*/*z* calcd for C_32_H_47_N_5_O_5_S [M + H]^+^: 614.3376, found: 615.01.

#### (2*S*,4*R*)-1-((*S*)-3,3-Dimethyl-2-(6-(2-phenylacetamido)hexanamido)butanoyl)-4-hydroxy-*N*-(4-(4-methylthiazol-5-yl)benzyl)pyrrolidine-2-carboxamide (VK-P03) (**3**)

Yield: 4.4 mg (30%); ^1^H NMR (CDCl_3_, 500 MHz) δ 8.85 (s, 1H), 7.39–7.27 (m, 8H), 7.27–7.21 (m, 2H), 6.17 (d, *J* = 8.7 Hz, 1H), 5.52 (s, 1H), 4.73 (t, *J* = 8.1 Hz, 1H), 4.58 (dd, *J* = 15.0, 6.6 Hz, 1H), 4.53–4.49 (m, 2H), 4.34 (dd, *J* = 15.0, 5.3 Hz, 1H), 4.13–4.07 (m, 1H), 3.58 (dd, *J* = 11.4, 3.3 Hz, 1H), 3.54 (s, 2H), 3.24 (s, 8H), 3.17 (q, *J* = 6.8 Hz, 2H), 2.53 (s, 3H), 2.56–2.47 (m, 1H), 2.22 (dt, *J* = 14.3, 7.1 Hz, 1H), 2.17 (d, *J* = 7.5 Hz, 1H), 2.17–2.10 (m, 1H), 1.68–1.48 (m, 2H), 1.44–1.34 (m, 2H), 1.27–1.17 (m, 3H), 0.94 (s, 9H). LC-MS *m*/*z* calcd for C_36_H_47_N_5_O_5_S [M + H]^+^: 662.3376, found: 662.99.

#### (2*S*,4*R*)-1-((*S*)-2-(6-(Cyclohexanecarboxamido)hexanamido)-3,3-dimethylbutanoyl)-4-hydroxy-*N*-(4-(4-methylthiazol-5-yl)benzyl)pyrrolidine-2-carboxamide (VK-P04) (**4**)

Yield: 4.2 mg (29%); ^1^H NMR (CDCl_3_, 500 MHz) δ 8.83 (s, 1H), 7.39 (s, 1H), 7.37 (s, 4H), 6.20 (d, *J* = 8.7 Hz, 1H), 5.57 (s, 1H), 4.76 (t, *J* = 8.1 Hz, 1H), 4.59 (dd, *J* = 15.0, 6.6 Hz, 1H), 4.53 (t, *J* = 7.0 Hz, 2H), 4.37 (dd, *J* = 15.0, 5.3 Hz, 1H), 4.14 (d, *J* = 11.5 Hz, 1H), 3.60 (dd, *J* = 11.4, 3.4 Hz, 1H), 3.28–3.14 (m, *J* = 6.5 Hz, 2H), 3.05 (s, 8H), 2.58–2.49 (m, 1H), 2.53 (s, 3H), 2.29–2.14 (m, 3H), 2.04 (tt, *J* = 11.8, 3.4 Hz, 1H), 1.85–1.73 (m, 5H), 1.66 (dd, *J* = 13.7, 7.6 Hz, 2H), 1.59 (td, *J* = 14.4, 7.2 Hz, 1H), 1.49–1.42 (m, 2H), 1.44–1.29 (m, 2H), 1.31–1.21 (m, 4H), 1.24–1.14 (m, 2H), 0.94 (s, 9H). LC-MS *m*/*z* calcd for C_35_H_51_N_5_O_5_S [M + H]^+^: 654.3689, found: 665.04.

#### (2*S*,4*R*)-1-((*S*)-2-(6-(2-(Adamantan-1-yl)acetamido)hexanamido)-3,3-dimethylbutanoyl)-4-hydroxy-*N*-(4-(4-methylthiazol-5-yl)benzyl)pyrrolidine-2-carboxamide (VK-P05) (**5**)

Yield: 4.5 mg (28%); ^1^H NMR (CDCl_3_, 500 MHz) δ 8.92 (s, 1H), 7.43–7.34 (m, 5H), 6.28 (d, *J* = 8.7 Hz, 1H), 5.56 (s, 1H), 4.76 (t, *J* = 8.1 Hz, 1H), 4.61 (dd, *J* = 15.1, 6.6 Hz, 1H), 4.54 (d, *J* = 8.9 Hz, 2H), 4.37 (dd, *J* = 15.1, 5.2 Hz, 1H), 4.15 (d, *J* = 11.4 Hz, 1H), 3.76 (s, 3H), 3.68 (s, 6H), 3.62 (dd, *J* = 11.3, 3.4 Hz, 1H), 3.29–3.14 (m, *J* = 7.0 Hz, 2H), 2.54 (s, 3H), 2.57–2.47 (m, 1H), 2.31–2.21 (m, 1H), 2.21 (s, 1H), 2.21–2.15 (m, 1H), 1.95 (s, 3H), 1.91 (s, 2H), 1.70 (s, 1H), 1.66 (d, *J* = 11.7 Hz, 3H), 1.62 (dd, *J* = 13.4, 10.1 Hz, 5H), 1.46 (p, *J* = 7.1 Hz, 2H), 1.30 (s, 2H), 1.33–1.24 (m, 2H), 0.95 (s, 8H), 0.85 (s, 1H). LC-MS *m*/*z* calcd for C_40_H_57_N_5_O_5_ [M + H]^+^: 720.4158, found: 721.15.

#### (2*S*,4*R*)-1-((*S*)-2-(6-(3,3-Diphenylpropanamido)hexanamido)-3,3-dimethylbutanoyl)-4-hydroxy-*N*-(4-(4-methylthiazol-5-yl)benzyl)pyrrolidine-2-carboxamide (VK-P06) (**6**)

Yield: 4.2 mg (25%); ^1^H NMR (CDCl_3_, 500 MHz) δ 8.89 (s, 1H), 7.37 (d, *J* = 1.9 Hz, 4H), 7.29 (d, *J* = 8.5 Hz, 1H), 7.30–7.20 (m, 5H), 7.22–7.14 (m, 4H), 6.16 (d, *J* = 8.7 Hz, 1H), 5.46 (s, 1H), 4.73 (t, *J* = 8.1 Hz, 1H), 4.59 (dd, *J* = 15.1, 6.7 Hz, 1H), 4.55–4.49 (m, 3H), 4.35 (dd, *J* = 15.1, 5.2 Hz, 1H), 4.12 (d, *J* = 11.4 Hz, 1H), 3.59 (dd, *J* = 11.4, 3.4 Hz, 1H), 3.06 (hept, *J* = 6.7 Hz, 2H), 2.87 (d, *J* = 7.9 Hz, 2H), 2.54 (s, 4H), 2.56–2.48 (m, 1H), 2.13 (ddt, *J* = 28.9, 14.6, 7.4 Hz, 3H), 1.50 (ddt, *J* = 41.5, 13.9, 7.2 Hz, 2H), 1.27–1.17 (m, 4H), 1.10–1.01 (m, 3H), 0.95 (s, 9H). LC-MS *m*/*z* calcd for C_43_H_53_N_5_O_5_S [M + H]^+^: 752.3845, found: 753.18.

#### (2*S*,4*R*)-1-((*S*)-2-(6-(4′-Butoxy-[1,1′-biphenyl]-4-carboxamido)hexanamido)-3,3-dimethylbutanoyl)-4-hydroxy-*N*-(4-(4-methylthiazol-5-yl)benzyl)pyrrolidine-2-carboxamide (VK-P07) (**7**)

Yield: 2.5 mg (14%); ^1^H NMR (CDCl_3_, 500 MHz) δ 8.81 (s, 1H), 7.79 (d, *J* = 8.2 Hz, 2H), 7.63–7.49 (m, 5H), 7.35 (s, 4H), 6.97 (d, *J* = 8.7 Hz, 2H), 6.33 (d, *J* = 6.0 Hz, 1H), 6.14 (d, *J* = 8.7 Hz, 1H), 4.73 (t, *J* = 8.1 Hz, 1H), 4.56 (dd, *J* = 15.0, 6.6 Hz, 1H), 4.51 (d, *J* = 8.6 Hz, 2H), 4.33 (dd, *J* = 15.1, 5.2 Hz, 1H), 4.11 (d, *J* = 11.4 Hz, 1H), 4.01 (t, *J* = 6.5 Hz, 3H), 3.57 (dd, *J* = 11.4, 3.4 Hz, 1H), 3.45 (q, *J* = 6.6 Hz, 2H), 2.54 (s, 3H), 2.51 (dd, *J* = 8.4, 4.7 Hz, 1H), 2.32–2.11 (m, 3H), 1.84–1.75 (m, 1H), 1.74–1.46 (m, 6H), 1.39 (s, 2H), 1.26 (s, 6H), 1.15 (s, 1H), 0.99 (t, *J* = 7.4 Hz, 4H), 0.94 (s, 9H), 0.88 (t, *J* = 6.2 Hz, 2H), 0.84 (s, 3H), 0.76 (s, 1H). LC-MS *m*/*z* calcd for C_45_H_57_N_5_O_6_S [M + H]^+^: 796.4108, found: 797.13.

#### 6-(((*S*)-1-((2*S*,4*R*)-4-Hydroxy-2-((4-(4-methylthiazol-5-yl)benzyl)carbamoyl)pyrrolidin-1-yl)-3,3-dimethyl-1-oxobutan-2-yl)amino)-6-oxohexyl 2-methoxyacetate (VK-P08) (**8**)

Purity by HPLC: 75%; yield: 2.4 mg (18%); ^1^H NMR (CDCl_3_, 500 MHz) δ 8.87 (s, 1H), 7.38 (s, 3H), 7.34–7.26 (m, 1H), 7.20–7.14 (m, 1H), 6.86–6.81 (m, 1H), 6.11 (d, *J* = 8.7 Hz, 1H), 4.72 (t, *J* = 8.0 Hz, 1H), 4.61 (dd, *J* = 15.0, 6.6 Hz, 1H), 4.55 (s, 1H), 4.50 (d, *J* = 8.7 Hz, 1H), 4.36 (dd, *J* = 15.0, 5.2 Hz, 1H), 4.21–4.11 (m, 2H), 4.02 (d, *J* = 0.9 Hz, 1H), 3.80 (s, 2H), 3.61 (dd, *J* = 11.4, 3.5 Hz, 1H), 3.43 (s, 2H), 2.56 (ddd, *J* = 12.7, 7.9, 4.5 Hz, 1H), 2.23 (dd, *J* = 7.4, 4.4 Hz, 1H), 2.23–2.12 (m, 1H), 1.70–1.59 (m, 1H), 1.47–1.30 (m, 1H), 1.35 (s, 1H), 1.27 (d, *J* = 14.3 Hz, 1H), 0.94 (s, 6H). LC-MS *m*/*z* calcd for C_31_H_44_N_4_O_7_S [M + H]^+^: 617.3009, found: 617.29.

#### 6-(((*S*)-1-((2*S*,4*R*)-4-Hydroxy-2-((4-(4-methylthiazol-5-yl)benzyl)carbamoyl)pyrrolidin-1-yl)-3,3-dimethyl-1-oxobutan-2-yl)amino)-6-oxohexyl Butyrate (VK-P09) (**9**)

Yield: 1.9 mg (14%); ^1^H NMR (CDCl_3_, 500 MHz) δ 8.77 (s, 1H), 7.41–7.33 (m, 4H), 6.04 (d, *J* = 8.6 Hz, 1H), 4.74 (t, *J* = 7.9 Hz, 1H), 4.59 (dd, *J* = 14.9, 6.7 Hz, 1H), 4.54 (s, 1H), 4.48 (d, *J* = 8.6 Hz, 1H), 4.34 (dd, *J* = 14.9, 5.1 Hz, 1H), 4.14 (d, *J* = 11.6 Hz, 1H), 4.05 (t, *J* = 6.7 Hz, 2H), 3.59 (dd, *J* = 11.5, 3.5 Hz, 1H), 2.59 (ddd, *J* = 12.8, 7.9, 4.6 Hz, 1H), 2.54 (s, 3H), 2.24 (dt, *J* = 23.0, 7.5 Hz, 4H), 2.01 (s, 3H), 1.63 (tt, *J* = 10.5, 7.2 Hz, 5H), 1.35 (ddt, *J* = 15.1, 7.2, 4.0 Hz, 2H), 1.26 (s, 7H), 1.08–0.98 (m, 1H), 0.97–0.81 (m, 12H), 0.79–0.68 (m, 1H). LC-MS *m*/*z* calcd for C_32_H_46_N_4_O_6_S [M + H]^+^: 615.3216, found: 615.31.

#### 6-(((*S*)-1-((2*S*,4*R*)-4-Hydroxy-2-((4-(4-methylthiazol-5-yl)benzyl)carbamoyl)pyrrolidin-1-yl)-3,3-dimethyl-1-oxobutan-2-yl)amino)-6-oxohexyl 2-phenylacetate (VK-P10) (**10**)

Yield: 1.2 mg (8%); ^1^H NMR (CDCl_3_, 500 MHz) δ 8.81 (s, 1H), 7.40–7.30 (m, 4H), 7.33–7.26 (m, 3H), 6.01 (d, *J* = 8.5 Hz, 1H), 4.72 (t, *J* = 7.9 Hz, 1H), 4.58 (dd, *J* = 15.0, 6.7 Hz, 1H), 4.53 (s, 1H), 4.47 (d, *J* = 8.6 Hz, 1H), 4.34 (dd, *J* = 14.9, 5.1 Hz, 1H), 4.12 (d, *J* = 11.6 Hz, 1H), 4.07 (t, *J* = 6.6 Hz, 2H), 3.60 (s, 2H), 3.61–3.55 (m, 1H), 2.62–2.55 (m, 1H), 2.55 (s, 3H), 2.21–2.09 (m, 2H), 1.60 (p, *J* = 7.1 Hz, 4H), 1.33–1.24 (m, 4H), 0.93 (s, 8H). LC-MS *m*/*z* calcd for C_36_H_46_N_4_O_6_S [M + H]^+^: 663.3216, found: 663.26.

#### 6-(((*S*)-1-((2*S*,4*R*)-4-Hydroxy-2-((4-(4-methylthiazol-5-yl)benzyl)carbamoyl)pyrrolidin-1-yl)-3,3-dimethyl-1-oxobutan-2-yl)amino)-6-oxohexyl cyclohexanecarboxylate (VK-P11) (**11**)

Yield: 1.9 mg (13%); ^1^H NMR (CDCl_3_, 500 MHz) δ 8.85 (s, 1H), 7.38 (s, 4H), 7.28 (s, 1H), 6.07 (d, *J* = 8.6 Hz, 1H), 4.74 (t, *J* = 7.9 Hz, 1H), 4.60 (dd, *J* = 15.0, 6.7 Hz, 1H), 4.55 (s, 1H), 4.49 (d, *J* = 8.6 Hz, 1H), 4.36 (dd, *J* = 14.9, 5.1 Hz, 1H), 4.15 (d, *J* = 11.5 Hz, 1H), 4.03 (td, *J* = 6.6, 1.1 Hz, 2H), 3.60 (dd, *J* = 11.4, 3.5 Hz, 1H), 2.62–2.54 (m, 1H), 2.55 (s, 3H), 2.27 (ddt, *J* = 11.3, 7.7, 3.6 Hz, 1H), 2.22 (t, *J* = 7.5 Hz, 2H), 1.87 (d, *J* = 13.0 Hz, 2H), 1.76–1.70 (m, 2H), 1.62 (dt, *J* = 14.4, 7.5 Hz, 5H), 1.42 (s, 1H), 1.41–1.31 (m, 2H), 1.31–1.27 (m, 1H), 1.26 (s, 2H), 1.24–1.17 (m, 1H), 0.94 (s, 8H). LC-MS *m*/*z* calcd for C_35_H_50_N_4_O_6_S [M + H]^+^: 655.3529, found: 655.20.

#### 6-(((*S*)-1-((2*S*,4*R*)-4-Hydroxy-2-((4-(4-methylthiazol-5-yl)benzyl)carbamoyl)pyrrolidin-1-yl)-3,3-dimethyl-1-oxobutan-2-yl)amino)-6-oxohexyl 2-(adamantan-1-yl)acetate (VK-P12) (**12**)

Yield: 2.2 mg (14%); ^1^H NMR (CDCl_3_, 500 MHz) δ 8.92 (s, 1H), 7.38 (s, 4H), 6.07 (d, *J* = 8.6 Hz, 1H), 4.74 (t, *J* = 8.0 Hz, 1H), 4.60 (dd, *J* = 15.1, 6.7 Hz, 1H), 4.55 (s, 1H), 4.49 (d, *J* = 8.7 Hz, 1H), 4.36 (dd, *J* = 15.1, 5.1 Hz, 1H), 4.15 (d, *J* = 11.5 Hz, 1H), 4.03 (td, *J* = 6.7, 1.2 Hz, 2H), 3.60 (dd, *J* = 11.4, 3.5 Hz, 1H), 2.57 (s, 3H), 2.62–2.53 (m, 1H), 2.25–2.11 (m, 4H), 2.04 (s, 2H), 1.96 (s, 4H), 1.71 (s, 1H), 1.70–1.61 (m, 9H), 1.59 (d, *J* = 2.7 Hz, 7H), 1.36 (qd, *J* = 8.7, 8.2, 5.3 Hz, 2H), 1.26 (s, 4H), 0.94 (s, 9H), 0.88 (t, *J* = 6.9 Hz, 0H). LC-MS *m*/*z* calcd for C_40_H_56_N_4_O_6_S [M + H]^+^: 721.3999, found: 721.39.

#### 6-(((*S*)-1-((2*S*,4*R*)-4-Hydroxy-2-((4-(4-methylthiazol-5-yl)benzyl)carbamoyl)pyrrolidin-1-yl)-3,3-dimethyl-1-oxobutan-2-yl)amino)-6-oxohexyl 3,3-diphenylpropanoate (VK-P13) (**13**)

Yield: 3.0 mg (18%); ^1^H NMR (CDCl_3_, 500 MHz) δ 8.80 (s, 1H), 7.37 (d, *J* = 1.6 Hz, 4H), 7.30–7.14 (m, 11H), 6.02 (d, *J* = 8.6 Hz, 1H), 4.72 (t, *J* = 8.0 Hz, 1H), 4.63–4.45 (m, 5H), 4.34 (dd, *J* = 14.9, 5.2 Hz, 1H), 4.13 (d, *J* = 11.5 Hz, 1H), 3.95 (t, *J* = 6.6 Hz, 2H), 3.59 (dd, *J* = 11.4, 3.5 Hz, 1H), 3.04 (d, *J* = 8.1 Hz, 2H), 2.58 (ddd, *J* = 12.9, 7.8, 4.6 Hz, 1H), 2.54 (s, 3H), 2.17–2.08 (m, 3H), 1.49 (dq, *J* = 38.0, 7.2, 6.8 Hz, 4H), 1.26 (s, 1H), 1.21–1.12 (m, 2H), 0.93 (s, 9H). LC-MS *m*/*z* calcd for C_43_H_52_N_4_O_6_S [M + H]^+^: 753.3686, found: 753.24.

#### 6-(((*S*)-1-((2*S*,4*R*)-4-Hydroxy-2-((4-(4-methylthiazol-5-yl)benzyl)carbamoyl)pyrrolidin-1-yl)-3,3-dimethyl-1-oxobutan-2-yl)amino)-6-oxohexyl 4′-butoxy-[1,1′-biphenyl]-4-carboxylate (VK-P14) (**14**)

Purity by HPLC ∼ 43%; yield: 1.3 mg (7%). ^1^H NMR (CDCl_3_, 500 MHz) δ 8.88 (s, 1H), 8.13–8.05 (m, 1H), 8.04 (d, *J* = 8.5 Hz, 2H), 7.67–7.50 (m, 7H), 7.37 (s, 4H), 7.02–6.96 (m, 4H), 6.09 (d, *J* = 8.5 Hz, 1H), 4.73 (t, *J* = 8.0 Hz, 1H), 4.59 (dd, *J* = 15.1, 6.6 Hz, 1H), 4.54 (s, 1H), 4.49 (d, *J* = 8.6 Hz, 1H), 4.38–4.29 (m, 3H), 4.15 (d, *J* = 11.3 Hz, 1H), 4.02 (t, *J* = 6.5 Hz, 4H), 3.60 (dd, *J* = 11.4, 3.5 Hz, 1H), 3.14 (s, 1H), 2.96 (s, 1H), 2.56 (s, 3H), 2.36 (t, *J* = 7.6 Hz, 1H), 2.26 (t, *J* = 7.4 Hz, 3H), 2.18–2.10 (m, 1H), 1.81 (q, *J* = 7.2 Hz, 5H), 1.53 (dt, *J* = 15.1, 7.4 Hz, 4H), 1.48 (s, 2H), 1.45 (d, *J* = 6.7 Hz, 1H), 1.26 (s, 11H), 1.06–0.96 (m, 10H), 0.93 (s, 9H), 0.88 (t, *J* = 6.9 Hz, 1H). LC-MS *m*/*z* calcd for C_45_H_56_N_4_O_7_S [M + H]^+^: 797.3948, found: 797.26.

#### (2*S*,4*R*)-1-((*S*)-3,3-Dimethyl-2-(3-(2-(2-phenylacetamido)ethoxy)propanamido)butanoyl)-4-hydroxy-N-(4-(4-methylthiazol-5-yl)benzyl)pyrrolidine-2-carboxamide (VK-P15) (**15**)

Purity by HPLC ∼ 74%; yield: 1.1 mg (8%); ^1^H NMR (CDCl_3_, 500 MHz) δ 8.80 (s, 1H), 7.40–7.26 (m, 7H), 6.98 (d, *J* = 8.9 Hz, 1H), 6.88 (t, *J* = 5.9 Hz, 1H), 6.75 (s, 1H), 4.68 (t, *J* = 8.0 Hz, 1H), 4.53 (d, *J* = 8.8 Hz, 1H), 4.41 (dd, *J* = 15.0, 6.5 Hz, 1H), 4.27 (dd, *J* = 15.0, 5.5 Hz, 1H), 4.08 (d, *J* = 11.4 Hz, 1H), 3.67–3.55 (m, 4H), 3.58–3.42 (m, 3H), 3.39–3.32 (m, 1H), 2.53 (s, 3H), 2.52–2.36 (m, 2H), 2.18–2.10 (m, 0H), 1.54 (t, *J* = 7.0 Hz, 1H), 1.45 (d, *J* = 6.6 Hz, 1H), 1.26 (s, 2H), 0.96 (s, 9H). LC-MS *m*/*z* calcd for C_35_H_45_N_5_O_6_S [M + H]^+^: 664.3169, found: 664.22.

#### (2*S*,4*R*)-1-((*S*)-14-(*tert*-Butyl)-2,12-dioxo-1-phenyl-6,9-dioxa-3,13-diazapentadecan-15-oyl)-4-hydroxy-N-(4-(4-methylthiazol-5-yl)benzyl)pyrrolidine-2-carboxamide (VK-P16) (**16**)

Yield: 1.0 mg (6%); ^1^H NMR (CDCl_3_, 500 MHz) δ 10.90 (s, 1H), 8.77 (s, 1H), 8.07 (s, 1H), 7.78 (dd, *J* = 7.5, 1.0 Hz, 1H), 7.65–7.59 (m, 1H), 7.44–7.34 (m, 1H), 7.37–7.29 (m, 6H), 7.30 (d, *J* = 0.9 Hz, 1H), 7.27 (s, 2H), 7.00 (d, *J* = 8.3 Hz, 1H), 6.33 (s, 1H), 4.67 (t, *J* = 8.1 Hz, 1H), 4.57 (dd, *J* = 15.0, 6.7 Hz, 1H), 4.52–4.46 (m, 2H), 4.32 (dd, *J* = 15.0, 5.2 Hz, 1H), 4.09–4.02 (m, 2H), 3.76–3.61 (m, 3H), 3.64–3.46 (m, 9H), 3.37 (ddt, *J* = 14.0, 8.8, 4.0 Hz, 2H), 3.11 (qd, *J* = 7.4, 4.2 Hz, 2H), 2.52 (s, 3H), 2.46 (h, *J* = 4.1 Hz, 3H), 2.19–2.10 (m, 1H), 1.50–1.34 (m, 13H), 1.26 (s, 3H), 0.95 (s, 9H). HRMS *m*/*z* calcd for C_37_H_49_N_5_O_7_S [M + H]^+^: 708.3431, found: 708.3423.

#### (2*S*,4*R*)-1-((*S*)-17-(*tert*-Butyl)-2,15-dioxo-1-phenyl-6,9,12-trioxa-3,16-diazaoctadecan-18-oyl)-4-hydroxy-*N*-(4-(4-methylthiazol-5-yl)benzyl)pyrrolidine-2-carboxamide (VK-P17) (**17**)

Yield: 1.9 mg (12%); ^1^H NMR (CDCl_3_, 500 MHz) δ 8.88 (s, 2H), 7.36 (s, 8H), 7.35–7.25 (m, 7H), 6.45 (s, 1H), 4.70 (t, *J* = 8.2 Hz, 2H), 4.58 (dd, *J* = 15.1, 6.5 Hz, 2H), 4.48 (d, *J* = 8.9 Hz, 3H), 4.35 (dd, *J* = 15.1, 5.2 Hz, 2H), 4.13 (d, *J* = 11.4 Hz, 2H), 3.75 (s, 2H), 3.68 (d, *J* = 4.8 Hz, 1H), 3.63–3.56 (m, 12H), 3.55 (s, 6H), 3.50 (d, *J* = 4.9 Hz, 2H), 3.41 (s, 4H), 2.98 (d, *J* = 2.5 Hz, 1H), 2.90 (s, 1H), 2.67 (s, 11H), 2.53 (s, 6H), 2.35 (t, *J* = 7.5 Hz, 1H), 2.17 (t, *J* = 11.0 Hz, 3H), 2.01 (s, 7H), 1.67–1.61 (m, 1H), 1.46–1.38 (m, 1H), 1.26 (s, 12H), 1.04 (d, *J* = 12.6 Hz, 1H), 0.96 (s, 1H), 0.95 (s, 17H), 0.94–0.83 (m, 3H). HRMS *m*/*z* calcd for C_39_H_53_N_5_O_8_S [M + H]^+^: 752.3693, found: 752.3681. Yield: 1.9 mg (12%).

#### 2-(3-(((*S*)-1-((2*S*,4*R*)-4-Hydroxy-2-((4-(4-methylthiazol-5-yl)benzyl)carbamoyl)pyrrolidin-1-yl)-3,3-dimethyl-1-oxobutan-2-yl)amino)-3-oxopropoxy)ethyl 2-phenylacetate (VK-P18) (**18**)

Yield: 1.2 mg (8%); ^1^H NMR (CDCl_3_, 500 MHz) δ 8.91 (s, 1H), 7.40–7.34 (m, 5H), 7.34–7.26 (m, 5H), 6.85 (d, *J* = 8.2 Hz, 1H), 4.73 (t, *J* = 7.9 Hz, 2H), 4.56 (dd, *J* = 15.0, 6.7 Hz, 2H), 4.51 (s, 2H), 4.44 (d, *J* = 8.2 Hz, 1H), 4.33 (dd, *J* = 15.1, 5.3 Hz, 1H), 4.28–4.20 (m, 3H), 4.12 (d, *J* = 11.5 Hz, 2H), 3.75–3.63 (m, 11H), 3.61–3.52 (m, 2H), 3.13 (d, *J* = 10.7 Hz, 1H), 2.95 (d, *J* = 5.6 Hz, 1H), 2.63–2.52 (m, 7H), 2.50–2.41 (m, 3H), 2.36 (q, *J* = 8.5, 7.5 Hz, 1H), 2.12 (dd, *J* = 14.0, 7.3 Hz, 2H), 2.01 (s, 5H), 1.26 (s, 29H), 1.10 (s, 1H), 1.05 (s, 3H), 1.06–0.96 (m, 1H), 0.94 (s, 11H), 0.89 (d, *J* = 6.8 Hz, 2H), 0.89–0.81 (m, 2H), 0.81–0.68 (m, 4H). LC-MS *m*/*z* calcd for C_35_H_44_N_4_O_7_S [M + H]^+^: 665.3009, found: 665.24.

#### 2-(2-(3-(((*S*)-1-((2*S*,4*R*)-4-Hydroxy-2-((4-(4-methylthiazol-5-yl)benzyl)carbamoyl)pyrrolidin-1-yl)-3,3-dimethyl-1-oxobutan-2-yl)amino)-3-oxopropoxy)ethoxy)ethyl 2-phenylacetate (VK-P19) (**19**)

Purity by HPLC ∼ 57%. Yield: 1.0 mg (6%); ^1^H NMR (CDCl_3_, 500 MHz) δ 8.90 (s, 1H), 7.36 (s, 3H), 7.35–7.26 (m, 4H), 7.07 (d, *J* = 7.9 Hz, 1H), 4.73 (t, *J* = 8.0 Hz, 1H), 4.58 (dd, *J* = 15.1, 6.7 Hz, 1H), 4.52 (s, 1H), 4.43 (d, *J* = 8.0 Hz, 1H), 4.37–4.18 (m, 3H), 4.15 (d, *J* = 11.5 Hz, 1H), 3.70 (t, *J* = 5.6 Hz, 2H), 3.69–3.62 (m, 4H), 3.60 (s, 3H), 3.60–3.55 (m, 1H), 2.56 (dd, *J* = 13.6, 5.0 Hz, 3H), 2.56–2.46 (m, 2H), 2.13 (dd, *J* = 13.6, 8.0 Hz, 1H), 1.26 (s, 2H), 0.94 (s, 9H), 0.85 (s, 1H). LC-MS *m*/*z* calcd for C_37_H_48_N_4_O_8_S [M + H]^+^: 709.3271, found: 709.22.

#### (*S*)-14-((2*S*,4*R*)-4-Hydroxy-2-((4-(4-methylthiazol-5-yl)benzyl)carbamoyl)pyrrolidine-1-carbonyl)-15,15-dimethyl-12-oxo-3,6,9-trioxa-13-azahexadecyl 2-phenylacetate (VK-P20) (**20**)

Purity by HPLC ∼ 61%. Yield: 0.9 mg (5%); ^1^H NMR (CDCl_3_, 500 MHz) δ 8.77 (s, 1H), 7.39–7.25 (m, 9H), 7.00 (d, *J* = 7.9 Hz, 1H), 4.74 (t, *J* = 8.0 Hz, 1H), 4.57 (dd, *J* = 14.9, 6.7 Hz, 1H), 4.51 (s, 1H), 4.42 (d, *J* = 7.9 Hz, 1H), 4.33 (dd, *J* = 15.0, 5.2 Hz, 1H), 4.25 (td, *J* = 4.4, 1.5 Hz, 2H), 4.17 (d, *J* = 11.4 Hz, 1H), 3.71 (dd, *J* = 5.5, 2.5 Hz, 1H), 3.72–3.63 (m, 2H), 3.63 (d, *J* = 7.5 Hz, 6H), 3.58 (s, 3H), 3.61–3.52 (m, 1H), 2.51 (d, *J* = 17.0 Hz, 6H), 2.14 (dd, *J* = 13.6, 8.2 Hz, 1H), 1.26 (s, 2H), 0.94 (s, 9H), 0.89 (d, *J* = 12.6 Hz, 1H). LC-MS *m*/*z* calcd for C_39_H_52_N_4_O_9_S [M + H]^+^: 753.3533, found: 753.31.

#### (2*S*,4*R*)-1-((*S*)-2-(*tert*-Butyl)-17-((*S*)-4-(4-chlorophenyl)-2,3,9-trimethyl-6*H*-thieno[3,2-*f*][1,2,4]triazolo[4,3-*a*][1,4]diazepin-6-yl)-4,16-dioxo-6,12-dioxa-3,15-diazaheptadecanoyl)-4-hydroxy-*N*-(4-(4-methylthiazol-5-yl)benzyl)pyrrolidine-2-carboxamide (AB1) (**23**)

Follow General Solution Phase Procedure A using azide **47**. Yield: 9.1 mg (62%); ^1^H NMR (CDCl_3_, 400 MHz): δ, ppm 8.76 (1H, s), 7.47–7.41 (3H, m), 7.36–7.31 (6H, m), 7.23 (1H, d, *J* = 8.9 Hz), 7.07 (1H, t, *J* = 5.1 Hz), 4.76–4.69 (2H, m), 4.56–4.48 (3H, m), 4.35 (1H, dd, *J* = 5.2, 15.2 Hz), 4.07 (1H, d, *J* = 11.6 Hz), 3.96 (1H, d, *J* = 15.4 Hz), 3.90 (1H, d, *J* = 15.5 Hz), 3.65 (1H, dd, *J* = 3.6, 11.1 Hz), 3.55–3.34 (10H, m), 2.68 (3H, s), 2.51 (3H, s), 2.46–2.38 (4H, m), 2.13 (1H, dd, *J* = 8.1, 13.3 Hz), 1.68–1.56 (7H, m), 1.52–1.41 (2H, m), 0.98 (9H, s); ^13^C NMR (CDCl_3_, 126 MHz): δ, ppm 171.4, 171.1, 170.7, 170.6, 164.3, 155.7, 150.5, 150.2, 148.5, 138.4, 137.1, 136.5, 132.3, 131.9, 131.23, 131.21, 130.9, 130.6, 130.1, 129.6, 128.9, 128.2, 71.8, 71.0, 70.3, 70.1, 69.3, 58.7, 57.1, 56.9, 54.3, 43.3, 41.2, 39.7, 38.7, 36.4, 35.3, 29.3, 26.6, 22.9, 16.1, 14.5, 13.2, 11.9; HRMS *m*/*z* calcd for C_50_H_63_ClN_9_O_7_S_2_ [M + H]^+^ 1000.3975, found: 1000.4962.

#### (2*S*,4*R*)-1-((*S*)-2-(*tert*-Butyl)-15-((*S*)-4-(4-chlorophenyl)-2,3,9-trimethyl-6*H*-thieno[3,2-*f*][1,2,4]triazolo[4,3-*a*][1,4]diazepin-6-yl)-4,14-dioxo-6,10-dioxa-3,13-diazapentadecanoyl)-4-hydroxy-*N*-(4-(4-methylthiazol-5-yl)benzyl)pyrrolidine-2-carboxamide (AB2) (**24**)

Follow General Solution Phase Procedure A using azide **49**. Yield: 6.5 mg (55%); ^1^H NMR (CDCl_3_, 500 MHz): δ, ppm 8.67 (1H, s), 7.60–7.53 (2H, m), 7.35 (2H, d, *J* = 8.2 Hz), 7.32 (1H, d, *J* = 9.0 Hz), 7.30–7.23 (6H, m), 4.81 (1H, t, *J* = 8.2 Hz), 4.66–4.61 (2H, m), 4.53 (1H, br. s), 4.45 (1H, dd, *J* = 6.4, 15.4 Hz), 4.15 (1H, d, *J* = 11.2 Hz), 4.08 (1H, dd, *J* = 5.5, 15.3 Hz), 4.02 (1H, d, *J* = 15.7 Hz), 3.93 (1H, d, *J* = 15.6 Hz), 3.71–3.50 (8H, m), 3.47 (1H, dd, *J* = 8.0, 15.0 Hz), 3.38 (1H, dd, *J* = 5.8, 14.9 Hz), 3.34-3.27 (1H, m), 2.63 (3H, s), 2.50 (3H, s), 2.39 (3H, s), 2.36–2.28 (1H, m), 2.18 (1H, dd, *J* = 7.9, 13.5 Hz), 1.96–1.86 (1H, m), 1.85–1.77 (1H, m), 1.64 (3H, s), 1.02 (9H, s); ^13^C NMR (CDCl_3_, 126 MHz): δ, ppm 171.6, 171.4, 170.8, 170.7, 164.4, 155.7, 150.4, 150.2, 148.5, 138.6, 137.2, 136.5, 132.3, 131.9, 131.4, 131.3, 130.6, 130.1, 129.4, 128.9, 127.9, 70.5, 70.4, 69.3, 67.9, 59.1, 57.3, 57.2, 54.3, 43.0, 39.8, 38.0, 37.2, 35.5, 29.3, 26.6, 16.2, 14.5, 13.3, 11.9; HRMS *m*/*z* calcd for C_48_H_59_ClN_9_O_7_S_2_ [M + H]^+^ 972.3662, found: 972.4628.

#### (*S*)-13-((2*S*,4*R*)-4-Hydroxy-2-((4-(4-methylthiazol-5-yl)benzyl)carbamoyl)pyrrolidine-1-carbonyl)-14,14-dimethyl-11-oxo-3,6,9-trioxa-12-azapentadecyl 2-((*S*)-4-(4-Chlorophenyl)-2,3,9-trimethyl-6*H*-thieno[3,2-*f*][1,2,4]triazolo[4,3-*a*][1,4]diazepin-6-yl)acetate (OMZ1) (**25**)

Follow General Solution Phase Procedure B using alcohol **51**. Yield: 5.4 mg (20%); ^1^H NMR (CDCl_3_, 400 MHz): δ, ppm 8.67 (1H, s), 7.44–7.27 (10H, m), 4.77 (1H, t, *J* = 7.8 Hz), 4.62–4.49 (4H, m), 4.39–4.23 (3H, m), 4.10 (1H, d, *J* = 10.7 Hz), 4.03 (1H, d, *J* = 15.4 Hz), 3.98 (1H, d, *J* = 15.6 Hz), 3.75–3.54 (13H, m), 2.66 (3H, s), 2.58–2.49 (4H, m), 2.41 (3H, s), 2.18 (1H, dd, *J* = 9.0, 12.9 Hz), 1.69 (3H, s), 0.96 (9H, s); ^13^C NMR (CDCl_3_, 126 MHz): δ, ppm 171.7, 171.5, 171.0, 170.3, 164.0, 155.4, 150.4, 150.0, 148.6, 138.4, 137.0, 136.7, 132.3, 131.8, 131.03, 130.99, 130.5, 130.0, 129.6, 128.8, 128.3, 71.3, 70.9, 70.8, 70.7, 70.6, 70.3, 69.1, 64.2, 58.6, 57.2, 56.9, 53.9, 43.4, 36.9, 36.2, 35.3, 35.2, 26.6, 16.2, 14.6, 13.3, 11.9; HRMS *m*/*z* calcd for C_49_H_60_ClN_8_O_9_S_2_ [M + H]^+^ 1003.3608, found: 1003.3437.

#### 2-(3-(2-(((*S*)-1-((2*S*,4*R*)-4-Hydroxy-2-(((*S*)-1-(4-(4-methylthiazol-5-yl)phenyl)ethyl)carbamoyl)pyrrolidin-1-yl)-3,3-dimethyl-1-oxobutan-2-yl)amino)-2-oxoethoxy)propoxy)ethyl 2-((*S*)-4-(4-Chlorophenyl)-2,3,9-trimethyl-6*H*-thieno[3,2-*f*][1,2,4]triazolo[4,3-*a*][1,4]diazepin-6-yl)acetate (OARV-771) (**26**)

Follow General Solution Phase Procedure B using alcohol **52**. Yield: 4.3 mg (22%); ^1^H NMR (CDCl_3_, 400 MHz): δ, ppm 8.67 (1H, s), 7.46 (1H, d, *J* = 8.3 Hz), 7.42–7.28 (8H, m), 7.21 (1H, d, *J* = 8.9 Hz), 5.09 (1H, dq, *J* = 7.1, 7.1 Hz), 4.77 (1H, t, *J* = 7.9 Hz), 4.63–4.51 (3H, m), 4.32 (2H, dd, *J* = 4.3, 7.9 Hz), 4.13 (1H, d, *J* = 11.3 Hz), 3.99 (1H, d, *J* = 15.4 Hz), 3.88 (1H, d, *J* = 15.4 Hz), 3.73–3.56 (9H, m), 2.66 (3H, s), 2.57–2.50 (4H, m), 2.41 (3H, s), 2.11 (1H, dd, *J* = 8.3, 13.7 Hz), 1.94–1.85 (2H, m), 1.69 (3H, s), 1.49 (3H, d, *J* = 7.0 Hz), 1.06 (9H, s); ^13^C NMR (CDCl_3_, 126 MHz): δ, ppm 171.7, 171.7, 170.3, 169.9, 164.0, 155.4, 150.4, 150.1, 148.7, 143.4, 137.0, 136.7, 132.4, 131.8, 131.02, 130.97, 130.5, 130.0, 129.7, 128.8, 126.6, 70.4, 70.3, 68.8, 67.9, 64.0, 58.5, 57.1, 56.8, 53.9, 49.0, 37.0, 35.7, 35.4, 30.0, 26.7, 22.4, 16.3, 14.6, 13.3, 12.0; HRMS *m*/*z* calcd for C_49_H_60_ClN_8_O_8_S_2_ [M + H]^+^ 987.3659, found: 987.3416.

#### 2-((5-(2-(((*S*)-1-((2*S*,4*R*)-4-Hydroxy-2-((4-(4-methylthiazol-5-yl)benzyl)carbamoyl)pyrrolidin-1-yl)-3,3-dimethyl-1-oxobutan-2-yl)amino)-2-oxoethoxy)pentyl)oxy)ethyl 2-((*S*)-4-(4-Chlorophenyl)-2,3,9-trimethyl-6*H*-thieno[3,2-*f*][1,2,4]triazolo[4,3-*a*][1,4]diazepin-6-yl)acetate (OAB1) (**27**)

Follow General Solution Phase Procedure B using alcohol **53**. Yield: 3.9 mg (22%); ^1^H NMR (CDCl_3_, 400 MHz): δ, ppm 8.67 (1H, s), 7.42–7.30 (9H, m), 7.17 (1H, d, *J* = 8.9 Hz), 4.76 (1H, t, *J* = 8.0 Hz), 4.63–4.53 (3H, m), 4.48 (1H, d, *J* = 8.6 Hz), 4.39–4.22 (3H, m), 4.10 (1H, d, *J* = 11.5 Hz), 3.95 (1H, d, *J* = 15.3 Hz), 3.86 (1H, d, *J* = 15.3 Hz), 3.70–3.58 (5H, m), 3.52–3.46 (4H, m), 2.66 (3H, s), 2.60–2.51 (4H, m), 2.41 (3H, s), 2.17 (1H, dd, *J* = 8.4, 13.8 Hz), 1.69 (3H, s), 1.67–1.56 (4H, m), 1.49–1.39 (2H, m), 0.95 (9H, s); ^13^C NMR (CDCl_3_, 126 MHz): δ, ppm 171.7, 171.5, 170.9, 170.5, 164.0, 155.4, 150.4, 150.1, 148.6, 138.3, 137.0, 136.7, 132.4, 131.8, 131.1, 131.0, 130.9, 130.5, 130.0, 129.6, 128.8, 128.3, 71.9, 71.3, 70.3, 70.1, 68.6, 64.1, 58.6, 57.1, 56.7, 53.8, 43.4, 37.0, 36.1, 35.2, 29.40, 29.37, 26.6, 22.8, 16.2, 14.6, 13.3, 12.0; HRMS *m*/*z* calcd for C_50_H_62_ClN_8_O_8_S_2_ [M + H]^+^ 1001.3815, found: 1001.4819.

#### 2-(3-(2-(((*S*)-1-((2*S*,4*R*)-4-Hydroxy-2-((4-(4-methylthiazol-5-yl)benzyl)carbamoyl)pyrrolidin-1-yl)-3,3-dimethyl-1-oxobutan-2-yl)amino)-2-oxoethoxy)propoxy)ethyl 2-((*S*)-4-(4-Chlorophenyl)-2,3,9-trimethyl-6*H*-thieno[3,2-*f*][1,2,4]triazolo[4,3-*a*][1,4]diazepin-6-yl)acetate (OAB2) (**28**)

Follow General Solution Phase Procedure B using alcohol **54**. Yield: 3.0 mg (23%); ^1^H NMR (CDCl_3_, 400 MHz): δ ppm 8.67 (1H, s), 7.42–7.30 (9H, m), 7.18 (1H, d, *J* = 9.0 Hz), 4.81–4.76 (1H, m), 4.62–4.49 (4H, m), 4.35 (1H, dd, *J* = 5.3, 15.0 Hz), 4.28 (2H, t, *J* = 4.9 Hz), 4.10 (1H, d, *J* = 11.2 Hz), 3.99 (1H, d, *J* = 15.3 Hz), 3.84 (1H, d, *J* = 15.5 Hz), 3.71–3.53 (9H, m), 3.44 (1H, br. s), 2.65 (3H, s), 2.57–2.49 (4H, m), 2.41 (3H, s), 2.17 (1H, dd, *J* = 8.1, 13.6 Hz), 1.92–1.81 (2H, m), 1.69 (3H, s), 0.96 (9H, s); ^13^C NMR (CDCl_3_, 126 MHz): δ, ppm 171.6, 171.5, 171.0, 170.2, 164.0, 155.4, 150.3, 150.0, 148.7, 138.5, 137.0, 136.7, 132.4, 131.8, 131.04, 130.98, 130.6, 130.0, 129.6, 128.9, 128.3, 70.4, 68.84, 68.82, 67.9, 64.0, 58.7, 57.1, 56.9, 53.9, 43.4, 37.0, 36.2, 35.4, 30.0, 26.6, 16.2, 14.5, 13.2, 11.9; HRMS *m*/*z* calcd for C_48_H_58_ClN_8_O_8_S_2_ [M + H]^+^ 973.3502, found: 973.4629.

#### Di-*tert*-butyl 2,2′-(Pentane-1,5-diylbis(oxy))diacetate (**32**)

To a stirred solution of 50% NaOH_(aq)_ solution (12 mL), DCM (12 mL), and TBAB (3.09 g, 9.6 mmol) at 5 °C was added pentane-1,5-diol (1 g, 9.6 mmol). This was stirred at 5 °C for 10 min before adding *tert*-butyl bromoacetate (5.62 g, 28.8 mmol) dropwise. This was left to stir vigorously, warming to r.t. over 16 h. After completion shown by TLC, water (20 mL) and pentane (80 mL) were added. The organic layer was separated, washed with brine (50 mL), dried with Na_2_SO_4_, and concentrated *in vacuo*. The residue was purified by flash column chromatography (80 g silica column) using a linear gradient from 5 to 40% EtOAc in heptane to afford **32** as a colorless oil. Yield: 2.11 g (63%); ^1^H NMR (CDCl_3_, 400 MHz): δ, ppm 3.93 (4H, s), 3.51 (4H, t, *J* = 6.6 Hz), 1.69–1.61 (2H, quint., *J =* 7.15 Hz), 1.52–1.41 (20H, m); ^13^C NMR (CDCl_3_, 101 MHz): δ, ppm 170.0, 81.5, 71.7, 69.0, 29.6, 28.3, 22.7.

#### 2,2′-(Pentane-1,5-diylbis(oxy))bis(ethan-1-ol) (**33**)

A solution of 2.4 M LiAlH_4_ in THF (10.08 mL, 24.2 mmol) was added to a N_2_-flushed flask containing anhydrous THF (27 mL) and cooled to 0 °C. Di-*tert*-butyl 2,2′-(pentane-1,5-diylbis(oxy))diacetate (2.11 g, 6.04 mmol) was dissolved in THF (5 mL) and added dropwise. The flask was left to warm to r.t. and stirred for 16 h. The flask was then cooled to 0 °C. Water (1 mL) was then added dropwise; 20% NaOH_(aq)_ solution (0.75 mL) was then added followed by water (3 mL) and left to stir at r.t. for 3 h. MeOH (30 mL) was then added, and the suspension was filtered and concentrated *in vacuo*. The residue was then dissolved in DCM (30 mL) and filtered through PTFE syringe filters. The filtrate was concentrated *in vacuo* to afford **33** as a colorless oil. Yield: 933 mg (74%); ^1^H NMR (CDCl_3_, 400 MHz): δ, ppm 3.75–3.70 (4H, m), 3.55–3.51 (4H, m), 3.49 (4H, t, *J* = 6.3 Hz), 2.26 (2H, t, *J* = 6.2 Hz), 1.67–1.58 (4H, m), 1.51–1.41 (2H, m); ^13^C NMR (CDCl_3_, 126 MHz): δ, ppm 72.0, 71.1, 62.0, 29.3, 22.8.

#### 2,2-Dimethyl-3,3-diphenyl-4,7,10,13-tetraoxa-3-silapentadecan-15-ol (**34**)

Tetraethylene glycol (20.4 g, 105 mmol) was dissolved in anhydrous THF (52.5 mL) under an atmosphere of N_2_. Imidazole (1.36 g, 20 mmol) was added as a solid, and the flask was flushed with N_2_. TBDPSCl (5.50 g, 20 mmol) was then added dropwise, and the reaction was left to stir at r.t. for 16 h. Et_2_O (50 mL) and water (50 mL) were then added, and the organic layer was separated. The aqueous layer was then extracted with Et_2_O (3 × 50 mL), and the combined organic layers were dried with MgSO_4_, filtered, and concentrated *in vacuo*. The residue was purified by flash column chromatography (220 g silica column) using a linear gradient from 30 to 100% EtOAc in heptane to afford **34** as a colorless oil. Yield: 6.91 g (80%); ^1^H NMR (CDCl_3_, 400 MHz): δ, ppm 7.68 (4H, d, *J =* 6.7 Hz), 7.45–7.35 (6H, m), 3.81 (2H, t, *J =* 5.3 Hz), 3.73–3.68 (2H, m), 3.67–3.58 (12H, m), 2.39 (1H, t, *J =* 6.2 Hz), 1.05 (9H, s); ^13^C NMR (CDCl_3_, 101 MHz): δ, ppm 135.8, 133.9, 129.7, 127.8, 72.63, 72.61, 70.93, 70.85, 70.6, 63.6, 62.0, 27.0, 19.3; LC-MS *m*/*z* calcd for C_24_H_36_NaO_5_Si [M + Na]^+^ 455.2, found: 455.2.

#### 2,2-Dimethyl-3,3-diphenyl-4,7,11-trioxa-3-silatridecan-13-ol (**35**)

Follow General Solution Phase Procedure C using 3,7-dioxa-1,9-nonanediol. Yield: 600 mg (49%); ^1^H NMR (CDCl_3_, 400 MHz): δ, ppm 7.69 (4H, d, *J =* 6.7 Hz), 7.45–7.35 (6H, m), 3.80 (2H, t, *J =* 5.2 Hz), 3.71 (2H, dt, *J =* 5.1, 4.9 Hz), 3.60–3.51 (8H, m), 2.03 (1H, t, *J =* 6.0 Hz), 1.85 (2H, quint., *J =* 6.3 Hz), 1.05 (9H, s); ^13^C NMR (CDCl_3_, 101 MHz): δ, ppm 135.8, 134.0, 129.7, 127.8, 72.3, 71.9, 68.4, 68.4, 63.6, 62.0, 30.2, 27.0, 19.4; LC-MS *m*/*z* calcd for C_23_H_34_NaO_4_Si [M + Na]^+^ 425.2, found: 425.1.

#### 2,2-Dimethyl-3,3-diphenyl-4,7,13-trioxa-3-silapentadecan-15-ol (**36**)

Follow General Solution Phase Procedure C using diol **33**. Yield: 654 mg (57%); ^1^H NMR (CDCl_3_, 400 MHz): δ, ppm 7.69 (4H, dd, *J* = 1.2, 7.5 Hz), 7.44–7.34 (6H, m), 3.80 (2H, t, *J* = 5.4 Hz), 3.74–3.69 (2H, m), 3.56–3.51 (4H, m), 3.47 (4H, dt, *J* = 4.4, 6.5 Hz), 1.93 (1H, t, *J* = 6.1 Hz), 1.66–1.54 (4H, m), 1.47–1.37 (2H, m), 1.05 (9H, s); ^13^C NMR (CDCl_3_, 101 MHz): δ, ppm 135.8, 134.0, 129.7, 127.7, 72.2, 71.9, 71.4, 63.7, 62.0, 29.8, 29.7, 27.0, 22.9, 19.4; LC-MS *m*/*z* calcd for C_25_H_38_NaO_4_Si [M + Na]^+^ 453.2, found: 453.2.

#### 2-(3-(2-Azidoethoxy)propoxy)ethan-1-ol (**37**)

Follow General Solution Phase Procedure D using 3,7-dioxa-1,9-nonanediol. Yield: 361 mg (63%); ^1^H NMR (CDCl_3_, 400 MHz): δ, ppm 3.73 (2H, td, *J =* 4.7, 5.9 Hz), 3.64–3.54 (8H, m), 3.37 (2H, t, *J =* 5.0 Hz), 1.99 (1H, t, *J =* 6.1 Hz), 1.89 (2H, quint., *J =* 6.2 Hz); ^13^C NMR (CDCl_3_, 101 MHz): δ, ppm 72.0, 69.9, 68.3, 68.1, 62.0, 50.9, 30.1.

#### 2-((5-(2-Azidoethoxy)pentyl)oxy)ethan-1-ol (**38**)

Follow General Solution Phase Procedure D using diol **33**. Yield: 244 mg (52%); ^1^H NMR (CDCl_3_, 400 MHz): δ, ppm 3.72 (2H, dt, *J* = 5.1, 4.9 Hz), 3.61 (2H, t, *J* = 5.0 Hz), 3.53 (2H, t, *J* = 4.6 Hz), 3.49 (4H, t, *J* = 6.5 Hz), 3.36 (2H, t, *J* = 5.0 Hz), 1.96 (1H, t, *J* = 6.2 Hz), 1.62 (4H, quint., *J* = 7.1 Hz), 1.50–1.40 (2H, m); ^13^C NMR (CDCl_3_, 126 MHz): δ, ppm 71.9, 71.4, 71.3, 69.7, 62.0, 50.9, 29.6, 29.6, 22.8.

#### 2,2-Dimethyl-3,3-diphenyl-4,7,10,13-tetraoxa-3-silapentadecan-15-oic Acid (**39**)

Follow General Solution Phase Procedure E using alcohol **34**. Yield: 622 mg (70%); ^11^H NMR (CDCl_3_, 400 MHz): δ, ppm 7.69 (4H, d, *J =* 7.2 Hz), 7.45–7.35 (6H, m), 4.13 (2H, s), 3.82 (2H, t, *J =* 5.2 Hz), 3.76–3.67 (8H, m), 3.61 (2H, t, *J =* 5.2 Hz), 1.05 (9H, s); ^13^C NMR (CDCl_3_, 101 MHz): δ, ppm 135.8, 133.8, 129.8, 127.8, 72.7, 71.9, 71.0, 70.7, 70.2, 63.7, 27.0, 19.3; LC-MS *m*/*z* calcd for C_24_H_34_NaO_6_Si [M + Na]^+^ 469.2, found: 469.1.

#### 2,2-Dimethyl-3,3-diphenyl-4,7,11-trioxa-3-silatridecan-13-oic Acid (**40**)

Follow General Solution Phase Procedure E using alcohol **35**. Yield: 264 mg (85%); ^1^H NMR (CDCl_3_, 400 MHz): δ, ppm 7.68 (4H, d, *J =* 6.8 Hz), 7.45–7.36 (6H, m), 4.04 (2H, s), 3.81 (2H, t, *J =* 5.2 Hz), 3.65 (4H, dt, *J =* 6.0, 6.0 Hz), 3.58 (2H, t, *J =* 5.2 Hz), 1.88 (2H, quint., *J =* 5.9 Hz), 1.05 (9H, s); ^13^C NMR (CDCl_3_, 101 MHz): δ, ppm 135.8, 133.8, 129.8, 127.8, 72.5, 70.2, 69.0, 68.3, 63.4, 29.5, 27.0, 19.4; LC-MS *m*/*z* calcd for C_23_H_32_NaO_5_Si [M + Na]^+^ 439.2, found: 439.1.

#### 2,2-Dimethyl-3,3-diphenyl-4,7,13-trioxa-3-silapentadecan-15-oic Acid (**41**)

Follow General Solution Phase Procedure E using alcohol **36**. Yield: 288 mg (85%); ^1^H NMR (CDCl_3_, 400 MHz): δ, ppm 9.00 (1H, br. s), 7.69 (4H, dd, *J* = 1.4, 7.7 Hz), 7.44–7.34 (6H, m), 4.07 (2H, s), 3.80 (2H, t, *J* = 5.4 Hz), 3.59–3.52 (4H, m), 3.47 (2H, t, *J* = 6.3 Hz), 1.70–1.55 (4H, m), 1.49–1.40 (2H, m), 1.05 (9H, s); ^13^C NMR (CDCl_3_, 101 MHz): δ, ppm 135.6, 133.8, 129.6, 127.6, 72.1, 71.1, 67.8, 63.5, 29.5, 29.2, 26.8, 22.6, 19.2; LC-MS *m*/*z* calcd for C_25_H_36_NaO_5_Si [M + Na]^+^ 467.2, found: 467.2.

#### 2-(3-(2-Azidoethoxy)propoxy)acetic Acid (**42**)

Follow General Solution Phase Procedure E using alcohol **37**. Yield: 273 mg (65%); ^1^H NMR (CDCl_3_, 500 MHz): δ, ppm 4.10 (2H, s), 3.72 (2H, t, *J =* 6.0 Hz), 3.67–3.64 (4H, m), 3.39 (2H, t, *J =* 5.0 Hz), 1.94 (2H, quint., *J =* 5.9 Hz); ^13^C NMR (CDCl_3_, 126 MHz): δ, ppm 70.1, 69.6, 68.6, 68.2, 50.7, 29.6.

#### 2-((5-(2-Azidoethoxy)pentyl)oxy)acetic Acid (**43**)

Follow General Solution Phase Procedure E using alcohol **38**. Yield: 78 mg (74%); ^1^H NMR (CDCl_3_, 400 MHz): δ, ppm 9.84 (1H, br. s), 4.08 (2H, s), 3.58 (2H, t, *J* = 5.1 Hz), 3.53 (2H, t, *J* = 6.6 Hz), 3.46 (2H, t, *J* = 6.4 Hz), 3.33 (2H, t, *J* = 5.0 Hz), 1.68–1.55 (4H, m), 1.47–1.38 (2H, m); ^13^C NMR (CDCl_3_, 101 MHz): δ, ppm 174.8, 71.9, 71.2, 69.5, 67.8, 50.8, 29.3, 29.2, 22.5.

#### (2*S*,4*R*)-1-((*S*)-17-(*tert*-Butyl)-2,2-dimethyl-15-oxo-3,3-diphenyl-4,7,10,13-tetraoxa-16-aza-3-silaoctadecan-18-oyl)-4-hydroxy-*N*-(4-(4-methylthiazol-5-yl)benzyl)pyrrolidine-2-carboxamide (**44**)

Follow General Solution Phase Procedure F using carboxylic acid **39** and VHL-NH_3_Cl. Yield: 108 mg (55%); ^1^H NMR (CDCl_3_, 400 MHz): δ, ppm 8.66 (1H, s), 7.67 (4H, d, *J =* 6.9 Hz), 7.44–7.27 (11H, m), 4.72 (1H, t, *J =* 7.8 Hz), 4.57–4.49 (2H, m), 4.46 (1H, d, *J =* 8.4 Hz), 4.32 (1H, dd, *J =* 5.3, 15.0 Hz), 4.08 (1H, d, *J =* 11.1 Hz), 4.00 (1H, d, *J =* 15.8 Hz), 3.94 (1H, d, *J =* 15.7 Hz), 3.79 (2H, t, *J =* 5.3 Hz), 3.66–3.55 (11H, m), 2.58–2.50 (4H, m), 2.09 (1H, dd, *J =* 8.5, 13.5 Hz), 1.04 (9H, s), 0.94 (9H, s); ^13^C NMR (CDCl_3_, 101 MHz): δ, ppm 170.9, 170.6, 150.4, 148.6, 138.3, 135.7, 133.8, 131.7, 131.0, 129.7, 129.6, 128.2, 127.7, 72.6, 71.3, 70.9, 70.8, 70.5, 70.4, 70.2, 63.5, 58.6, 57.3, 56.7, 43.3, 36.0, 35.0, 26.9, 26.5, 19.3, 16.1; LC-MS *m*/*z* calcd for C_46_H_63_N_4_O_8_SSi [M + H]^+^ 859.4, found: 859.1.

#### (2*S*,4*R*)-1-((*S*)-2-(2-(3-(2-Azidoethoxy)propoxy)acetamido)-3,3-dimethylbutanoyl)-4-hydroxy-*N*-((*S*)-1-(4-(4-methylthiazol-5-yl)phenyl)ethyl)pyrrolidine-2-carboxamide (**45**)

Follow General Solution Phase Procedure F using carboxylic acid **42** and Me-VHL-NH_3_Cl. Yield: 70 mg (53%); ^1^H NMR (CDCl_3_, 500 MHz): δ, ppm 8.65 (1H, s), 7.56 (1H, d, *J* = 8.0 Hz), 7.38 (2H, d, *J* = 8.4 Hz), 7.34 (2H, d, *J* = 8.3 Hz), 7.18 (1H, d, *J* = 8.1 Hz), 5.06 (1H, dq, *J* = 7.1, 7.1 Hz), 4.68 (1H, t, *J* = 8.0 Hz), 4.49 (1H, s), 4.46 (1H, d, *J* = 8.1 Hz), 4.01 (1H, d, *J* = 11.7 Hz), 3.93 (2H, dd, *J* = 15.6, 16.8 Hz), 3.66–3.50 (7H, m), 3.32 (2H, dd, *J* = 4.4, 5.4 Hz), 2.50 (3H, s), 2.39–2.30 (1H, m), 2.07–1.99 (1H, m), 1.86 (2H, quint., *J* = 6.2 Hz), 1.47 (3H, d, *J* = 6.8 Hz), 1.05 (9H, s); ^13^C NMR (CDCl_3_, 126 MHz): δ, ppm 171.3, 170.7, 170.1, 150.4, 148.6, 143.5, 131.7, 130.9, 129.6, 126.5, 70.2, 70.0, 69.8, 68.8, 67.8, 58.8, 57.5, 56.7, 50.7, 49.0, 36.1, 35.1, 29.9, 26.6, 22.4, 16.1; LC-MS *m*/*z* calcd for C_30_H_44_N_7_O_6_S [M + H]^+^ 630.3, found: 630.3.

#### (2*S*,4*R*)-1-((*S*)-15-(*tert*-Butyl)-2,2-dimethyl-13-oxo-3,3-diphenyl-4,7,11-trioxa-14-aza-3-silahexadecan-16-oyl)-4-hydroxy-*N*-((*S*)-1-(4-(4-methylthiazol-5-yl)phenyl)ethyl)pyrrolidine-2-carboxamide (**46**)

Follow General Solution Phase Procedure F using carboxylic acid **40** and Me-VHL-NH_3_Cl. Yield: 17 mg (66%); ^1^H NMR (CDCl_3_, 400 MHz): δ, ppm 8.66 (1H, s), 7.68 (4H, dd, *J* = 1.6, 7.8 Hz), 7.46–7.35 (11H, m), 7.19 (1H, d, *J* = 8.5 Hz), 5.08 (1H, dq, *J* = 7.1, 7.2 Hz), 4.74 (1H, t, *J* = 7.8 Hz), 4.54–4.51 (2H, m), 4.09 (1H, d, *J* = 11.4 Hz), 3.92 (2H, s), 3.81–3.78 (2H, m), 3.64–3.53 (7H, m), 2.62–2.52 (4H, m), 2.04 (1H, dd, *J* = 8.7, 13.3 Hz), 1.87 (2H, quint., *J* = 6.3 Hz), 1.47 (3H, d, *J* = 7.1 Hz), 1.06–1.02 (18H, m); ^13^C NMR (CDCl_3_, 101 MHz): δ, ppm 171.8, 170.5, 169.7, 150.4, 148.7, 143.3, 135.7, 133.9, 131.7, 131.0, 129.75, 129.70, 127.7, 126.6, 72.3, 70.2, 69.1, 67.9, 63.6, 58.5, 57.1, 56.7, 49.0, 35.5, 35.1, 30.1, 27.0, 26.6, 22.4, 19.3, 16.2; LC-MS *m*/*z* calcd for C_46_H_63_N_4_O_7_SSi [M + H]^+^ 843.4, found: 843.3.

#### (2*S*,4*R*)-1-((*S*)-2-(2-((5-(2-Azidoethoxy)pentyl)oxy)acetamido)-3,3-dimethylbutanoyl)-4-hydroxy-*N*-(4-(4-methylthiazol-5-yl)benzyl)pyrrolidine-2-carboxamide (**47**)

Follow General Solution Phase Procedure F using carboxylic acid **43** and VHL-NH_3_Cl. Yield: 31 mg (56%); ^1^H NMR (CDCl_3_, 400 MHz): δ, ppm 8.66 (1H, s), 7.40–7.31 (5H, m), 7.16 (1H, d, *J* = 8.9 Hz), 4.72 (1H, t, *J* = 7.7 Hz), 4.56–4.47 (3H, m), 4.35 (1H, d, *J* = 5.3 Hz), 4.32 (1H, d, *J* = 5.2 Hz), 4.05 (1H, d, *J* = 11.4 Hz), 3.91 (1H, d, *J* = 15.4 Hz), 3.86 (1H, d, *J* = 15.4 Hz), 3.62 (2H, dd, *J* = 3.8, 11.2 Hz), 3.58 (1H, t, *J* = 5.0 Hz), 3.51–3.44 (4H, m), 3.33 (2H, t, *J* = 5.0 Hz), 2.55–2.48 (4H, m), 2.09 (1H, dd, *J* = 8.0, 13.6 Hz), 1.66–1.57 (4H, m), 1.47–1.39 (2H, m), 0.93 (9H, s). ^13^C NMR (CDCl_3_, 101 MHz): δ, ppm 171.5, 170.8, 170.5, 150.4, 148.6, 138.3, 131.7, 131.1, 129.6, 128.2, 71.9, 71.2, 70.2, 70.1, 69.7, 58.6, 57.0, 56.8, 50.9, 43.4, 36.0, 35.1, 29.5, 29.4, 26.5, 22.7, 16.1; LC-MS *m*/*z* calcd for C_31_H_46_N_7_O_6_S [M + H]^+^ 644.3, found: 644.7.

#### (2*S*,4*R*)-1-((*S*)-17-(*tert*-Butyl)-2,2-dimethyl-15-oxo-3,3-diphenyl-4,7,13-trioxa-16-aza-3-silaoctadecan-18-oyl)-4-hydroxy-*N*-(4-(4-methylthiazol-5-yl)benzyl)pyrrolidine-2-carboxamide (**48**)

Follow General Solution Phase Procedure F using carboxylic acid **41** and VHL-NH_3_Cl. Yield: 44 mg (61%); ^1^H NMR (CDCl_3_, 400 MHz): δ, ppm 8.66 (1H, s), 7.68 (4H, dd, *J* = 1.3, 7.6 Hz), 7.43–7.30 (11H, m), 7.17 (1H, d, *J* = 8.7 Hz), 4.73 (1H, t, *J* = 7.8 Hz), 4.57–4.47 (3H, m), 4.34 (1H, dd, *J* = 5.1, 14.7 Hz), 4.07 (1H, d, *J* = 11.3 Hz), 3.92 (1H, d, *J* = 15.4 Hz), 3.87 (1H, d, *J* = 15.2 Hz), 3.79 (2H, t, *J* = 5.3 Hz), 3.62 (1H, dd, *J* = 3.7, 11.3 Hz), 3.54–3.41 (7H, m), 2.58–2.50 (4H, m), 2.09 (1H, dd, *J* = 8.1, 13.7 Hz), 1.67–1.54 (4H, m), 1.46–1.35 (2H, m); ^13^C NMR (CDCl_3_, 101 MHz): δ, ppm 171.5, 170.8, 170.6, 150.4, 148.6, 138.2, 135.7, 133.9, 131.7, 131.1, 129.7, 129.6, 128.2, 127.7, 72.2, 72.0, 71.3, 70.2, 70.1, 63.6, 58.6, 57.1, 56.7, 43.4, 35.9, 35.0, 29.7, 29.4, 27.0, 26.5, 22.8, 19.3, 16.1; LC-MS *m*/*z* calcd for C_47_H_65_N_4_O_7_SSi [M + H]^+^ 857.4, found: 857.3.

#### (2*S*,4*R*)-1-((*S*)-2-(2-(3-(2-Azidoethoxy)propoxy)acetamido)-3,3-dimethylbutanoyl)-4-hydroxy-*N*-(4-(4-methylthiazol-5-yl)benzyl)pyrrolidine-2-carboxamide (**49**)

Follow General Solution Phase Procedure F using carboxylic acid **42** and VHL-NH_3_Cl. Yield: 28 mg (53%); ^1^H NMR (CDCl_3_, 400 MHz): δ, ppm 8.67 (1H, s), 7.39–7.33 (5H, m), 7.15 (1H, d, *J* = 8.6 Hz), 4.74 (1H, t, *J* = 7.8 Hz), 4.59–4.48 (3H, m), 4.35 (1H, dd, *J* = 5.2, 14.9 Hz), 4.09 (1H, d, *J* = 11.2 Hz), 3.95 (1H, d, *J* = 15.4 Hz), 3.90 (1H, d, *J* = 15.3 Hz), 3.65–3.54 (7H, m), 3.34 (2H, t, *J* = 4.9 Hz), 2.62–2.54 (1H, m), 2.51 (3H, s), 2.11 (1H, dd, *J* = 8.2, 13.5 Hz), 1.89 (2H, quint., *J* = 6.2 Hz), 0.95 (9H, s); ^13^C NMR (CDCl_3_, 101 MHz): δ, ppm 171.6, 170.7, 170.5, 150.4, 148.7, 138.2, 131.7, 131.2, 129.7, 128.3, 70.3, 70.2, 69.9, 68.7, 67.9, 58.5, 57.1, 56.7, 50.8, 43.4, 35.8, 35.0, 30.0, 26.5, 16.2; LC-MS *m*/*z* calcd for C_29_H_42_N_7_O_6_S [M + H]^+^ 616.3, found: 616.2.

#### (2*S*,4*R*)-1-((*S*)-15-(*tert*-Butyl)-2,2-dimethyl-13-oxo-3,3-diphenyl-4,7,11-trioxa-14-aza-3-silahexadecan-16-oyl)-4-hydroxy-*N*-(4-(4-methylthiazol-5-yl)benzyl)pyrrolidine-2-carboxamide (**50**)

Follow General Solution Phase Procedure F using carboxylic acid **40** and VHL-NH_3_Cl. Yield: 40 mg (57%); ^1^H NMR (CDCl_3_, 400 MHz): δ, ppm 8.66 (1H, s), 7.68 (4H, dd, *J* = 1.0, 7.5 Hz), 7.44–7.30 (11H, m), 7.16 (1H, d, *J* = 8.7 Hz), 4.72 (1H, t, *J* = 7.8 Hz), 4.57–4.47 (3H, m), 4.33 (1H, dd, *J* = 5.3, 15.0 Hz), 4.06 (1H, d, *J* = 11.5 Hz), 3.91 (1H, d, *J* = 16.2 Hz), 3.87 (1H, d, *J* = 15.3 Hz), 3.79 (2H, t, *J* = 5.2 Hz), 3.65–3.51 (7H, m), 2.59–2.50 (4H, m), 2.10 (1H, dd, *J* = 8.0, 13.3 Hz), 1.85 (2H, quint., *J* = 6.4 Hz), 1.04 (9H, s), 0.94 (9H, s); ^13^C NMR (CDCl_3_, 101 MHz): δ, ppm 171.5, 170.8, 170.5, 150.4, 148.6, 138.2, 135.7, 133.9, 131.7, 131.1, 129.7, 129.6, 128.3, 127.7, 72.3, 70.24, 70.16, 69.1, 67.9, 63.6, 58.6, 57.1, 56.7, 43.4, 35.9, 35.1, 30.1, 27.0, 26.5, 19.3, 16.1; LC-MS *m*/*z* calcd for C_45_H_61_N_4_O_7_SSi [M + H]^+^ 829.4, found: 829.3.

#### (2*S*,4*R*)-1-((*S*)-2-(*tert*-Butyl)-14-hydroxy-4-oxo-6,9,12-trioxa-3-azatetradecanoyl)-4-hydroxy-*N*-(4-(4-methylthiazol-5-yl)benzyl)pyrrolidine-2-carboxamide (**51**)

Follow General Solution Phase Procedure G using silyl ether **44**. Yield: 39 mg (quant.); ^1^H NMR (500 MHz, CDCl_3_): δ, ppm 8.67 (1H, s), 7.48 (1H, t, *J =* 5.8 Hz), 7.38–7.32 (5H, m), 4.70 (1H, t, *J =* 7.9 Hz), 4.58–4.51 (3H, m), 4.34 (1H, dd, *J =* 5.4, 15.0 Hz), 4.09–4.00 (3H, m), 3.71–3.59 (12H, m), 3.58–3.49 (2H, m), 2.51–2.45 (4H, m), 2.12 (1H, dd, *J =* 8.0, 13.4 Hz), 0.96 (9H, s); ^13^C NMR (CDCl_3_, 101 MHz): δ, ppm 171.2, 171.1, 170.6, 150.4, 148.5, 138.4, 131.7, 130.9, 129.5, 128.2, 72.7, 71.0, 70.8, 70.7, 70.5, 70.3, 70.2, 61.7, 58.7, 57.0, 56.9, 43.3, 36.4, 35.5, 26.5, 16.1; LC-MS *m*/*z* calcd for C_30_H_46_N_4_O_8_S [M + H]^+^ 621.3, found: 621.3.

#### (2*S*,4*R*)-4-Hydroxy-1-((*S*)-2-(2-(3-(2-hydroxyethoxy)propoxy)acetamido)-3,3-dimethylbutanoyl)-*N*-((*S*)-1-(4-(4-methylthiazol-5-yl)phenyl)ethyl)pyrrolidine-2-carboxamide (**52**)

Follow General Solution Phase Procedure G using silyl ether **46**. Yield: 15 mg (83%); ^1^H NMR (CDCl_3_, 400 MHz): δ, ppm 8.66 (1H, s), 7.50 (1H, d, *J* = 7.9 Hz), 7.40 (2H, d, *J* = 8.7 Hz), 7.36 (2H, d, *J* = 8.6 Hz), 7.23 (1H, d, *J* = 9.3 Hz), 5.08 (1H, dq, *J* = 7.1, 7.2 Hz), 4.67 (1H, t, *J* = 7.8 Hz), 4.58 (1H, d, *J* = 9.1 Hz), 4.50 (1H, s), 4.05–3.99 (2H, m), 3.88 (1H, d, *J* = 15.6 Hz), 3.76–3.51 (10H, m), 2.52 (3H, s), 2.48–2.39 (1H, m), 2.04 (1H, dd, *J* = 8.2, 13.6 Hz), 1.93–1.84 (2H, m), 1.48 (3H, d, *J* = 6.8 Hz), 1.05 (9H, s); ^13^C NMR (CDCl_3_, 101 MHz): δ, ppm 171.5, 170.6, 170.0, 150.4, 148.6, 143.4, 131.7, 131.0, 129.6, 126.6, 72.2, 70.3, 70.2, 69.2, 67.7, 61.8, 58.8, 57.1, 56.9, 49.0, 36.1, 35.5, 29.9, 26.6, 22.3, 16.2; LC-MS *m*/*z* calcd for C_30_H_45_N_4_O_7_S [M + H]^+^ 605.3, found: 605.2.

#### (2*S*,4*R*)-4-Hydroxy-1-((*S*)-2-(2-((5-(2-hydroxyethoxy)pentyl)oxy)acetamido)-3,3-dimethylbutanoyl)-*N*-(4-(4-methylthiazol-5-yl)benzyl)pyrrolidine-2-carboxamide (**53**)

Follow General Solution Phase Procedure G using silyl ether **48**. Yield: 11 mg (73%); ^1^H NMR (CDCl_3_, 500 MHz): δ, ppm 8.67 (1H, s), 7.55 (1H, t, *J* = 5.6 Hz), 7.38–7.31 (4H, m), 7.20 (1H, d, *J* = 8.7 Hz), 4.72 (1H, t, *J* = 7.7 Hz), 4.58–4.51 (3H, m), 4.34 (1H, dd, *J* = 5.3, 14.9 Hz), 4.03 (1H, d, *J* = 11.3 Hz), 3.94 (1H, d, *J* = 15.7 Hz), 3.87 (1H, d, *J* = 15.7 Hz), 3.69–3.63 (3H, m), 3.56–3.45 (6H, m), 3.31–3.27 (1H, m), 2.55–2.48 (4H, m), 2.12 (1H, dd, *J* = 8.2, 13.4 Hz), 1.69–1.58 (4H, m), 1.51–1.41 (2H, m), 0.95 (9H, s); ^13^C NMR (CDCl_3_, 126 MHz): δ, ppm 171.3, 171.0, 170.5, 150.4, 148.6, 138.4, 131.8, 131.1, 129.6, 128.3, 72.0, 71.9, 71.1, 70.3, 70.1, 61.9, 58.6, 56.93, 56.88, 43.4, 36.2, 35.4, 29.6, 29.4, 26.5, 23.1, 16.2; LC-MS *m*/*z* calcd for C_31_H_47_N_4_O_7_S [M + H]^+^ 619.3, found: 619.3.

#### (2*S*,4*R*)-4-Hydroxy-1-((*S*)-2-(2-(3-(2-hydroxyethoxy)propoxy)acetamido)-3,3-dimethylbutanoyl)-*N*-(4-(4-methylthiazol-5-yl)benzyl)pyrrolidine-2-carboxamide (**54**)

Follow General Solution Phase Procedure G using silyl ether **50**. Yield: 11 mg (79%); ^1^H NMR (CDCl_3_, 400 MHz): δ, ppm 8.67 (1H, s), 7.45 (1H, t, *J* = 5.8 Hz), 7.36 (4H, dd, *J* = 9.1, 9.1 Hz), 7.17 (1H, d, *J* = 9.1 Hz), 4.69 (1H, t, *J* = 7.9 Hz), 4.61–4.52 (3H, m), 4.33 (1H, dd, *J* = 5.3, 15.0 Hz), 4.06 (1H, d, *J* = 11.6 Hz), 4.02 (1H, d, *J* = 15.8 Hz), 3.87 (1H, d, *J* = 15.6 Hz), 3.70–3.46 (9H, m), 2.55–2.48 (4H, m), 2.12 (1H, dd, *J* = 8.1, 13.4 Hz), 1.89 (2H, quint., *J* = 6.1 Hz), 0.96 (9H, s); ^13^C NMR (CDCl_3_, 101 MHz): δ, ppm 171.5, 170.9, 170.6, 150.4, 148.7, 138.4, 131.8, 131.1, 129.6, 128.3, 72.1, 70.34, 70.28, 69.2, 67.7, 61.9, 58.7, 57.1, 56.9, 43.4, 36.3, 35.2, 29.9, 26.6, 16.2; LC-MS *m*/*z* calcd for C_29_H_43_N_4_O_7_S [M + H]^+^ 591.3, found: 591.2.

#### Parallel Artificial Membrane Permeability Assay (PAMPA)

PAMPA^43,79^ was used to determine the passive membrane permeability, as described in Naylor et al.^41^ and Klein et al. (2020).^31^

#### Log *D*_(dec/w)_ Shake Flask Partition Coefficient Assay

The shake flask partition coefficient of each compound was determined following the procedure described in Klein et al. (2020).^31^

#### Lipophilic Permeability Efficiency (LPE) Metric Calculations

LPE was calculated using the protocol described in Naylor et al.^41^ using the following equation: LPE = Log *D*_(dec/w)_ – 1.06(ALog *P*) + 5.47.

#### Bidirectional MDCK-MDR1 Cell Permeability

Bidirectional MDCK-MDR1 cell permeability data were collected by the CRO Quintara Discovery Inc., San Francisco, CA.

#### Cell Culture

All cell lines employed in this study were obtained from ATCC. HEK293 was cultured in Dulbecco’s modified Eagle’s medium (Gibco, 31966021) supplemented with 10% fetal bovine serum (FBS) and 1% Pen/Strep. MV4;11 cell line was cultured in Iscove’s modified Dulbecco’s medium (Gibco, 21980032) supplemented with 10% FBS and 1% Pen/Strep. 22Rv1 cell line was cultured in RPMI-1640 (Gibco, 11875093) media supplemented with 10% FBS and 1% Pen/Strep. All cell lines were maintained in a humidified incubator at 37 °C and 5% CO_2_.

#### Western Blot Analysis

Cells were seeded (HEK293: 1 × 10^5^ cells/well) in 12-well plates. Following compound treatment, cells were lysed on ice with RIPA lysis and extraction buffer (Thermo Fisher Scientific, 89901) supplemented with protease inhibitor cocktail (Merck, 11697498001) and Benzonase Nuclease (Sigma-Aldrich, E1014). Protein concentration was determined using the BCA assay (Thermo Fisher Scientific, 23225). Samples were then prepared and loaded onto NuPAGE 4–12% Bis–Tris Midi gels (Thermo Fisher Scientific, WG1403A) followed by the transfer of the proteins onto nitrocellulose membranes (EMD Millipore). The membranes were blocked for 1 h prior to incubation with the primary antibodies using 5% Milk TBST. Membranes were probed for Brd2 (Abcam, Ab139690, 1:1000), Brd3 (Abcam, Ab50818, 1:4000), and Brd4 (Abcam, Ab128874, 1:1000). Following overnight incubation with the primary antibodies at 4 °C, the membranes were incubated with secondary antibodies (Anti-rabbit, Abcam AB216773, 1:5000 or antimouse, Abcam AB216774, 1:5000) and hFAB Rhodamine Anti-Tubulin Antibody (Bio-Rad, 12004165, 1:10 000) for 1 h and then imaged with a Bio-Rad imager (LI-COR Biosciences). All Western blots were analyzed for band intensities using Image Lab from Bio-Rad (LI-COR Biosciences). The data extracted from these blots were then subsequently plotted and analyzed using Prism (v. 8.2.1, GraphPad).

#### Cell Viability Assay

MV4;11 cells were plated on 96-well, white-bottom plates and grown for 16 h at 37 °C prior to treatment in IMDM media supplemented with 10% FBS and penicillin/streptomycin. 22Rv1 cells were plated on 96-well, clear-bottom plates and grown for 16 h at 37 °C prior to treatment in RMPI media supplemented with 10% FBS and penicillin/streptomycin. Wells containing just media were also included for blank correction. The initial cell density was 1 × 10^6^ per mL at a volume of 50 μL per well for both cell lines (5 × 10^5^ cells per well). Cells were treated with compounds in duplicate (triplicate for DMSO controls) at a 2× concentration in 0.2% DMSO. Compounds were serially diluted in Eppendorf tubes (7-point, 10-fold serial dilution). Cells were treated with 50 μL of compound for a final concentration of 10 μM:10 pM in 0.1% DMSO. Cells were incubated at 37 °C; 24 h for MV4;11 cells; 72 h for 22Rv1 cells. Then, 100 μL of Promega CellTiter-Glo 2.0 Cell Viability Assay reagent was added to each well according to the manufacturer’s instructions. Plates were subjected to 2 min on an orbital shaker to encourage lysis and left for a further 8 min to reach maximal luminescence. Luminescence was then recorded on a BMG Labtech PHERAstar luminescence plate reader with recommended settings. Data were analyzed with Prism (v. 9.1.0, GraphPad) and normalized to the DMSO vehicle control. EC_50_ values were derived from this plot.

#### Protein Expression and Purification

VCB (VHL:ElonginC:ElonginB) was expressed and purified as described previously.^14^ Briefly, N-terminally His6-tagged VHL (54–213), ElonginC (17–112), and ElonginB (1–104) were coexpressed in *Escherichia coli* and the complex was isolated using Ni-affinity chromatography using TEV protease to remove His6 Tag. The complex was further purified by anion exchange followed by gel filtration chromatography. The BET bromodomains were expressed and purified as described previously.^14^ Briefly, N-terminally His6-tagged Brd2-BD1 (71–194), Brd2-BD2 (344–455), Brd3-BD1 (24–146), Brd3-BD2 (306–416), Brd4-BD1 (44–178), and Brd4-BD2 (333–460) were expressed in *E. coli* and isolated by Ni-affinity chromatography using TEV protease to remove His6 Tag followed by gel filtration chromatography.

#### Fluorescence Polarization (FP) Binding Assay

FP competitive binding assays were performed as described previously,^34,80^ with all measurements taken using a PHERAstar FS (BMG LABTECH) with fluorescence excitation and emission wavelengths (λ) of 485 and 520 nm, respectively. Assays were run in triplicate using 384-well plates (Corning 3544), with each well solution containing 15 nM VCB protein, 10 nM 5,6-carboxyfluorescein (FAM)-labeled HIF-1α peptide (FAM-DEALAHypYIPMDDDFQLRSF, “JC9”), and decreasing concentrations of VHL ligands (14-point, 2-fold serial dilution starting from 100 μM VHL ligand) or PROTACs (14-point, 2-fold serial dilution starting from 20 μM PROTAC) or PROTACs:bromodomain (14-point, 2-fold serial dilution starting from 20 μM PROTAC: 50 μM bromodomain into buffer containing 10 μM of bromodomain). All components were dissolved from stock solutions using 100 mM Bis–Tris propane, 100 mM NaCl, 1 mM DTT, pH 7.0, to yield a final assay volume of 15 μL. DMSO was added as appropriate to ensure a final concentration of 2% v/v. Control wells containing VCB and JC9 with no compound (zero displacement) or JC9 in the absence of protein (maximum displacement) were also included to allow for normalization. Percentage displacement values were obtained by the normalization of controls and were plotted against Log[Compound]. IC_50_ values were determined for each titration using nonlinear regression analysis with Prism (v. 9.1.0, GraphPad). *K*_i_ values were back-calculated from the *K*_d_ for JC9 (∼1.5–2.5 nM determined from direct binding) and fitted IC_50_ values, as described previously.^59,80^ Cooperativity values (α) for each PROTAC were calculated using the ratio: α = *K*_d_ (− bromodomain)/*K*_d_(+ bromodomain).

## Supporting Information Available

Supplementary figures and tables, physicochemical properties of compounds, MDCK permeability data, FP binding data, plasma stability data, Western blot data with uncropped blots, degradation profile data, HPLC traces for compounds **1**–**28**, and liposcan selected ion chromatograms for compounds **1**–**20** (PDF)
Molecular strings for compounds **1**–**28** with associated biochemical and biological data (CSV)

## Abbreviations Used

ACN: acetonitrile
BD1: first bromodomain
BD2: second bromodomain
BET: bromodomain and extra terminal
DAIB: (diacetoxyiodo)benzene
DIC: *N*,*N*′-diisopropylcarbodiimide
DIPEA: *N*,*N*-diisopropylethylamine
HATU: 1-[bis(dimethylamino)methylene]-1*H*-1,2,3-triazolo[4,5-*b*]pyridinium 3-oxide hexafluorophosphate
HF/pyridine: hydrogen fluoride pyridine
PROTAC: proteolysis targeting chimera
*p*-TsCl: *p*-tosyl chloride
TBDPSCl: *tert*-butyldiphenylchlorosilane
TEA: triethylamine
TEMPO: 2,2,6,6-tetramethyl-1-piperidinyloxy
TEV: tobacco etch virus
TMS-ethanol: 2-(trimethylsilyl)ethanol
