# Supplementary material for: Amide-to-Ester Substitution as a Strategy for Optimizing PROTAC Permeability and Cellular Activity
Source: J Med Chem. 2021 Dec 9;64(24):18082–101. doi: 10.1021/acs.jmedchem.1c01496 (PMC8713283; doi:10.1021/acs.jmedchem.1c01496)
Supplement: Supplementary file 1 — jm1c01496_si_001.pdf [file jm1c01496_si_001.pdf]

Supporting information for:

Amide-to-ester substitution as a strategy for optimizing PROTAC permeability and cellular activity

Victoria G Klein,[1#] Adam G. Bond,[2#] Conner Craigon,[2] R. Scott Lokey, [1*] Alessio Ciulli,[2*]

Author Affiliations:
[1] Department of Chemistry and Biochemistry, University of California Santa Cruz, Santa Cruz, CA, USA, 95064
[2] Division of Biological Chemistry and Drug Discovery, School of Life Sciences, University of Dundee, Dow Street, Dundee, DD1 5EH, Scotland, UK.

#co-first authors
*co-corresponding authors: a.ciulli@dundee.ac.uk and slokey@ucsc.edu

List of Tables:

Supplementary Table 1: Physicochemical properties of model compounds……………………..….S3
Supplementary Table 2: MDCK-MDR1 bidirectional permeability……………………………..….S4
Supplementary Table 3: Physicochemical properties of PROTAC compounds…..……………..……..S5
Supplementary Table 4: FP binding data for PROTACs **21** – **28**……………………….……………..….S6
Supplementary Table 5: PROTAC cellular activity………………………………………………...S8

List of Figures:

Supplementary Figure 1: Plasma stability…………………………………………………….....S9
Supplementary Figure 2: Fluorescence polarization (FP) on linker scan model compounds……………………………………………………………….……………….…….S13
Supplementary Figure 3: Protein degradation profiles of PROTACs……………………..…….S14
Supplementary Figure 4: Original Uncropped Western blots………………………..……….S18

Compound Purity: LC-MS Traces……………………………………….....S20

LC/MS spectra for compound **1**………………………………….……..…..…S20
LC/MS spectra for compound **2**…………………………………...……..…S21
LC/MS spectra for compound **3**………………………………..……………...…S22
LC/MS spectra for compound **4**………………………………….…………..…S23
LC/MS spectra for compound **5**…………………………………..……………..…S24
LC/MS spectra for compound **6**…………………………………..………………..…S25
LC/MS spectra for compound **7**…………………………………..……………..…S26
LC/MS spectra for compound **8**………………………………………...……..…S27
LC/MS spectra for compound **9**…………………………………..…………………..…S28
LC/MS spectra for compound **10**………………………………………………..…S29
LC/MS spectra for compound **11**……………………………………………..…S30
LC/MS spectra for compound **12**…………………………………………….…..…S31
LC/MS spectra for compound **13**………………………………………………..…S32
LC/MS spectra for compound **14**……………………………………….…..…S33
LC/MS spectra for compound **15**…………………………………………….…..…S34
LC/MS spectra for compound **16**…………………………………………….…..…S35
LC/MS spectra for compound **17**…………………………………………….…..…S36
LC/MS spectra for compound **18**……………………………………………...S37
LC/MS spectra for compound **19**……………………………………………..…S38
LC/MS spectra for compound **20** ………………………...……………….…..…S39
LC/MS spectra for compound **23**………………………………………………......S40
LC/MS spectra for compound **24**………………………………………..……S41
LC/MS spectra for compound **25**……………………………………..……S42
LC/MS spectra for compound **26**……………………………………..……S43
LC/MS spectra for compound **27**……………………………………….…..…S44
LC/MS spectra for compound **28**……………………………………….…..…S45

Liposcan Selected Ion Chromatograms for Compounds 1 – 20……………S46

Table S-1: Physicochemical properties of model compounds. Green heat map shows calculated lipophilicity from high ALogP (green) to low ALogP (white). Magenta heat map shows PAMPA permeabilities from high (magenta) to low (white). Orange heat map shows experimental $LogD_{(dec/w)}$ from high (orange) to low (white). Blue heat map shows calculated LPE from high (blue) to low (white).

		Cmpd[a]	MW[b]	ALogP	# of HBDs[c]	# of HBAs[d]	PAMPA[e]	LogD (dec/w)[f]	LPE[g]
Liposcan	Amide	VK-P01 (**1**)	616	1.2	4	7	0.07 ± 0.007	-2.2	2.0
		VK-P02 (**2**)	614	2.6	4	6	0.1 ± 0.01	-2.2	0.5
		VK-P03 (**3**)	662	3.2	4	6	0.8 ± 0.04	-1.5	0.6
		VK-P04 (**4**)	654	3.6	4	6	1.1 ± 0.1	-1.4	0.3
		VK-P05 (**5**)	720	4.1	4	6	6.8 ± 0.8	-0.2	1.0
		VK-P06 (**6**)	752	4.9	4	6	3.6 ± 1.0	-0.3	-0.1
		VK-P07 (**7**)	796	6.0	4	7	BLQ	1.0	0.1
	Ester	VK-P08 (**8**)	617	1.9	3	8	0.7 ± 0.1	-1.7	1.8
		VK-P09 (**9**)	615	3.2	3	7	6.5 ± 1.5	-0.5	1.6
		VK-P10 (**10**)	663	3.8	3	7	6.5 ± 0.8	0.8	2.3
		VK-P11 (**11**)	655	4.3	3	7	4.4 ± 2.0	1.1	2.1
		VK-P12 (**12**)	721	4.7	3	7	0.2 ± 0.1	2.4	2.8
		VK-P13 (**13**)	753	5.6	3	7	0.6 ± 0.2	1.9	1.5
		VK-P14 (**14**)	797	6.6	3	8	BLQ	BLD	--
Linkers	Amide	VK-P15 (**15**)	664	1.8	4	7	0.3 ± 0.06	-1.4	2.2
		VK-P16 (**16**)	708	1.7	4	8	0.2 ± 0.01	-2.3	1.4
		VK-P17 (**17**)	752	1.6	4	9	BLQ	-1.9	2.0
	Ester	VK-P18 (**18**)	665	2.5	3	8	5.8 ± 0.8	-0.2	2.7
		VK-P19 (**19**)	709	2.3	3	9	3.2 ± 0.2	-0.5	2.5
		VK-P20 (**20**)	753	2.2	3	10	1.9 ± 0.3	-0.7	2.4

a: Cmpd: compound
b: MW: molecular weight
c: HBD: hydrogen bond donor
d: HBA: hydrogen bond acceptor
e: Pe (x 10^{-6} cm/s)
f: partition coefficient of decadiene/water
g: $LPE = LogD_{(dec/w)} - 1.06(ALogP) + 5.47$

Table S-2: MDCK-MDR1 bidirectional permeability for model compounds and two PROTACS. Digoxin and propranolol were used as controls for low and high cell permeability, respectively. N = 2. A-B: apical to basal; B-A: basal to apical. Efflux ratio calculated as B-A/A-B.

Compound	Concentration [µM]	Papp, A-B (x10^{-6} cm/s)		Papp, B-A (x10^{-6} cm/s)		Ratio B-A/A-B	Recover Rate (%)
		Value	Mean	Value	Mean		
Digoxin	5	0.9<br>0.7	0.8	20.2<br>22.7	21.5	26.3	93
Propranolol	5	51.1<br>47.2	49.1	41.6<br>41.5	41.6	0.8	87
VK-P01 (**1**)	5	0.6<br>0.5	0.5	1.5<br>1.8	1.7	3.1	108
VK-P02 (**2**)	5	0.4<br>0.5	0.4	2.8<br>3.1	2.9	6.8	100
VK-P03 (**3**)	5	1.1<br>0.3	0.7	7.9<br>8.9	8.4	11.9	100
VK-P04 (**4**)	5	0.3<br>0.3	0.3	10.5<br>10.1	10.3	33.3	98
VK-P05 (**5**)	5	0.1<br>0.2	0.2	22.9<br>24.6	23.7	157.5	85
VK-P06 (**6**)	5	0.2<br>0.1	0.1	20.4<br>16.6	18.5	133.1	86
VK-P07 (**7**)	5	BLD<br>BLD	N/A	0.2<br>0.2	0.2	N/A	31
VK-P08 (**8**)	5	0.5<br>0.4	0.4	11.0<br>10.9	11.0	25.8	109
VK-P09 (**9**)	5	1.0<br>0.7	0.9	45.3<br>48.8	47.0	54.6	113
VK-P10 (**10**)	5	0.7<br>1.1	0.9	42.8<br>41.6	42.2	49.4	81
VK-P11 (**11**)	5	0.3<br>0.3	0.3	36.2<br>35.0	35.6	142.1	78
VK-P12 (**12**)	5	BLD<br>BLD	N/A	4.5<br>4.5	4.5	N/A	29
VK-P13 (**13**)	5	0.2<br>0.2	0.2	13.8<br>14.1	13.9	84.0	41
VK-P14 (**14**)	5	BLD<br>BLD	N/A	BLD<br>BLD	N/A	N/A	15
VK-P15 (**15**)	5	0.3<br>0.4	0.4	4.5<br>4.6	4.6	12.7	112
VK-P16 (**16**)	5	BLD<br>BLD	N/A	3.1<br>3.5	3.3	N/A	115
VK-P17 (**17**)	5	BLD<br>BLD	N/A	1.7<br>2.2	2.0	N/A	112
VK-P18 (**18**)	5	0.6<br>0.5	0.6	36.4<br>41.8	39.1	70.7	109
VK-P19 (**19**)	5	BLD<br>BLD	N/A	22.4<br>25.1	23.7	N/A	102
VK-P20 (**20**)	5	1.0<br>1.0	1.0	18.0<br>18.7	18.3	17.8	111
MZ1 (**21**)	5	BLD<br>BLD	N/A	1.6<br>1.5	1.5	N/A	98
OMZ1 (**25**)	5	BLD<br>BLD	N/A	BLD<br>BLD	N/A	N/A	43

Table S-3: Physicochemical properties of PROTAC compounds. Green heat map shows calculated lipophilicity from high ALogP (green) to low ALogP (white). Magenta heat map shows PAMPA permeabilities from high (magenta) to low (white).

		Cmpd[a]	MW[b]	ALogP	# of HBDs[c]	# of HBAs[d]	PAMPA[e]
PROTAC	Amide	MZ1 (**21**)	1003	3.6	4	12	0.01 ± 0.0002
		ARV-771 (**22**)	987	4.2	4	11	0.2 ± 0.06
		AB1 (**23**)	1001	4.8	4	11	0.5 ± 0.07
		AB2 (**24**)	973	3.8	4	11	0.08 ± 0.02
	Ester	OMZ1 (**25**)	1004	4.3	3	13	0.1 ± 0.02
		OARV-771 (**26**)	988	4.8	3	12	0.3 ± 0.1
		OAB1 (**27**)	1002	5.5	3	12	0.2 ± 0.03
		OAB2 (**28**)	974	4.4	3	12	0.6 ± 0.06

a: Cmpd: compound
b: MW: molecular weight
c: HBD: hydrogen bond donor
d: HBA: hydrogen bond acceptor
e: Pe (x 10^{-6} cm/s)

Table S-4: FP binding data for PROTACs **21** – **28** to VBC ± BET bromodomain

PROTAC		+ target	FP Competition (VHL) K_d (nM)	± SEM	N	α	pKd	± SEM	ΔpK_d	± error
MZ1	*binary*	-	**81**	11	5	-	**7.09**	0.06	-	-
(**21**)	*ternary*	$Brd2^{BD1}$	37	0.7		2.2	7.44	0.01	0.34	0.06
		$Brd2^{BD2}$	2.8	0.05	2	29	8.55	0.01	1.46	0.06
		$Brd3^{BD1}$	28	4	2	2.8	7.55	0.07	0.45	0.09
		$Brd3^{BD2}$	15	0.2	2	5.3	7.82	0.01	0.73	0.06
		$Brd4^{BD1}$	19	3	2	4.2	7.71	0.06	0.62	0.09
		$Brd4^{BD2}$	1.5	0.5	3	54	8.82	0.13	1.73	0.15
ARV-771	*binary*	-	**34**	5	6	-	**7.47**	0.08	-	-
(**22**)	*ternary*	$Brd2^{BD1}$	14	0.2	2	2.4	7.85	0.01	0.38	0.08
		$Brd2^{BD2}$	7.3	0.4	2	4.6	8.13	0.02	0.66	0.09
		$Brd3^{BD1}$	17	3.7	2	2.0	7.77	0.10	0.30	0.13
		$Brd3^{BD2}$	7.8	0.5	2	4.3	8.11	0.03	0.64	0.09
		$Brd4^{BD1}$	13	2	2	2.6	7.89	0.07	0.42	0.11
		$Brd4^{BD2}$	2.5	1	3	13	8.60	0.19	1.12	0.21
AB1	*binary*	-	**98**	14	6	-	**7.01**	0.06	-	-
(**23**)	*ternary*	$Brd2^{BD1}$	66	1	2	1.5	7.18	0.01	0.17	0.06
		$Brd2^{BD2}$	9.2	0.5	2	11	8.04	0.02	1.03	0.06
		$Brd3^{BD1}$	57	11	2	1.7	7.25	0.08	0.24	0.10
		$Brd3^{BD2}$	27	2	2	3.6	7.56	0.03	0.56	0.07
		$Brd4^{BD1}$	42	5	2	2.3	7.37	0.05	0.37	0.08
		$Brd4^{BD2}$	3.7	1	3	27	8.44	0.14	1.43	0.15
AB2	*binary*	-	**66**	7	6	-	**7.18**	0.05	-	-
(**24**)	*ternary*	$Brd2^{BD1}$	29	2	2	2.3	7.54	0.01	0.36	0.05
		$Brd2^{BD2}$	9.7	0.7	2	6.8	8.01	0.03	0.83	0.06
		$Brd3^{BD1}$	23	4	2	2.9	7.65	0.09	0.46	0.10
		$Brd3^{BD2}$	20	2	2	3.3	7.70	0.05	0.52	0.07
		$Brd4^{BD1}$	18	0.6	2	3.7	7.74	0.01	0.56	0.05
		$Brd4^{BD2}$	3.0	1	3	22	8.52	0.13	1.34	0.14
OMZ1	*binary*	-	**248**	30	6	-	**6.61**	0.05	-	-
(**25**)	*ternary*	$Brd2^{BD1}$	129	8	2	1.9	6.89	0.03	0.29	0.06
		$Brd2^{BD2}$	15	1	2	17	7.84	0.03	1.23	0.06
		$Brd3^{BD1}$	112	21	2	2.2	6.95	0.08	0.35	0.10
		$Brd3^{BD2}$	47	2	2	5.3	7.33	0.01	0.72	0.06
		$Brd4^{BD1}$	93	15	2	2.7	7.03	0.07	0.43	0.09

		$Brd4^{BD2}$	6.4	3	3	39	8.19	0.19	1.59	0.19
OARV-771	*binary*	-	**63**	11	6	-	**7.20**	0.08	-	-
(**26**)	*ternary*	$Brd2^{BD1}$	32	3	2	2.0	7.50	0.04	0.29	0.09
		$Brd2^{BD2}$	14	0.1	2	4.5	7.86	0.00	0.65	0.08
		$Brd3^{BD1}$	42	6	2	1.5	7.38	0.07	0.18	0.11
		$Brd3^{BD2}$	20	3	2	3.1	7.70	0.07	0.50	0.11
		$Brd4^{BD1}$	33	5	2	1.9	7.48	0.06	0.28	0.10
		$Brd4^{BD2}$	7.3	3	3	8.5	8.13	0.18	0.93	0.20
OAB1	*binary*	-	**236**	19	6	-	**6.63**	0.04	-	-
(**27**)	*ternary*	$Brd2^{BD1}$	161	6	2	1.5	6.79	0.01	0.16	0.04
		$Brd2^{BD2}$	42	1	2	5.6	7.38	0.01	0.75	0.04
		$Brd3^{BD1}$	165	29	2	1.4	6.78	0.08	0.15	0.08
		$Brd3^{BD2}$	74	0.2	2	3.2	7.13	0.00	0.50	0.04
		$Brd4^{BD1}$	128	17	2	1.8	6.89	0.06	0.26	0.07
		$Brd4^{BD2}$	18	6	3	13	7.74	0.13	1.12	0.14
OAB2	*binary*	-	**143**	7	6	-	**6.84**	0.06	-	-
(**28**)	*ternary*	$Brd2^{BD1}$	54	5	2	2.7	7.27	0.04	0.42	0.07
		$Brd2^{BD2}$	21	0.5	2	6.8	7.67	0.01	0.83	0.06
		$Brd3^{BD1}$	61	13	2	2.3	7.21	0.09	0.37	0.11
		$Brd3^{BD2}$	45	11	2	3.2	7.34	0.10	0.50	0.12
		$Brd4^{BD1}$	40	5	2	3.6	7.40	0.05	0.55	0.08
		$Brd4^{BD2}$	21	7	3	7.0	7.69	0.14	0.84	0.15

Tabulated FP data giving derived dissociative constants (K_d) ± S.E.M from 2 – 6 independent repeats (N) for both binary and ternary binding between VHL, PROTAC ± BET BD. Cooperativity (α) = K_d^{binary} / $K_d^{ternary}$. ΔpK_d = $Log_{10}(\alpha)$ = $pK_d^{ternary}$ - pK_d^{binary}. ΔpK_d error is propagated from SQRT(($pK_d^{ternary}$ S.E.M)^2 + (pK_d^{binary} S.E.M)^2). Red heat map used to show decreasing affinity (increasing K_d, decreasing pK_d) white to red. Green heat map used to show increasing cooperativity (increasing α, increasing ΔpK_d) white to green.

Table S-5: PROTAC cellular activity.

	Protein Degradation			Cell Viability	
	Brd4 Long	Brd3	Brd2	MV4;11	22Rv1
Compound	pDC_{50}[a]	pDC_{50}[a]	pDC_{50}[a]	pEC_{50}[a]	pEC_{50}[b]
**21**	7.2 ± 0.2	6.6 ± 0.2	6.6 ± 0.2	6.9 ± 0.1	6.3 ± 0.1
**22**	7.4 ± 0.2	6.7 ± 0.2	6.9 ± 0.2	7.7 ± 0.2	7.4 ± 0.2
**23**	7.5 ± 0.2	7.3 ± 0.1	7.4 ± 0.1	7.0 ± 0.2	6.4 ± 0.1
**24**	8.2 ± 0.2	8.4 ± 0.3	8.9 ± 0.2	6.7 ± 0.1	6.2 ± 0.1
**25**	6.9 ± 0.2	6.8 ± 0.1	6.8 ± 0.1	6.7 ± 0.1	6.1 ± 0.1
**26**	7.2 ± 0.2	7.0 ± 0.1	7.1 ± 0.2	8.4 ± 0.2	7.2 ± 0.2
**27**	6.9 ± 0.1	6.7 ± 0.1	6.6 ± 0.1	6.9 ± 0.1	6.5 ± 0.1
**28**	7.5 ± 0.1	7.2 ± 0.1	7.2 ± 0.2	7.3 ± 0.1	6.6 ± 0.1

[a] calculated as mean (± S.E.) from three independent biological experiments.

[b] calculated as mean (± S.E.) from two independent biological experiments.

Figure S-1: Plasma stability results for model compounds (**1 – 20**) and PROTACs (**21 – 28**)

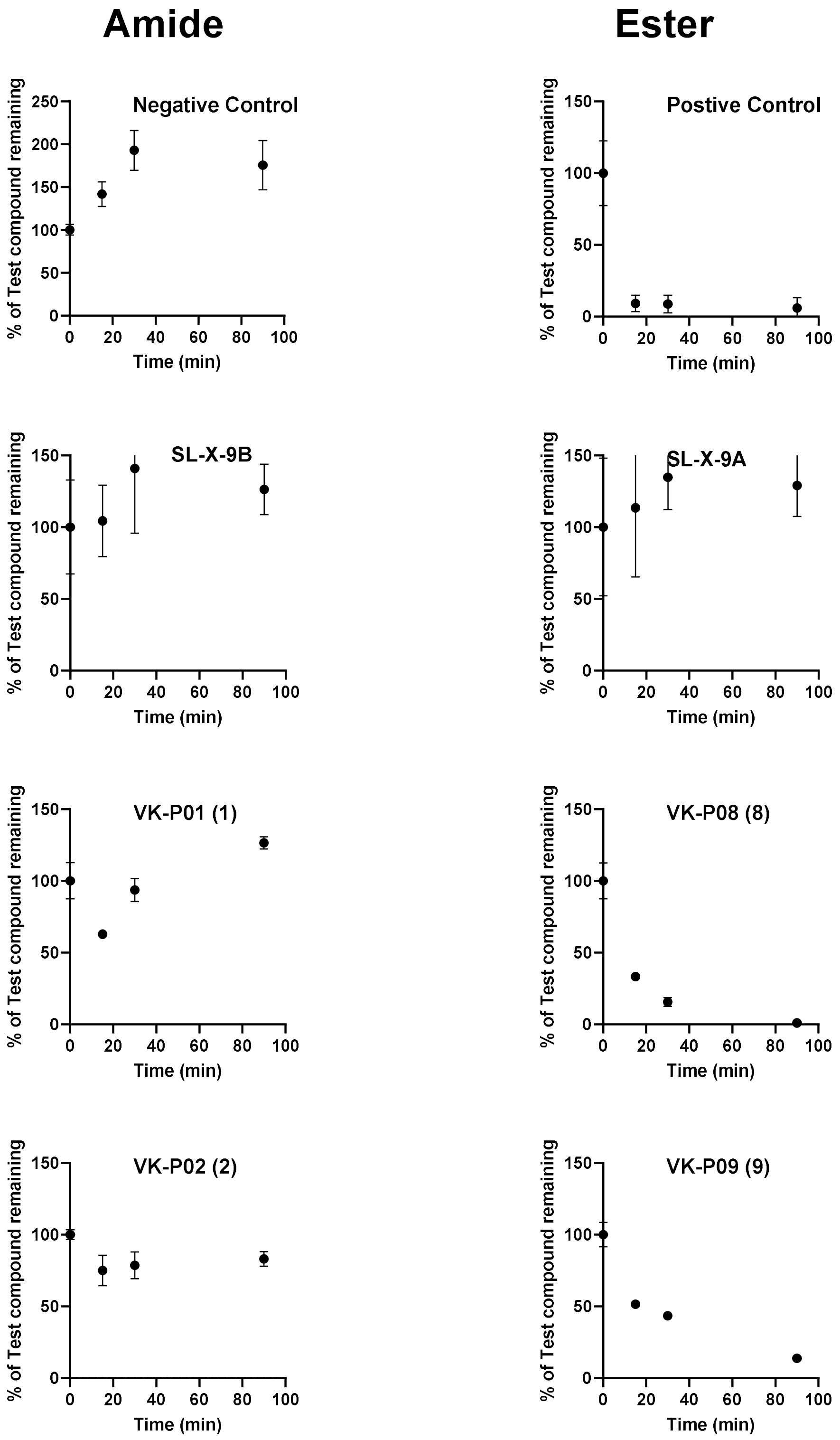

Amide

Ester

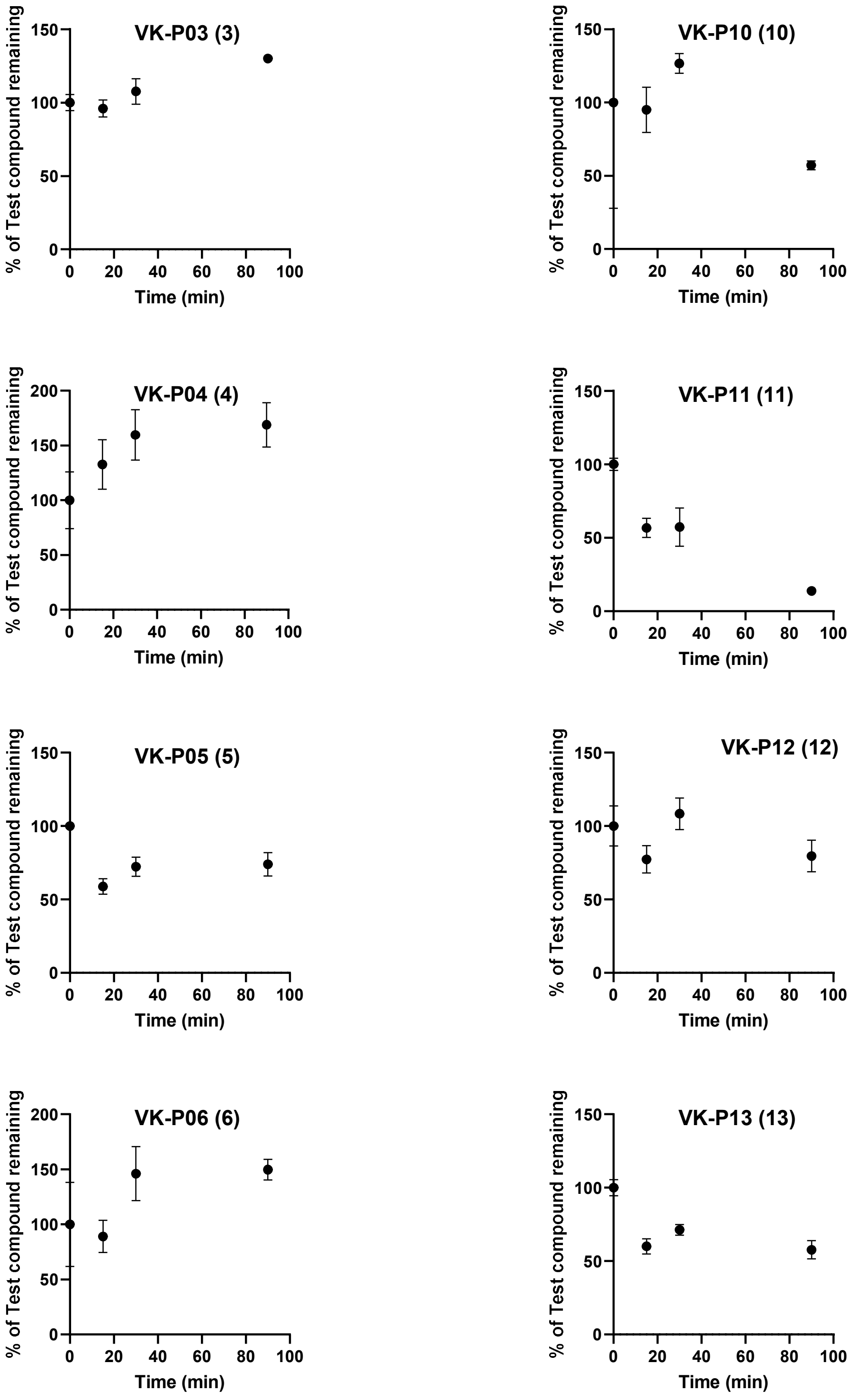

VK-P03 (3)
VK-P04 (4)
VK-P05 (5)
VK-P06 (6)
VK-P10 (10)
VK-P11 (11)
VK-P12 (12)
VK-P13 (13)
% of Test compound remaining
Time (min)

Amide   Ester

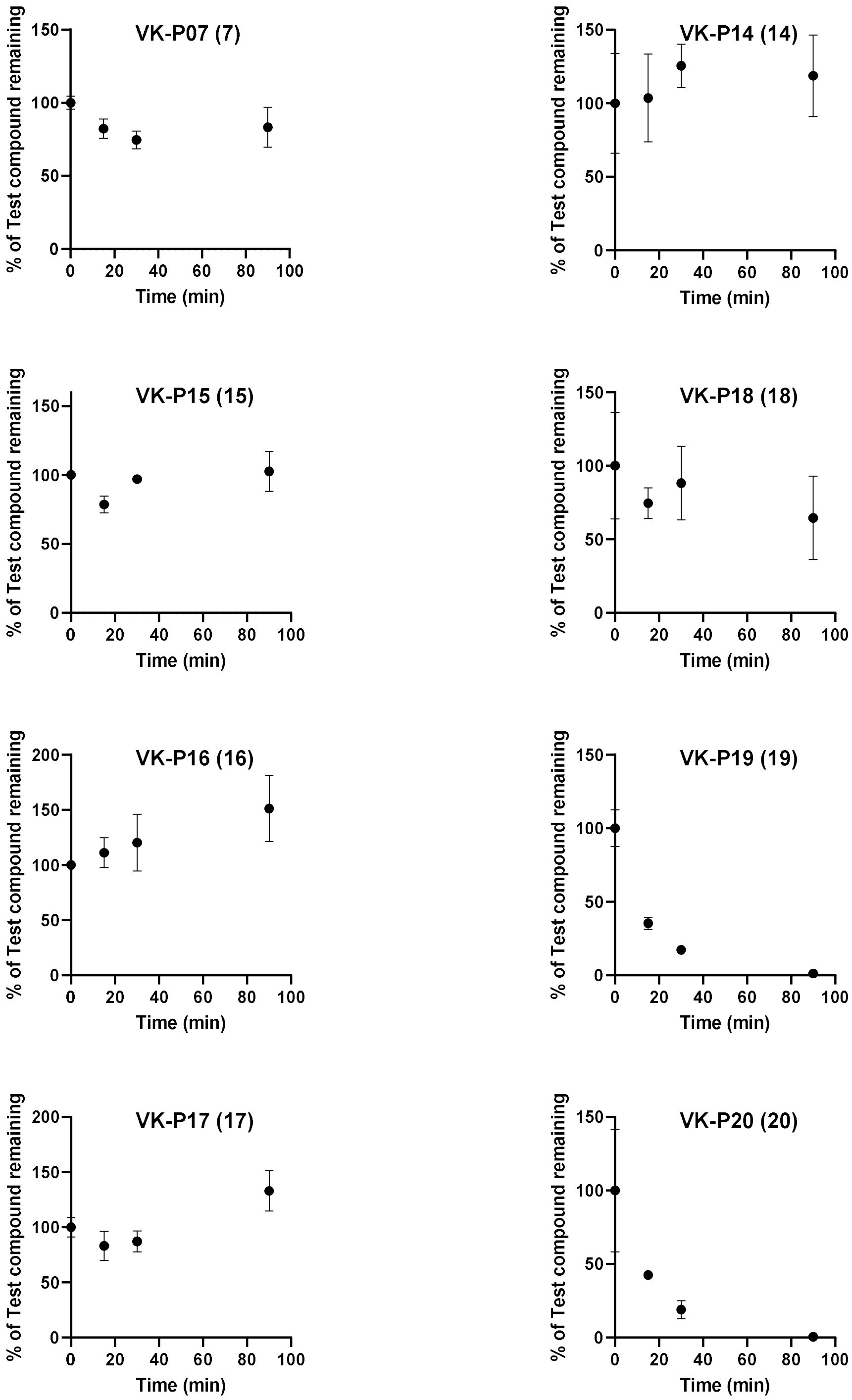
VK-P07 (7)
VK-P14 (14)
VK-P15 (15)
VK-P18 (18)
VK-P16 (16)
VK-P19 (19)
VK-P17 (17)
VK-P20 (20)
% of Test compound remaining
Time (min)
0
20
40
60
80
100
50
150
200

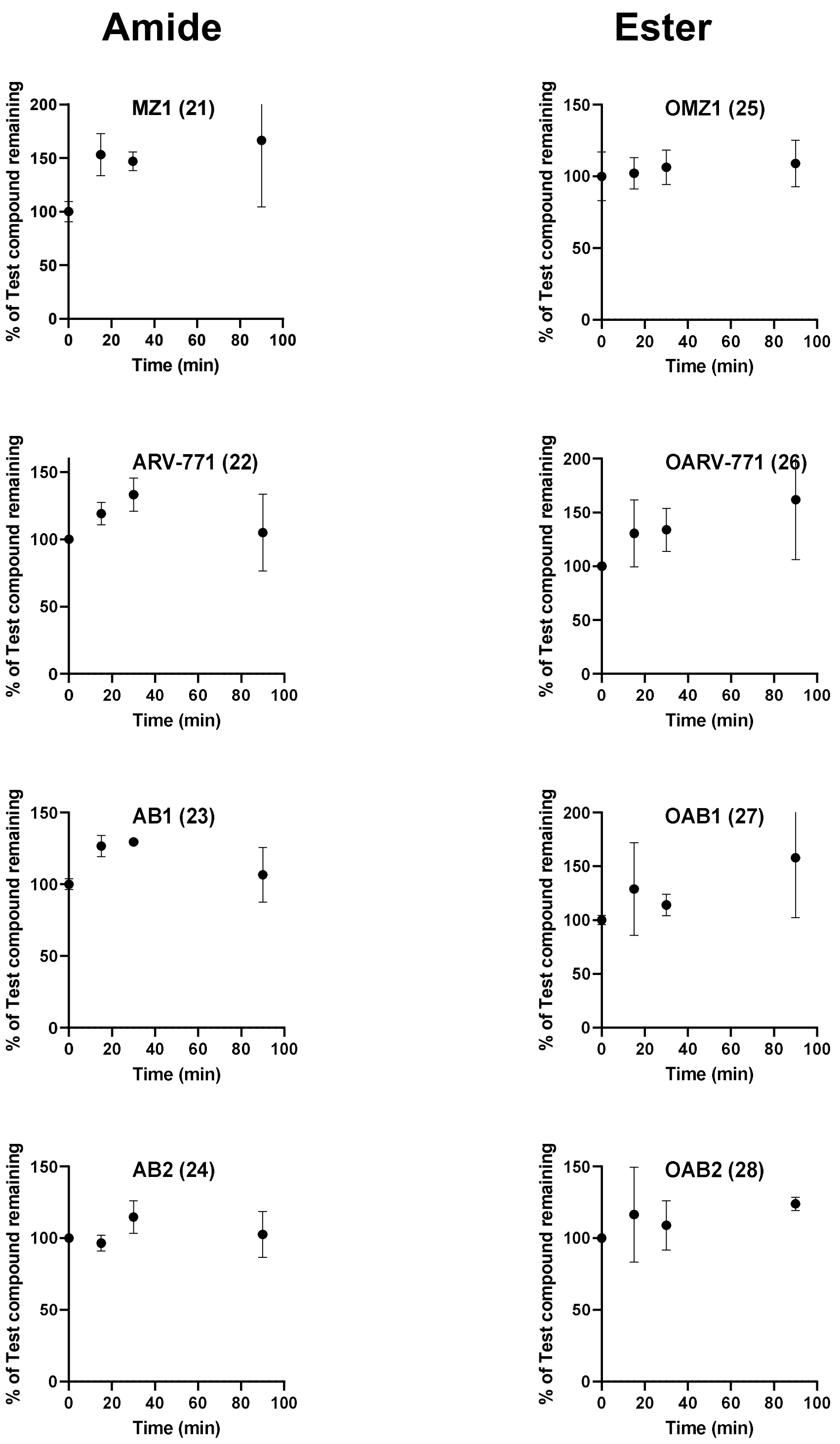

Figure S-1: Human plasma stability results for model compounds (**1 – 20**) and PROTACs (**21 – 28**). Time points collected at 0, 15, 30, and 90 minutes. Normalized to concentration at time zero. See methods for full experimental details. N= 3, error bars: ±SD

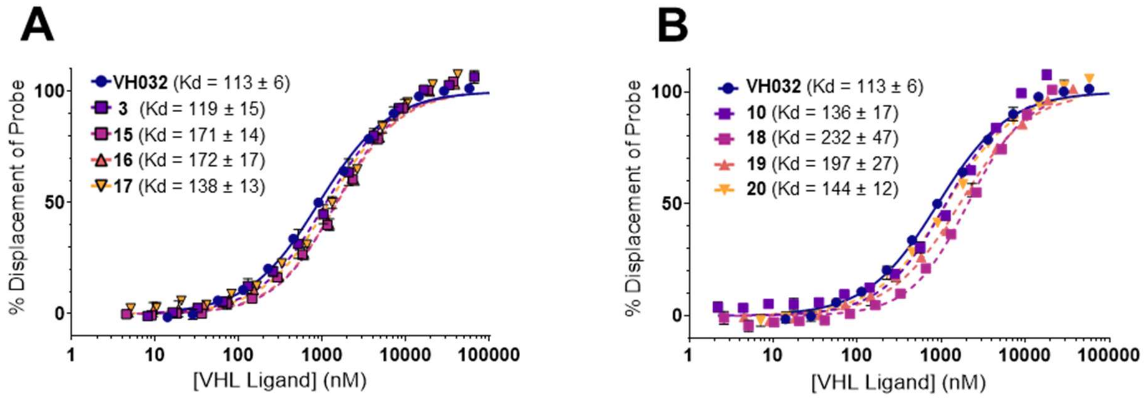

Figure S-2: Fluorescence polarization (FP) on linker scan model compounds Representative fluorescence polarization data for amide (*A*) and ester (*B*) model compounds compared to known VHL-ligand, VH032. Includes linker scan model compounds for alkyl linkers (**3** and **10**) and PEG linkers that are 1-PEG (**15** and **18**), 2-PEG (**16** and **19**), or 3-PEG (**17** and **20**). K_d values are mean ± S.E.M from $N = 3$ for VH032 and amides (**3**, **15** – **17**) and $N = 2$ for esters (**10**, **18** – **20**).

Figure S-3: Protein degradation profiles of PROTACs **21** – **28** for Brd4 long, Brd3 and Brd2. Intensity values were quantified as described in the Methods.

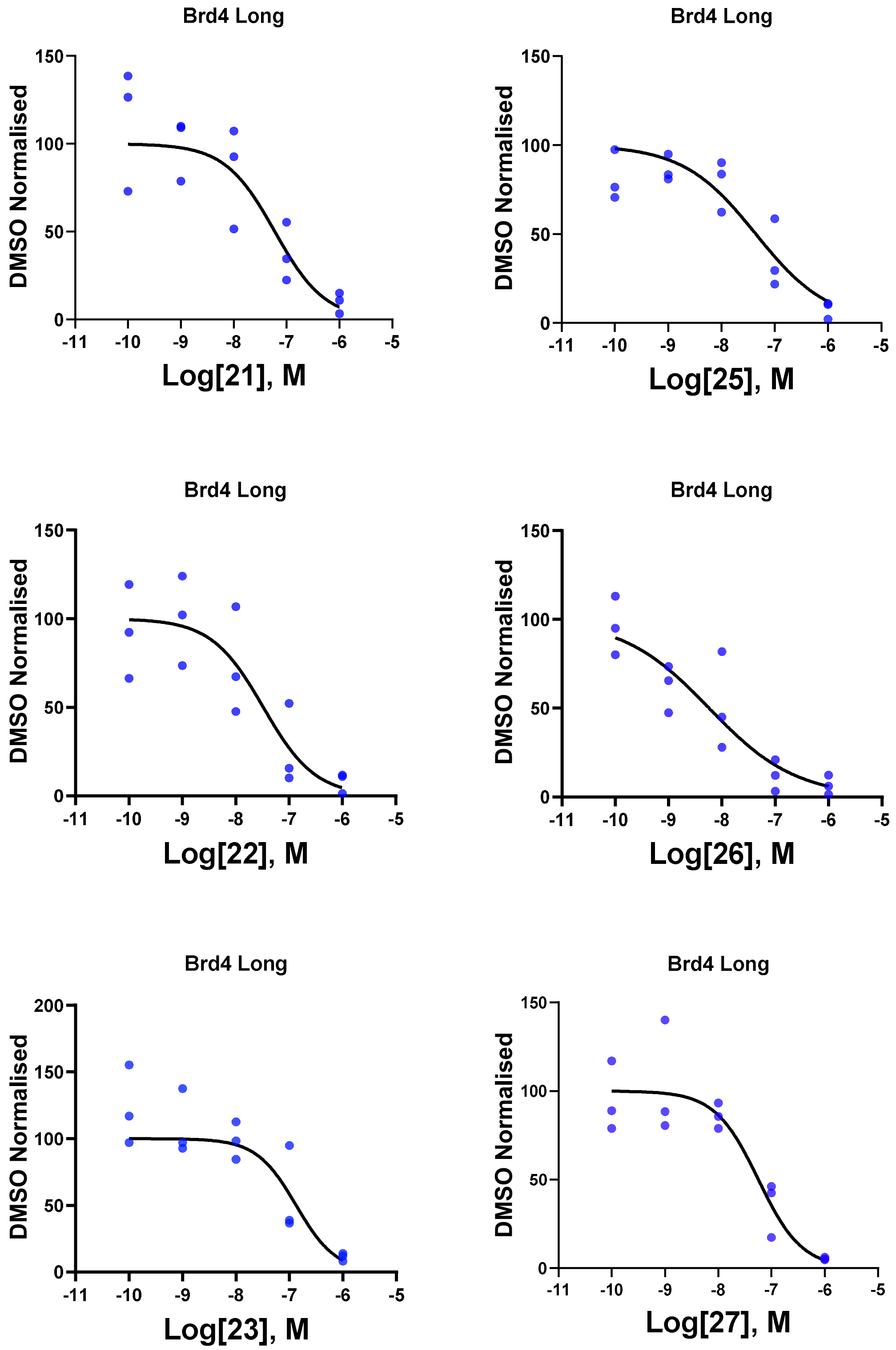

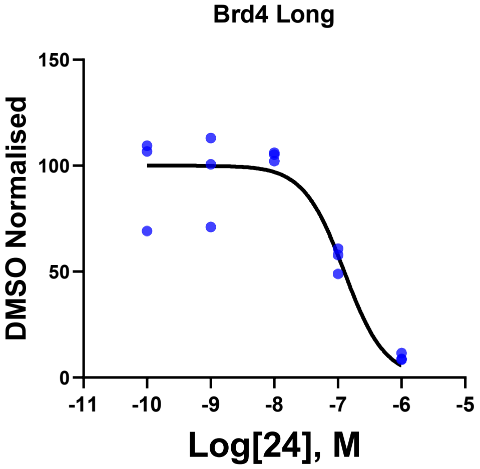
Brd4 Long
DMSO Normalised
150
100
50
0
-11
-10
-9
-8
-7
-6
-5
Log[24], M

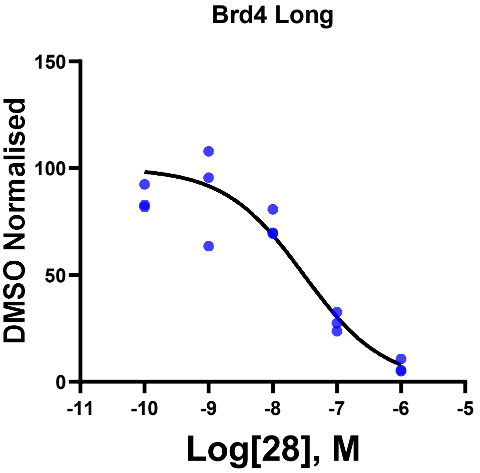
Brd4 Long
DMSO Normalised
150
100
50
0
-11
-10
-9
-8
-7
-6
-5
Log[28], M

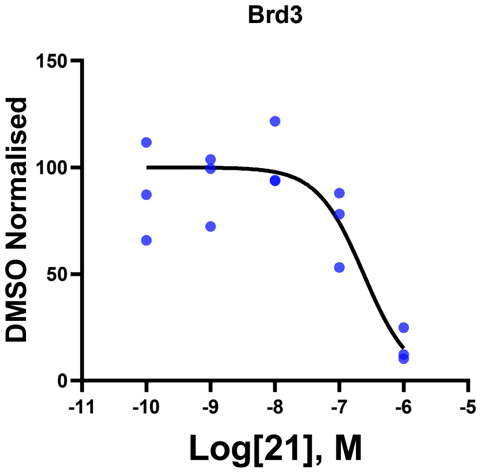
Brd3
DMSO Normalised
150
100
50
0
-11
-10
-9
-8
-7
-6
-5
Log[21], M

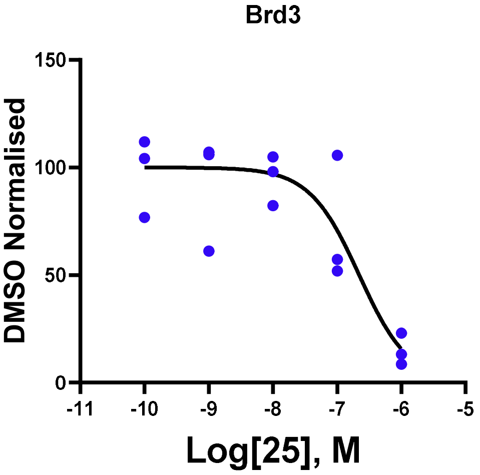
Brd3
DMSO Normalised
150
100
50
0
-11
-10
-9
-8
-7
-6
-5
Log[25], M

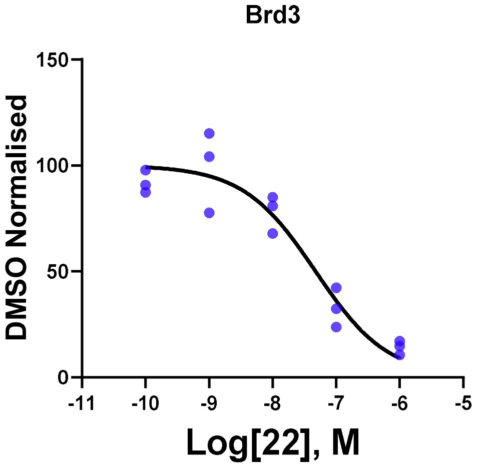
Brd3
DMSO Normalised
150
100
50
0
-11
-10
-9
-8
-7
-6
-5
Log[22], M

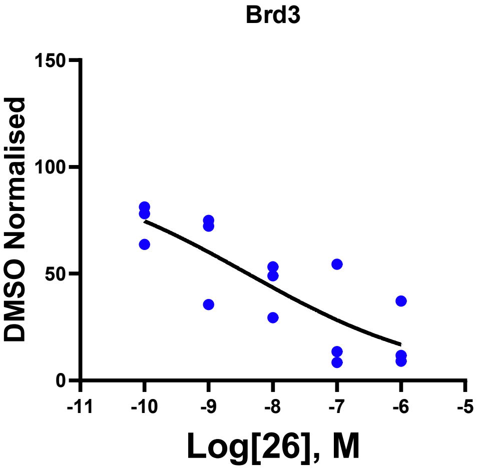
Brd3
DMSO Normalised
150
100
50
0
-11
-10
-9
-8
-7
-6
-5
Log[26], M

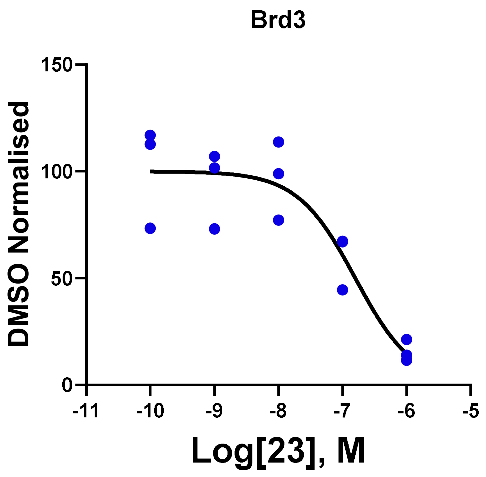
Brd3
DMSO Normalised
150
100
50
0
-11
-10
-9
-8
-7
-6
-5
Log[23], M

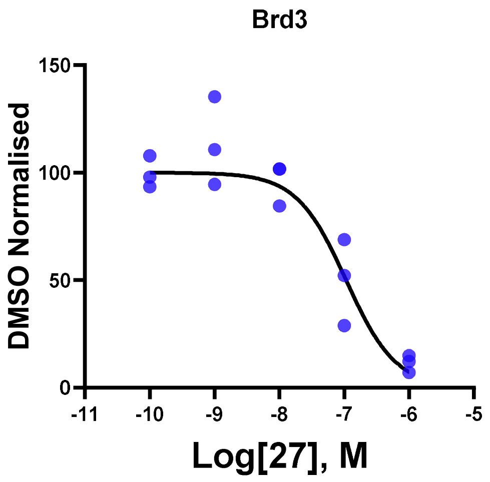
Brd3
DMSO Normalised
150
100
50
0
-11
-10
-9
-8
-7
-6
-5
Log[27], M

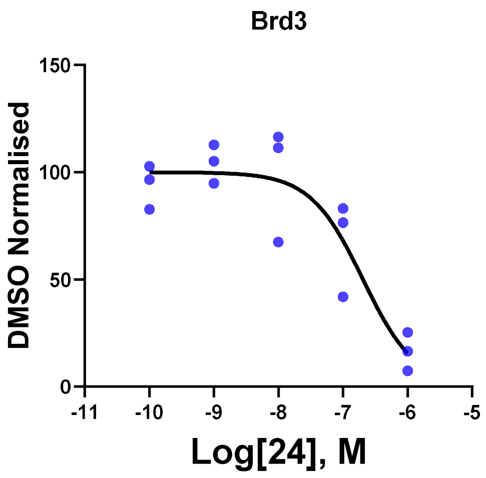
Brd3
DMSO Normalised
150
100
50
0
-11
-10
-9
-8
-7
-6
-5
Log[24], M

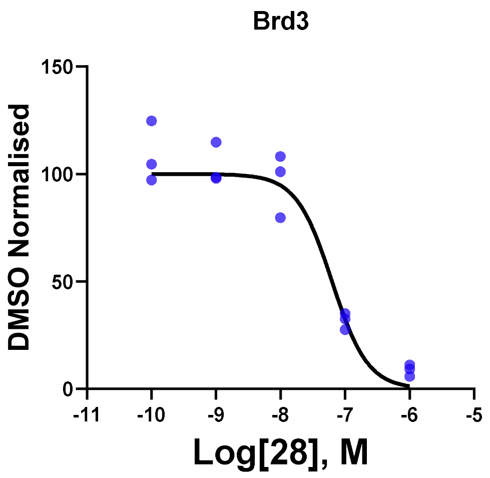
Brd3
DMSO Normalised
150
100
50
0
-11
-10
-9
-8
-7
-6
-5
Log[28], M

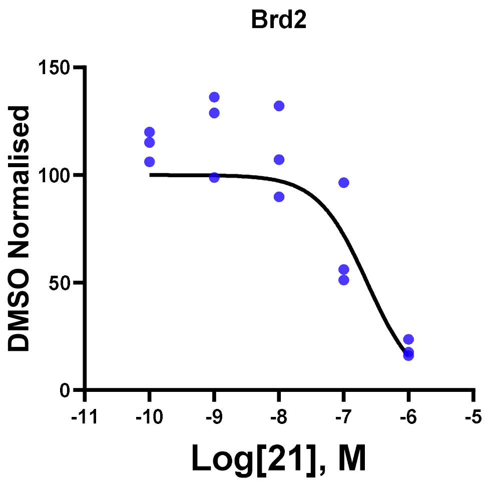
Brd2
DMSO Normalised
150
100
50
0
-11
-10
-9
-8
-7
-6
-5
Log[21], M

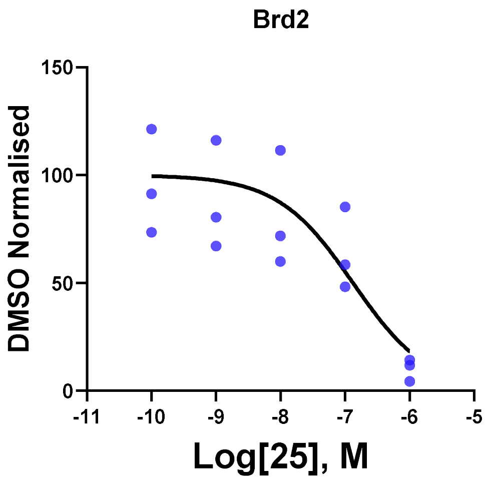
Brd2
DMSO Normalised
150
100
50
0
-11
-10
-9
-8
-7
-6
-5
Log[25], M

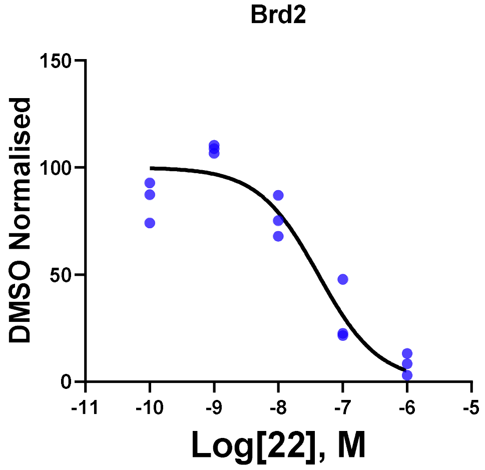
Brd2
DMSO Normalised
150
100
50
0
-11
-10
-9
-8
-7
-6
-5
Log[22], M

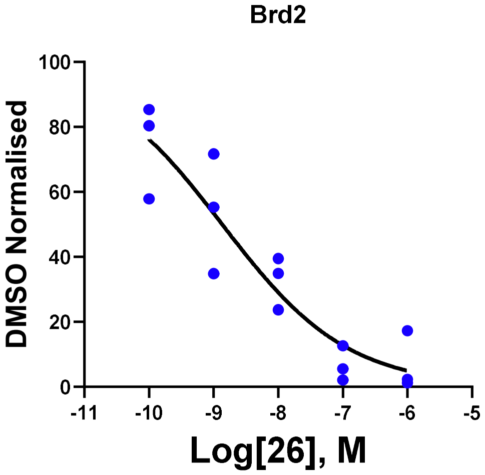
Brd2
DMSO Normalised
100
80
60
40
20
0
-11
-10
-9
-8
-7
-6
-5
Log[26], M

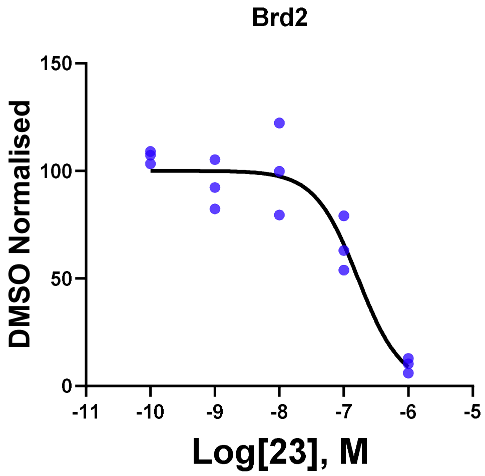
Brd2
DMSO Normalised
150
100
50
0
-11
-10
-9
-8
-7
-6
-5
Log[23], M

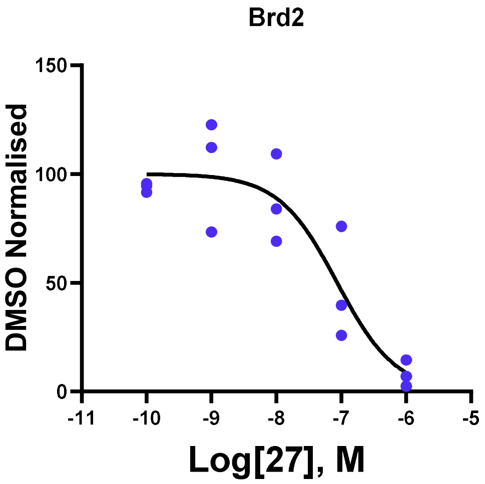
Brd2
DMSO Normalised
150
100
50
0
-11
-10
-9
-8
-7
-6
-5
Log[27], M

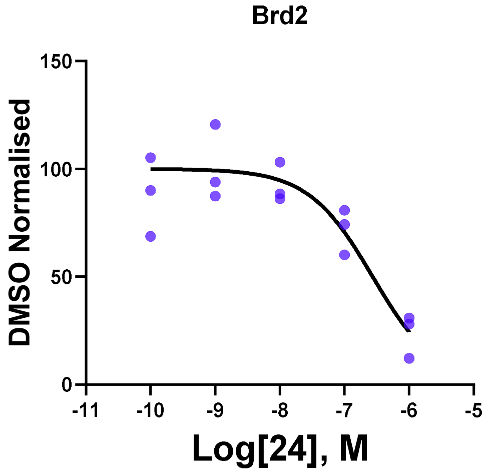
Brd2
DMSO Normalised
150
100
50
0
-11
-10
-9
-8
-7
-6
-5
Log[24], M

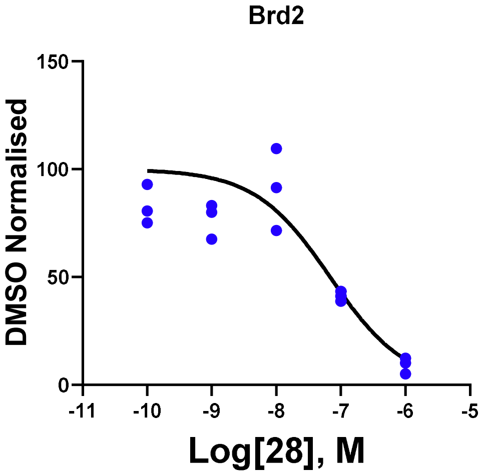
Brd2
DMSO Normalised
150
100
50
0
-11
-10
-9
-8
-7
-6
-5
Log[28], M

Figure S-4: Original Uncropped Western blots for PROTACs **21** – **28**

Dashed boxes mark the cropped area of blots shown in main text figures.

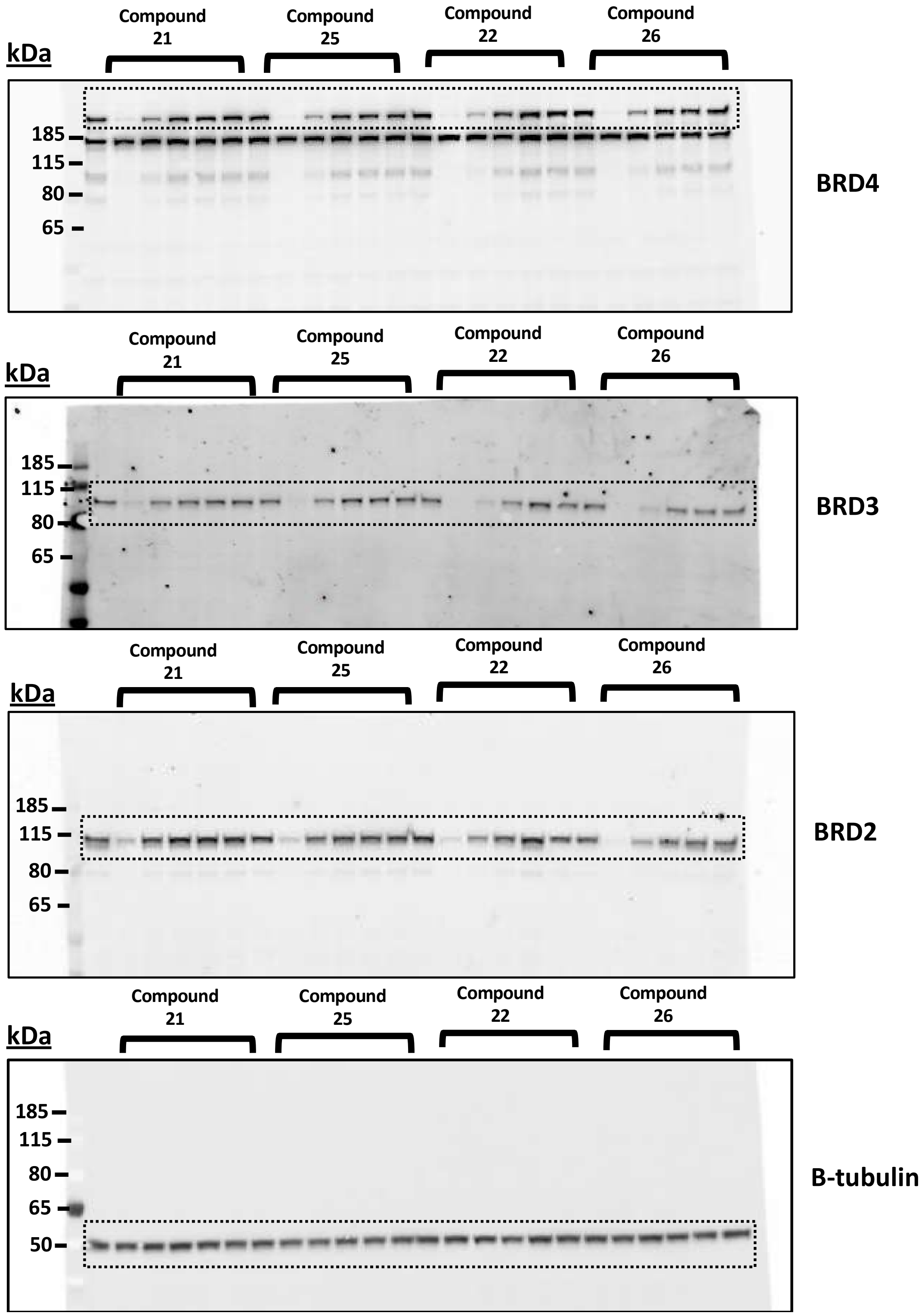

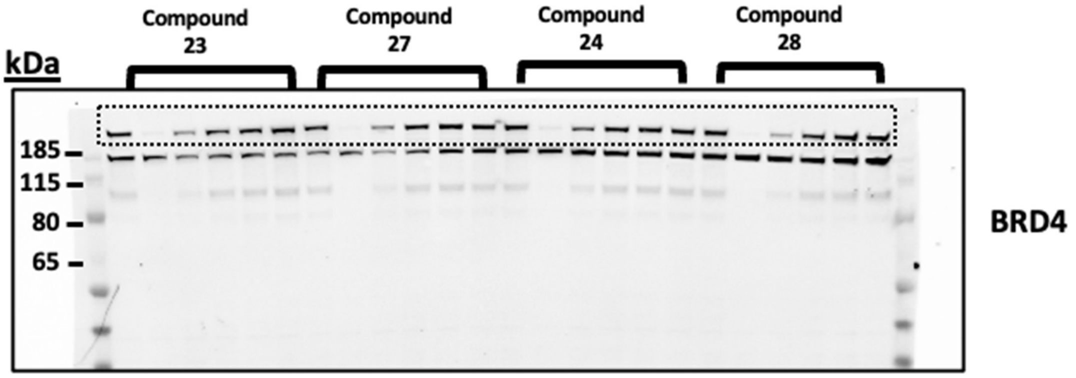
Compound 23
Compound 27
Compound 24
Compound 28
kDa
185
115
80
65
BRD4

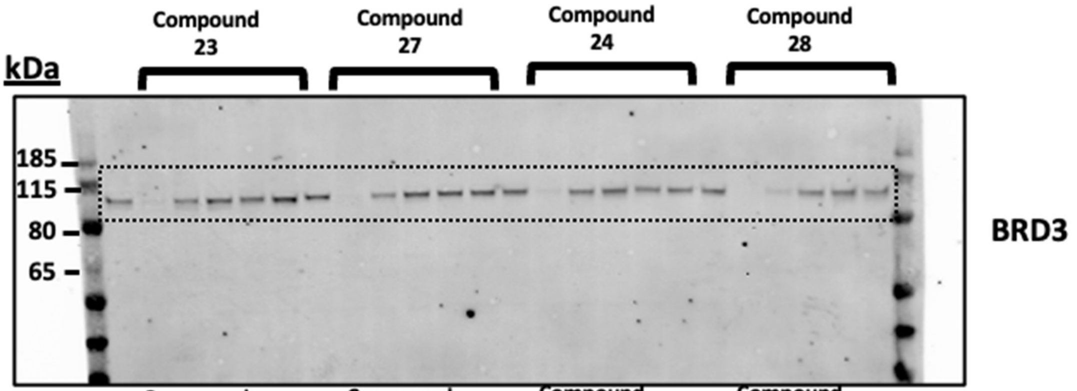
Compound 23
Compound 27
Compound 24
Compound 28
kDa
185
115
80
65
BRD3

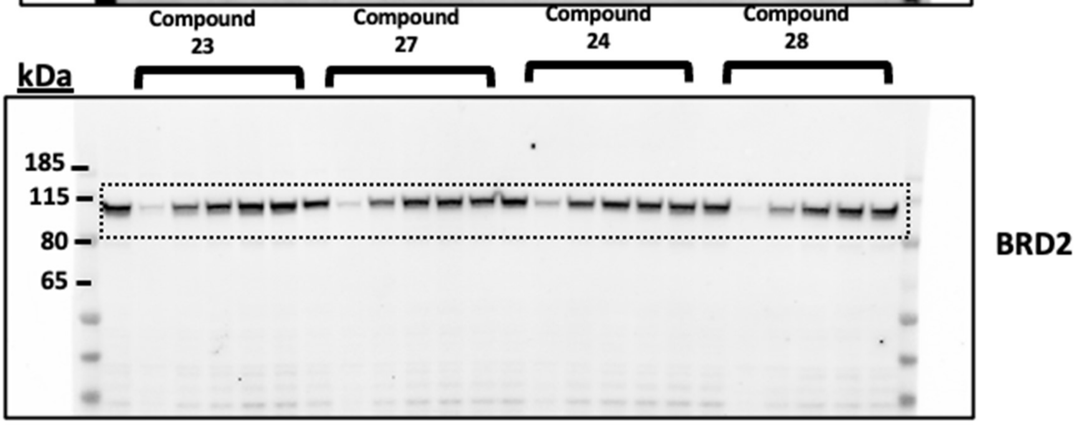
Compound 23
Compound 27
Compound 24
Compound 28
kDa
185
115
80
65
BRD2

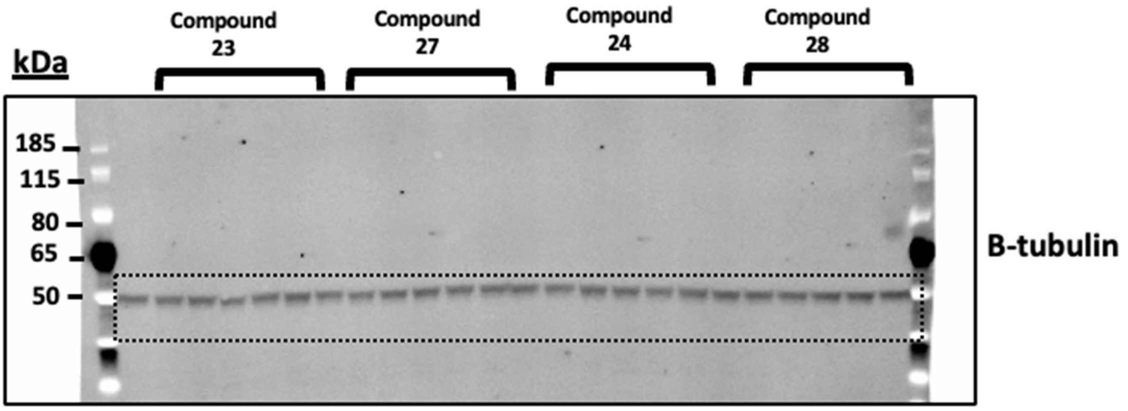
Compound 23
Compound 27
Compound 24
Compound 28
kDa
185
115
80
65
50
B-tubulin

Compound Purity: LC-MS Traces

I. VK – P01 (**1**)

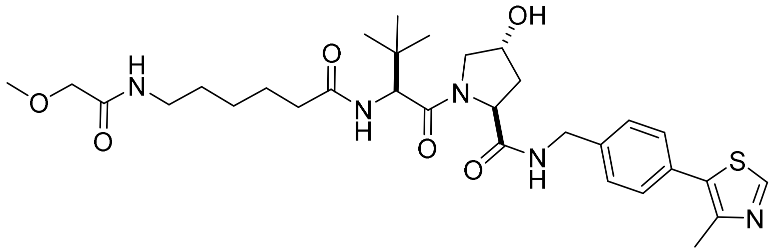

Purity by HPLC: >95%.

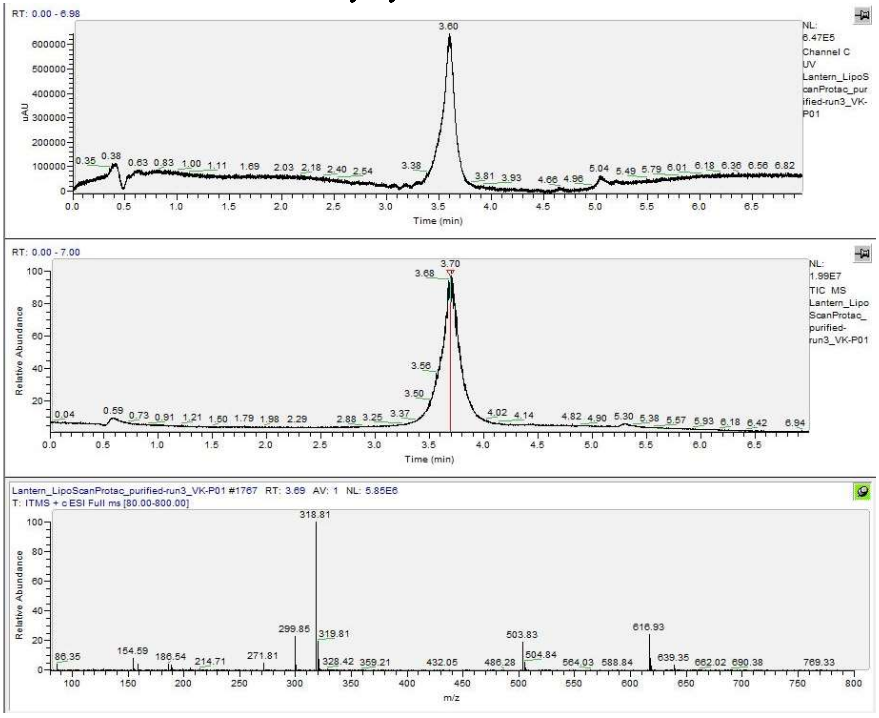

II. VK-P02 (**2**)

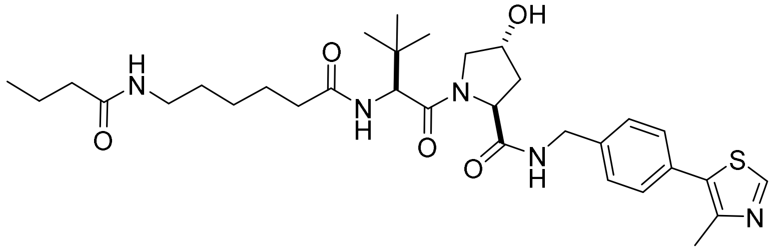

Purity by HPLC: >95%.

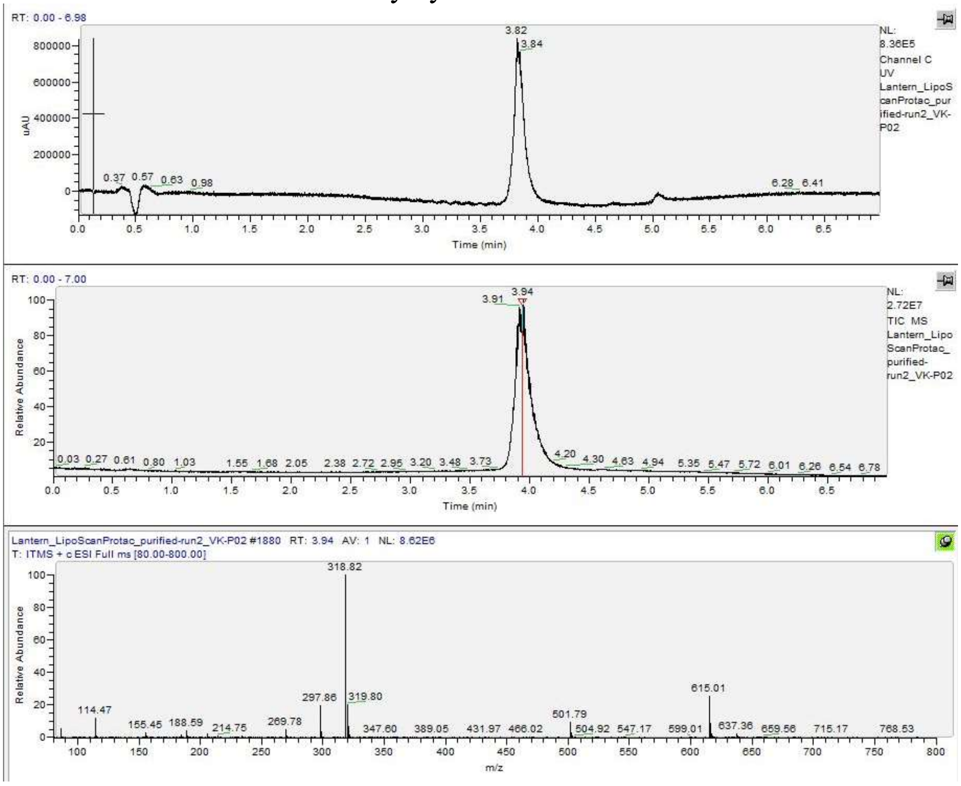

III. VK-P03 (**3**)

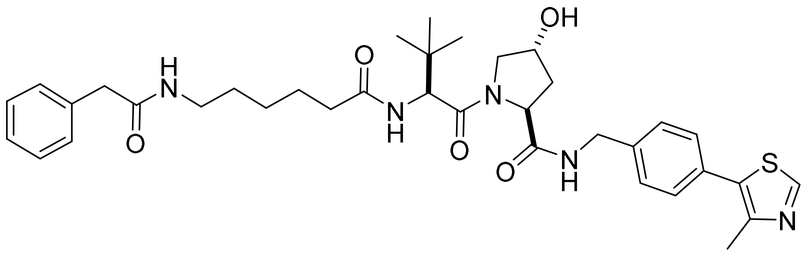

Purity by HPLC: >95%.

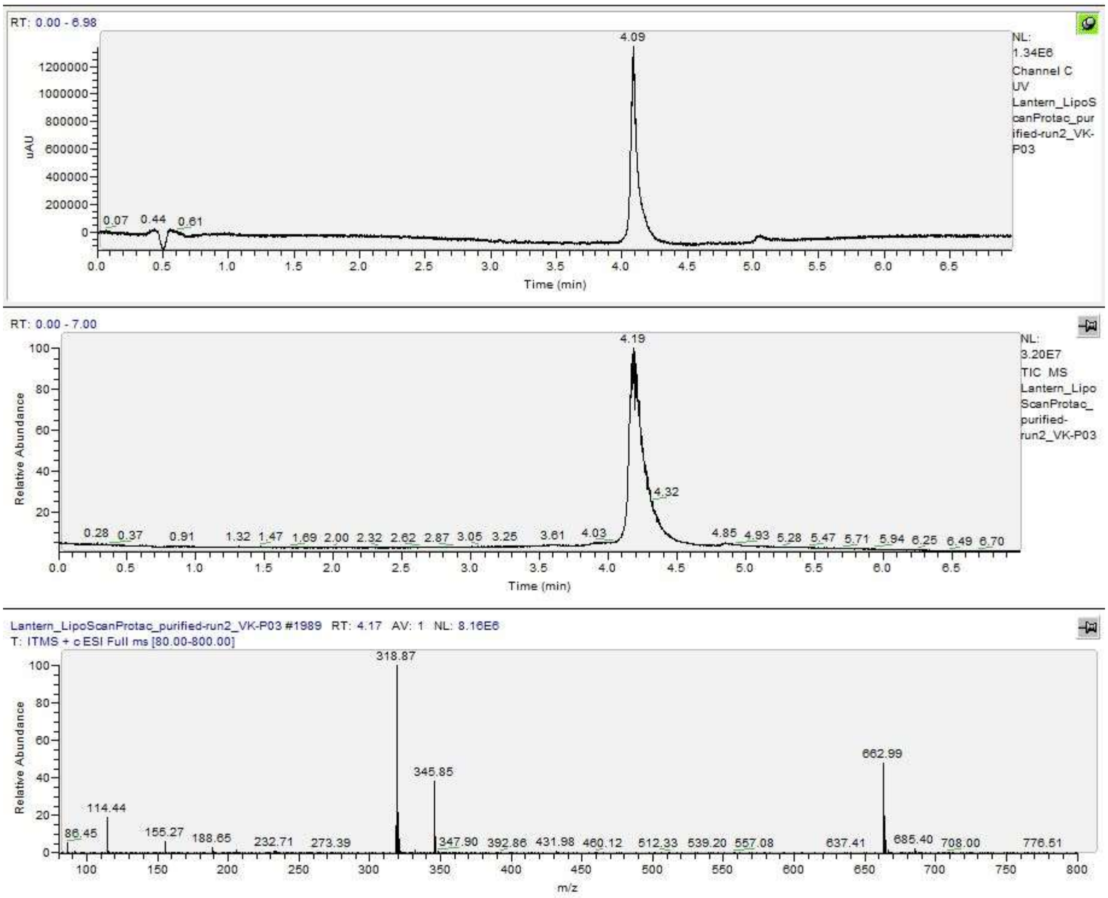

IV. VK-P04 (**4**)

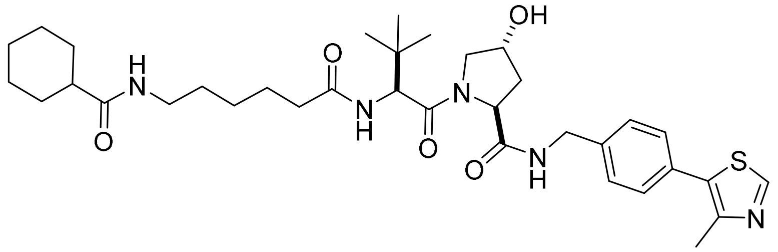

Purity by HPLC: >95%.

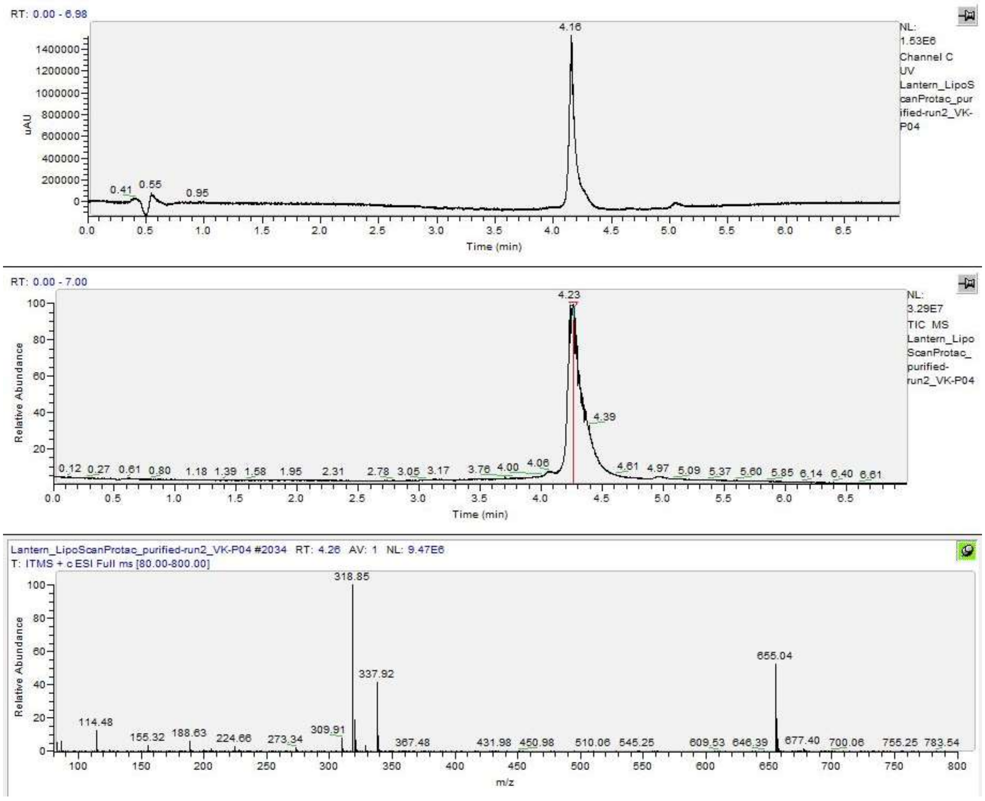

V. VK-P05 (**5**)

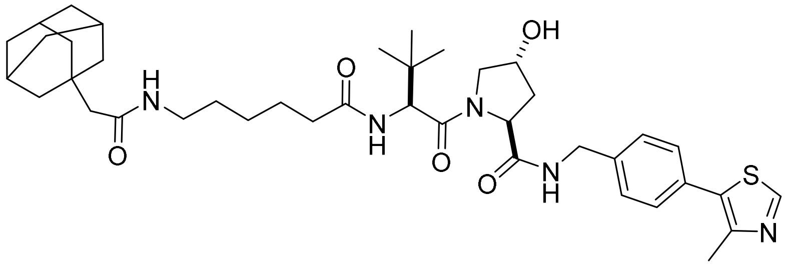

Purity by HPLC: >95%.

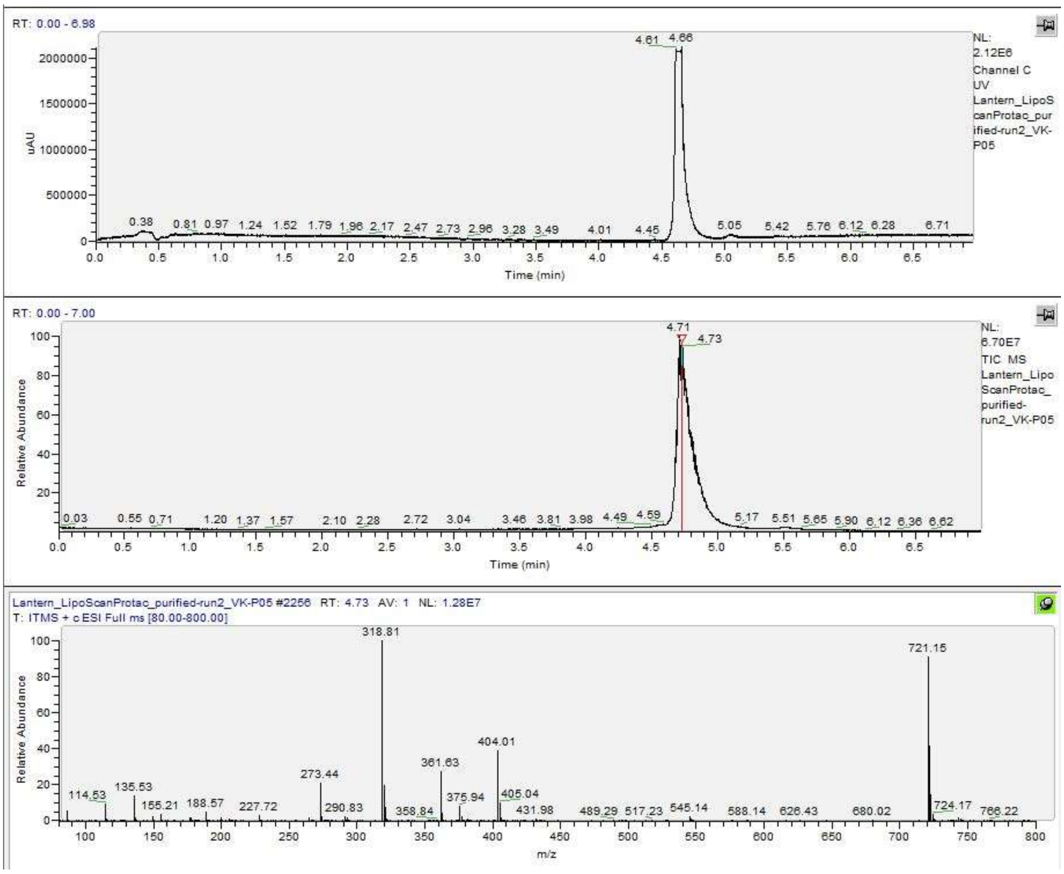

VI. VK-P06 (**6**)

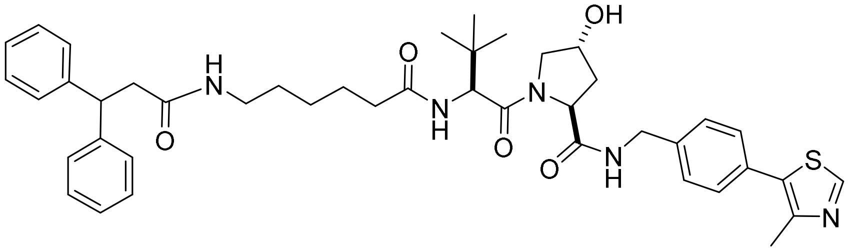

Purity by HPLC: >95%.

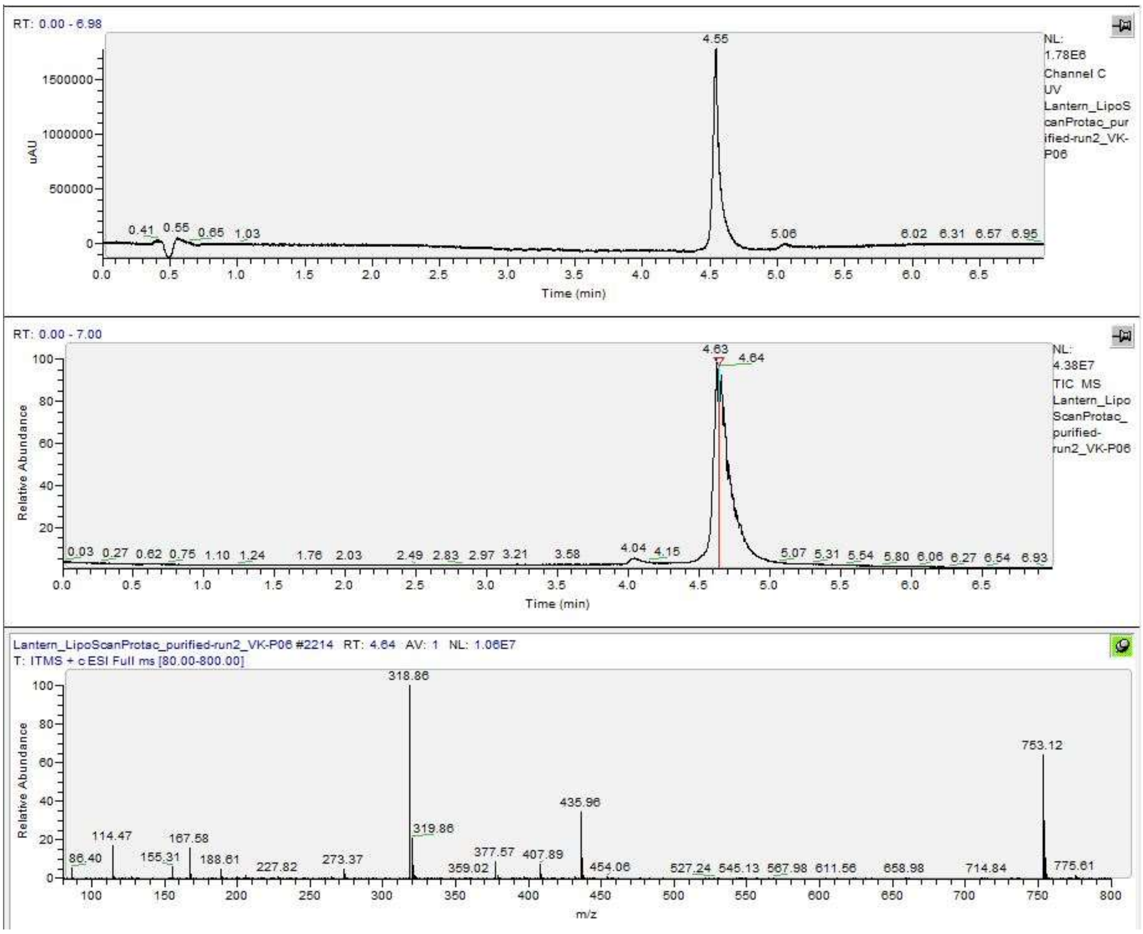

VII. VK-P07 (**7**)

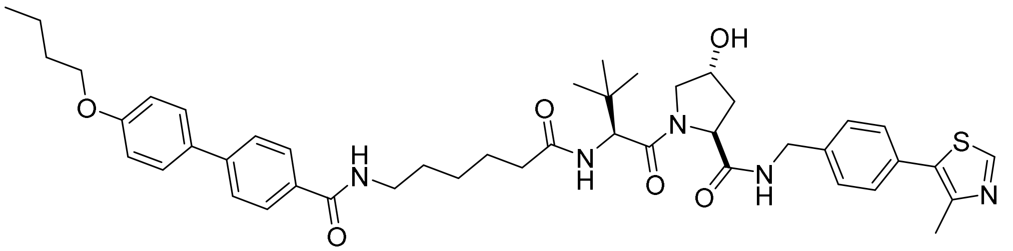

Purity by HPLC: >95%.

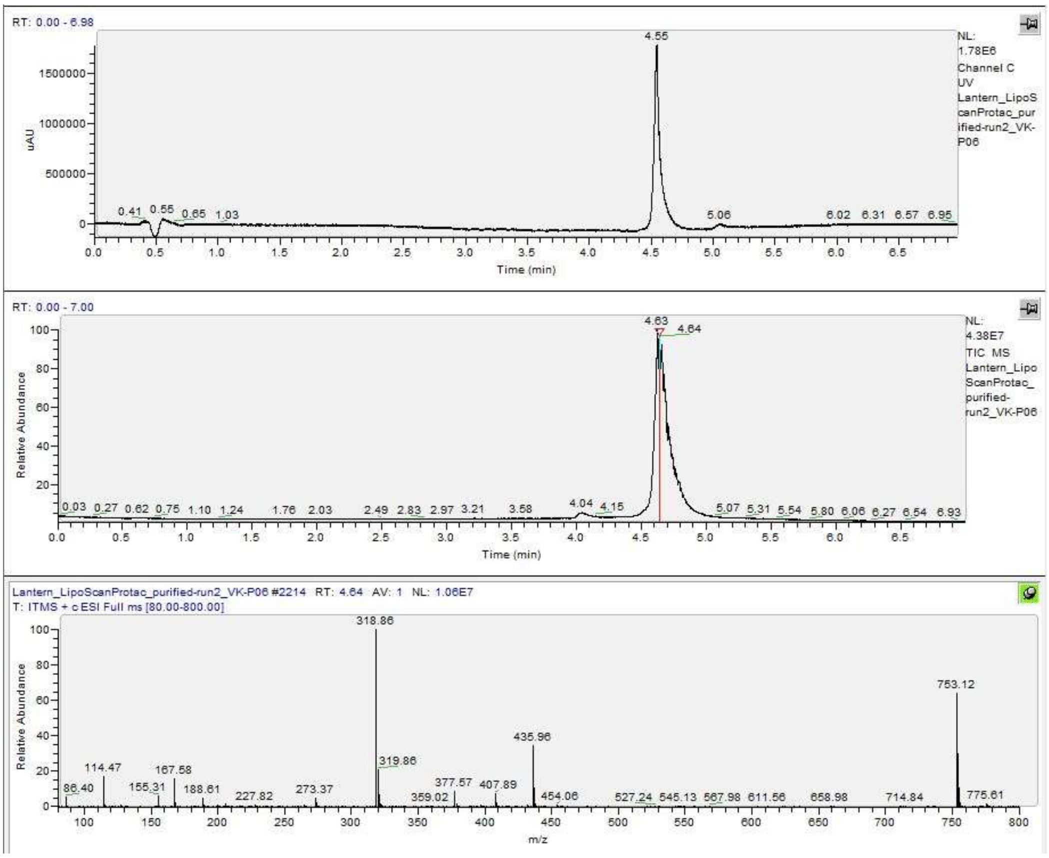

VIII. VK-P08 (**8**)

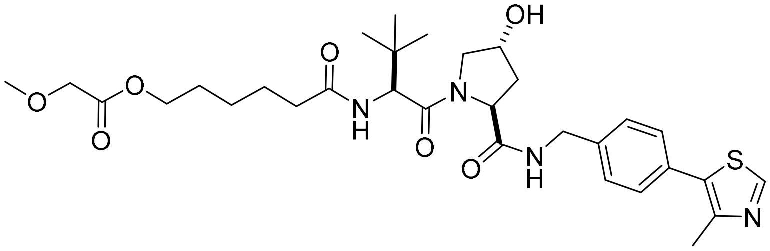

Purity by HPLC: 75%.

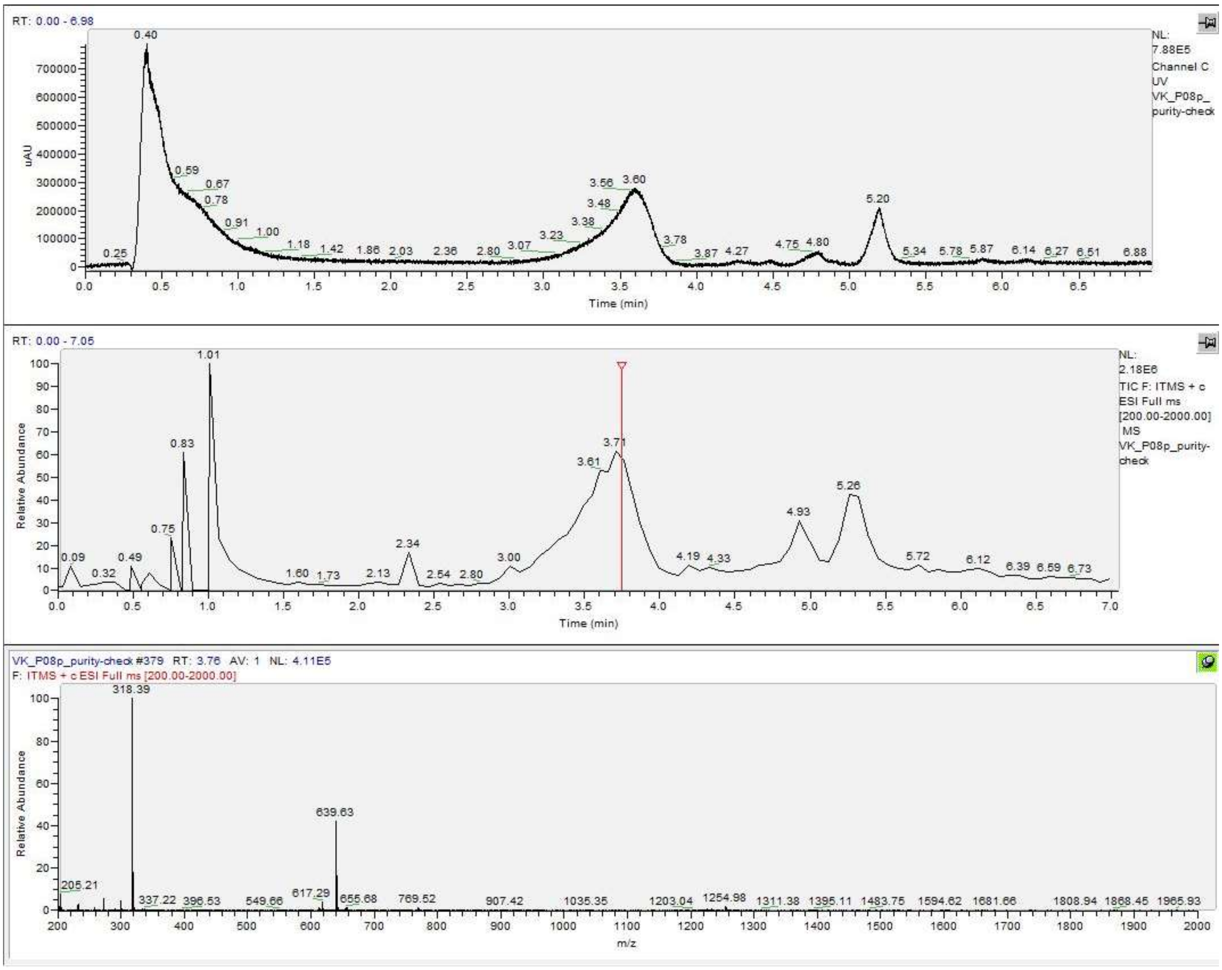

IX. VK-P09 (**9**)

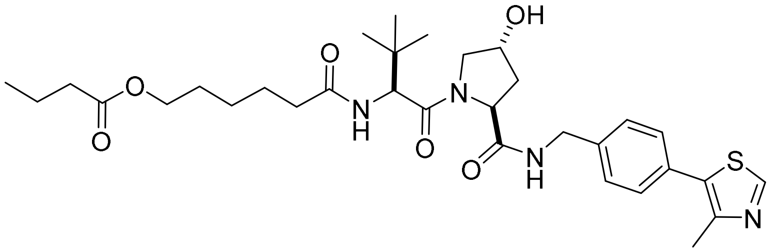

Purity by HPLC: >95%.

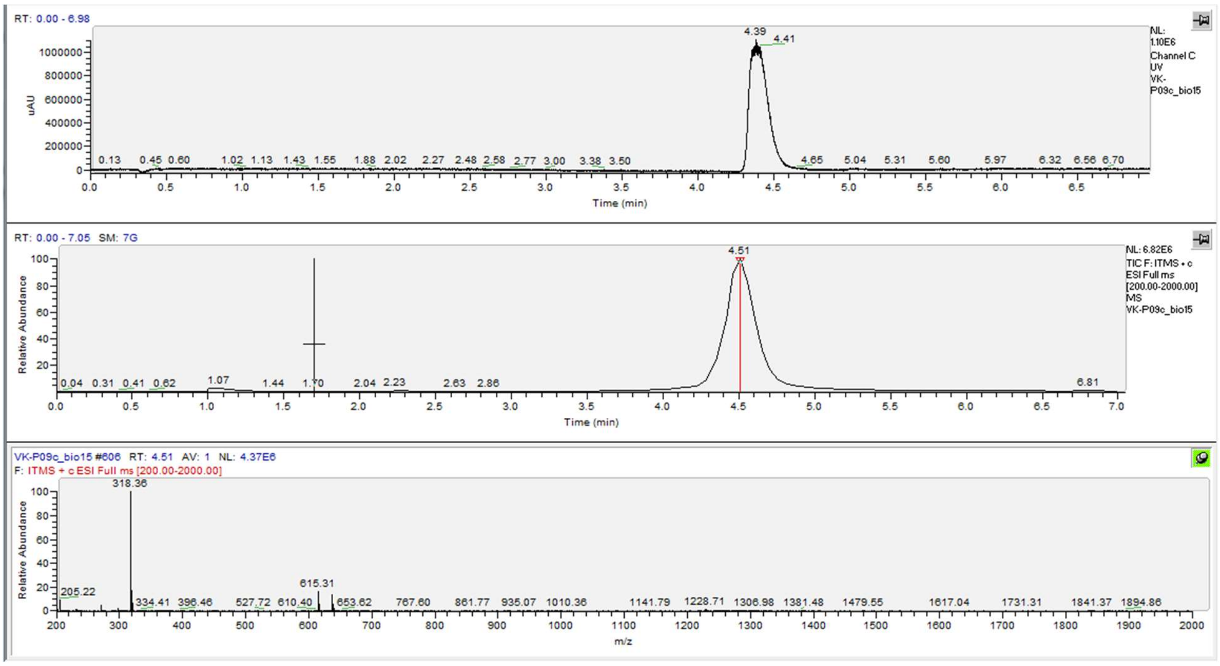

X. VK-P10 (**10**)

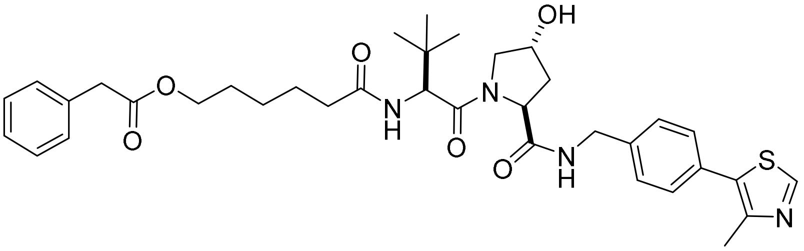

Purity by HPLC: >95%.

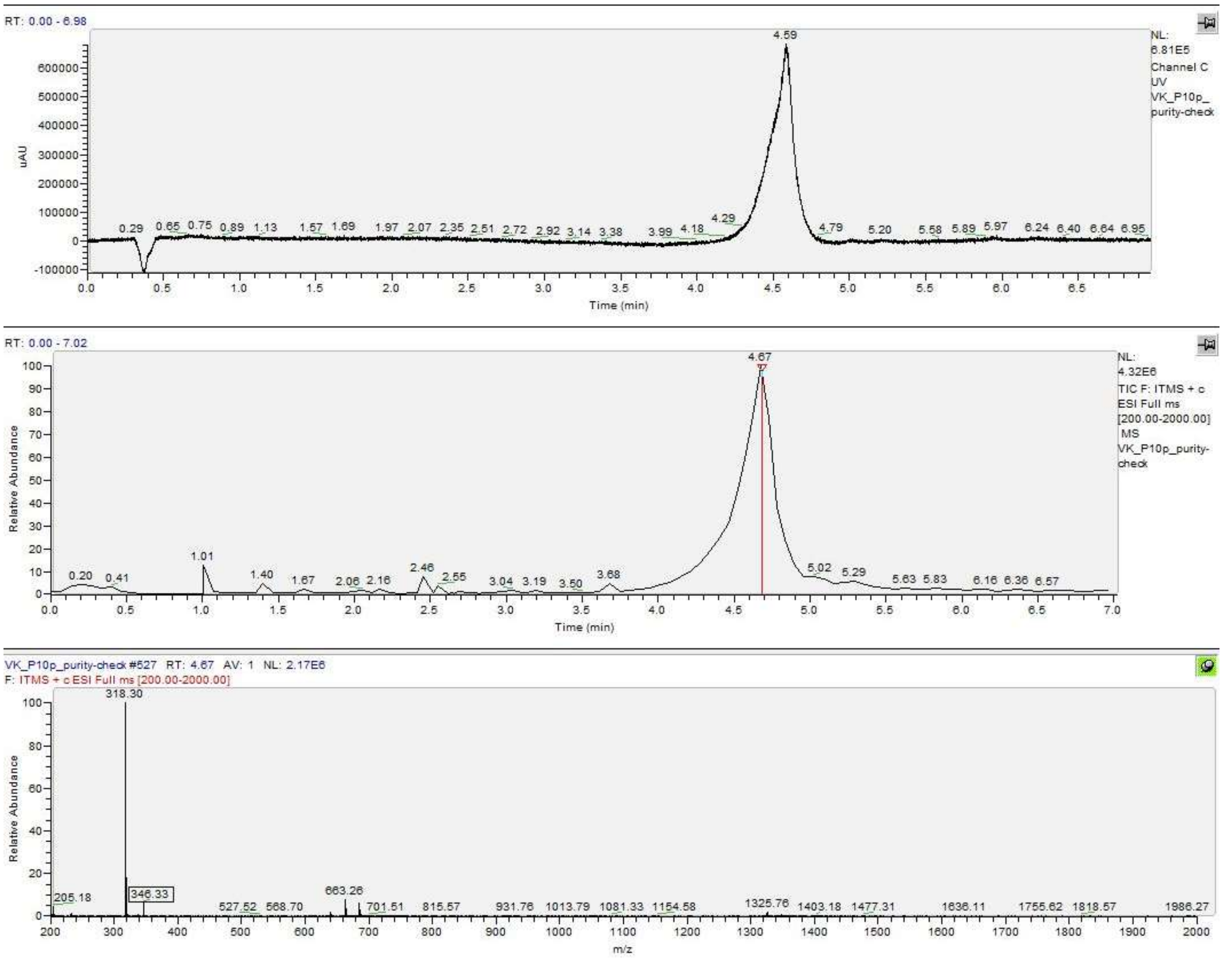

XI. VK-P11 (**11**)

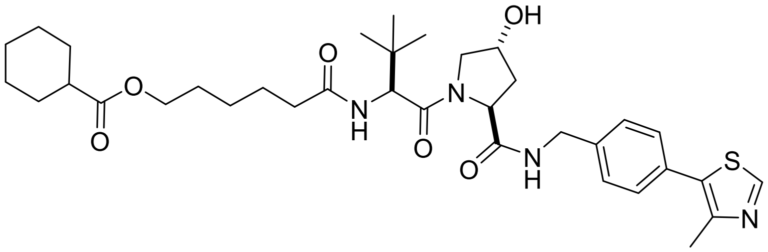

Purity by HPLC: >95%.

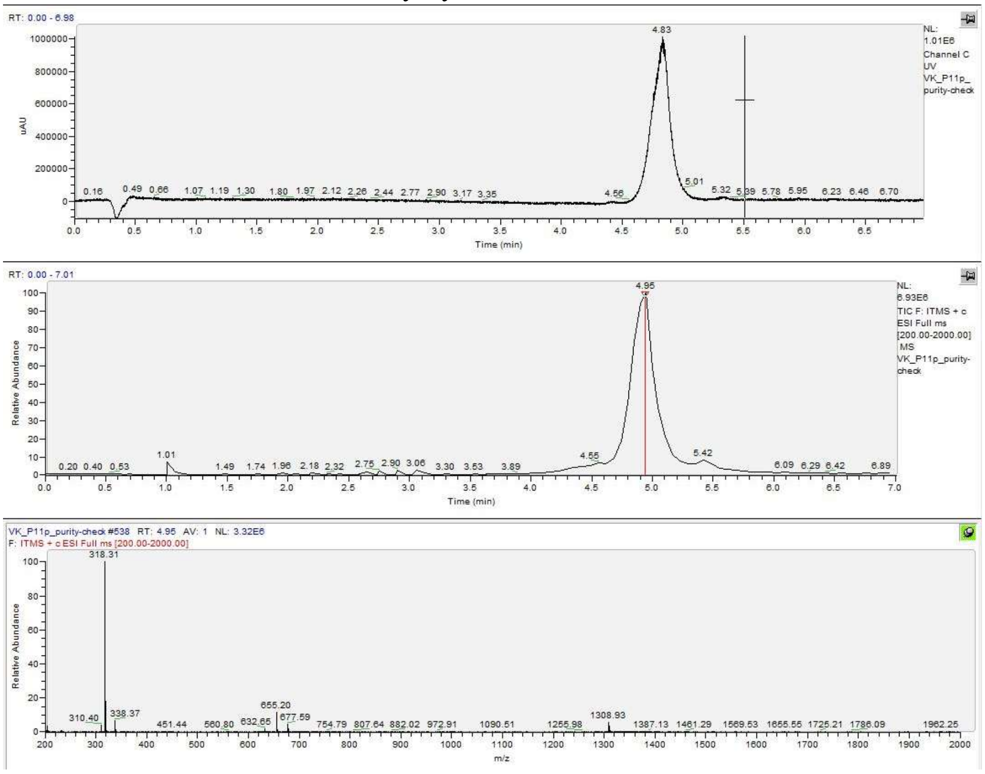

XII. VK-P12 (**12**)

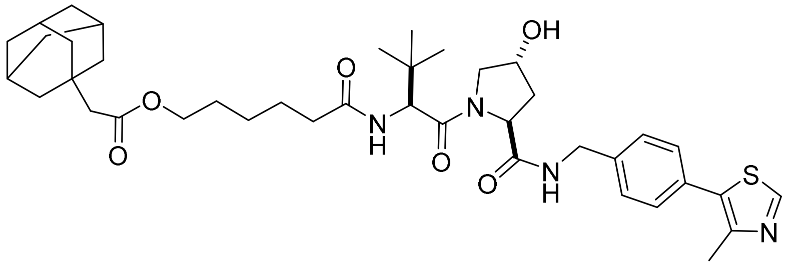

Purity by HPLC: >95%.

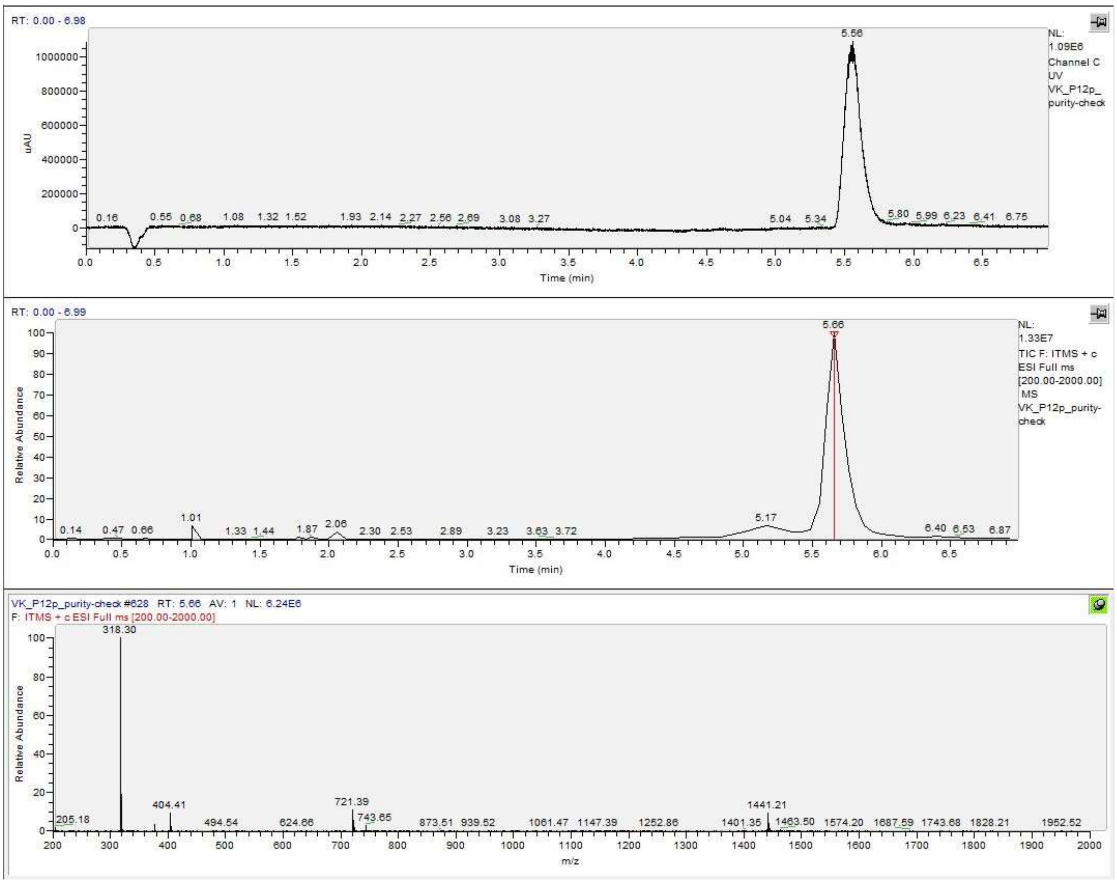

XIII. VK-P13 (**13**)

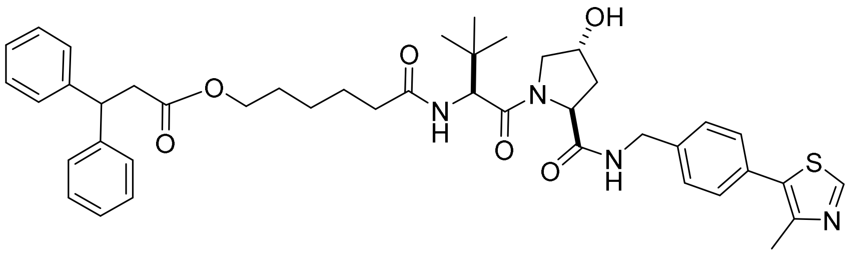

Purity by HPLC: >95%.

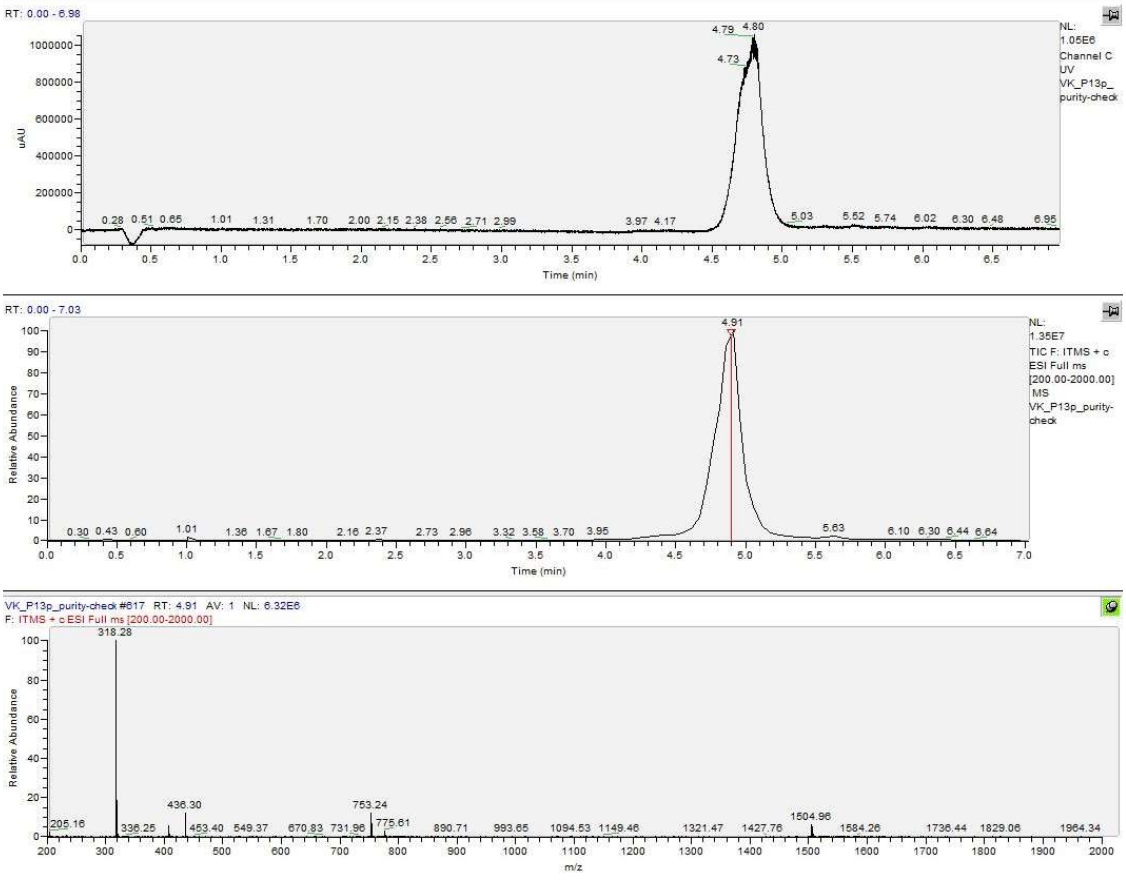

XIV. VK-P14 (**14**)

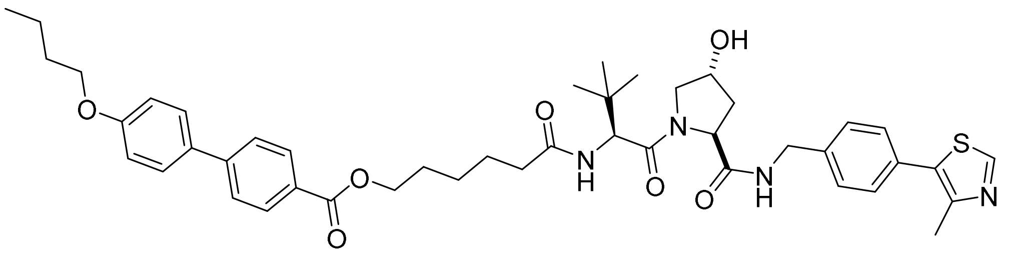

Purity by HPLC: 43%.

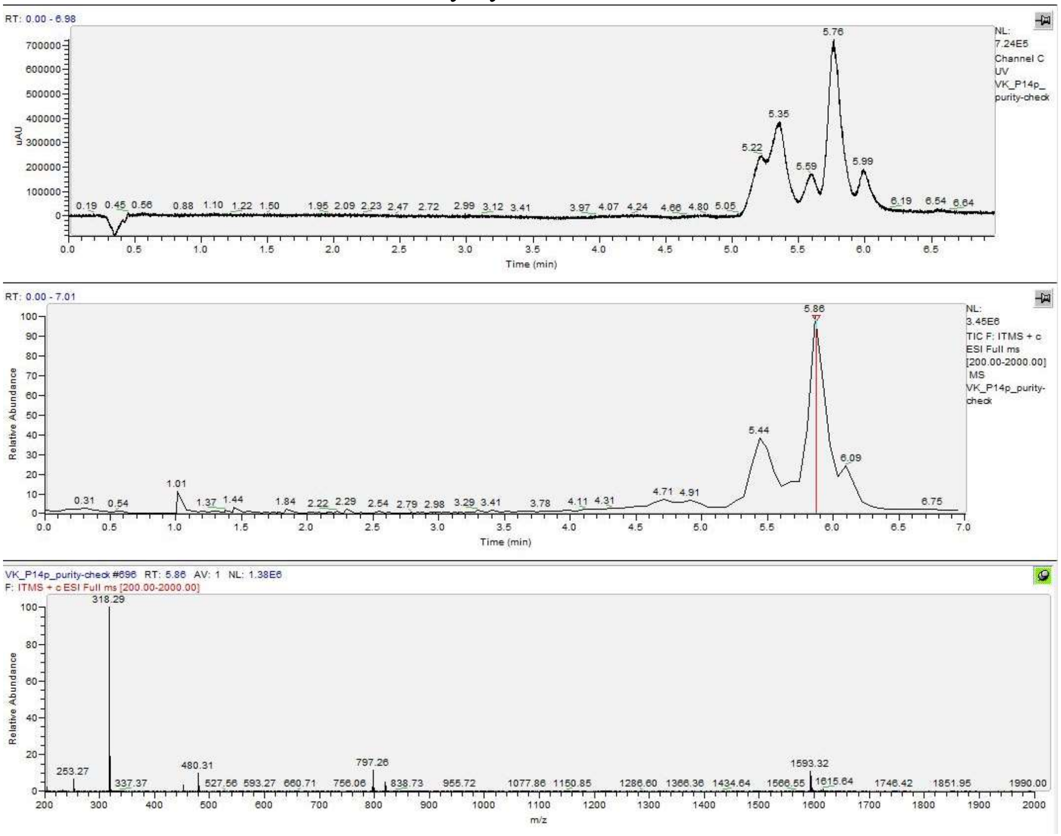

XV. VK-P15 (**15**)

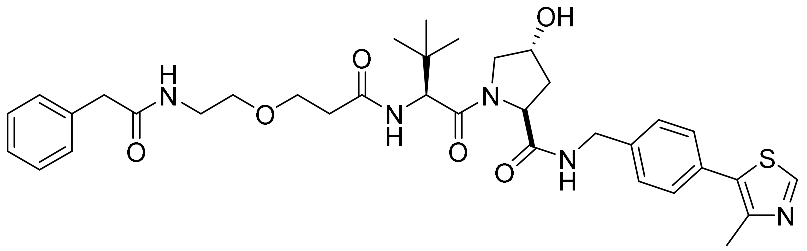

Purity by HPLC ~ 74%.

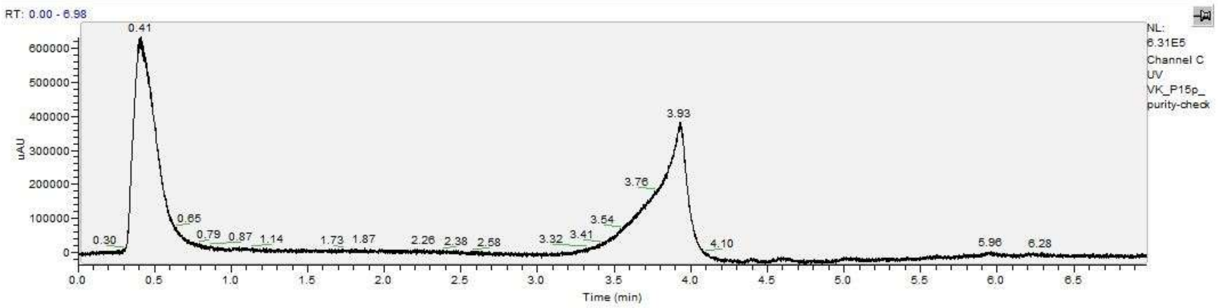

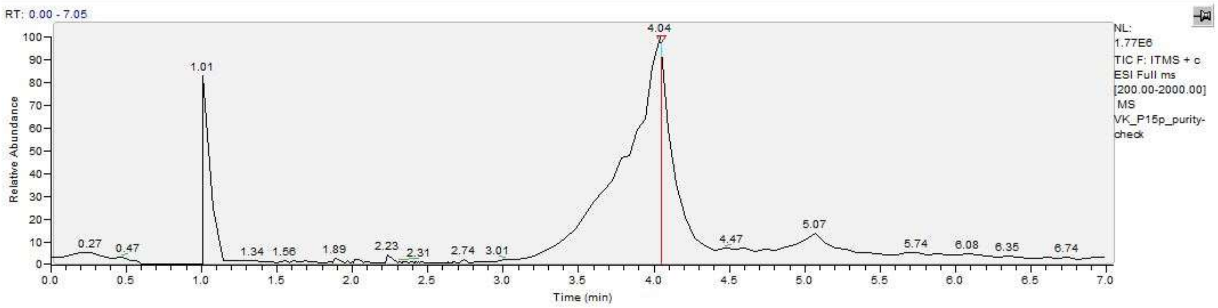

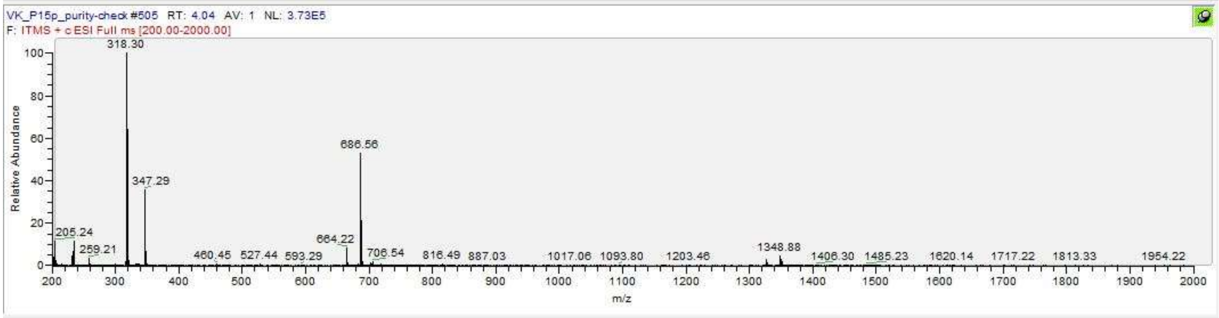

XVI. VK-P16 (**16**)

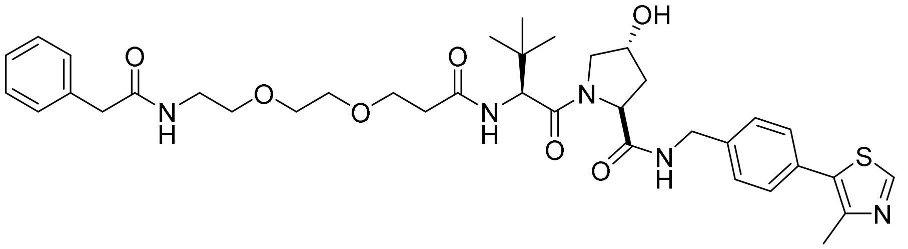

Purity by HPLC: >95%.

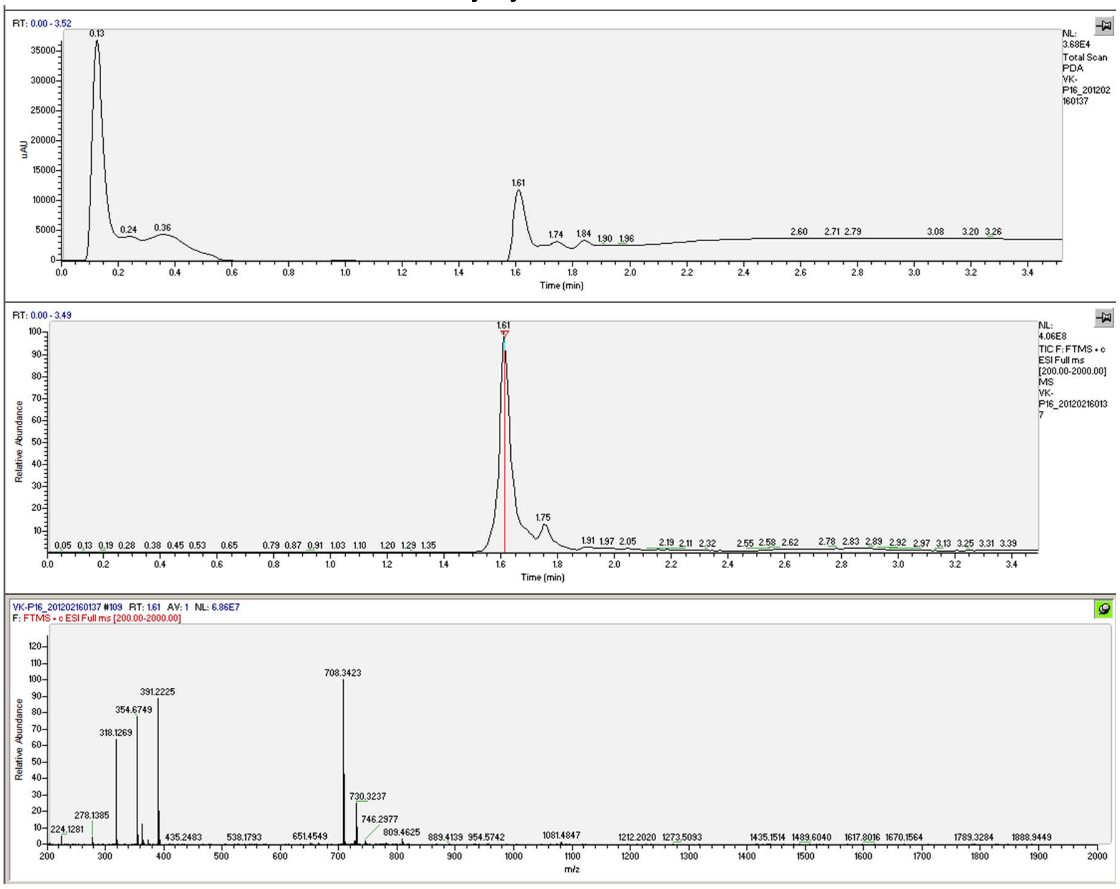

XVII. VK-P17 (**17**)

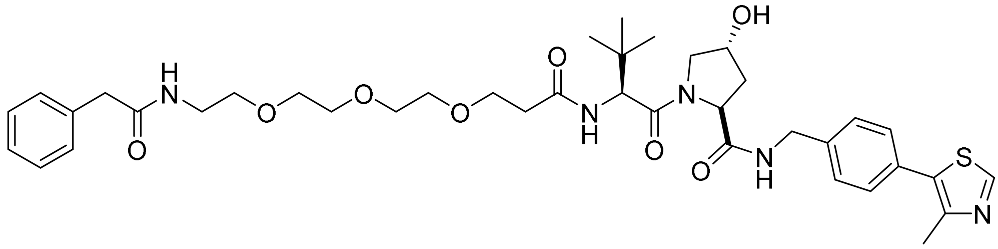

Purity by HPLC: >95%.

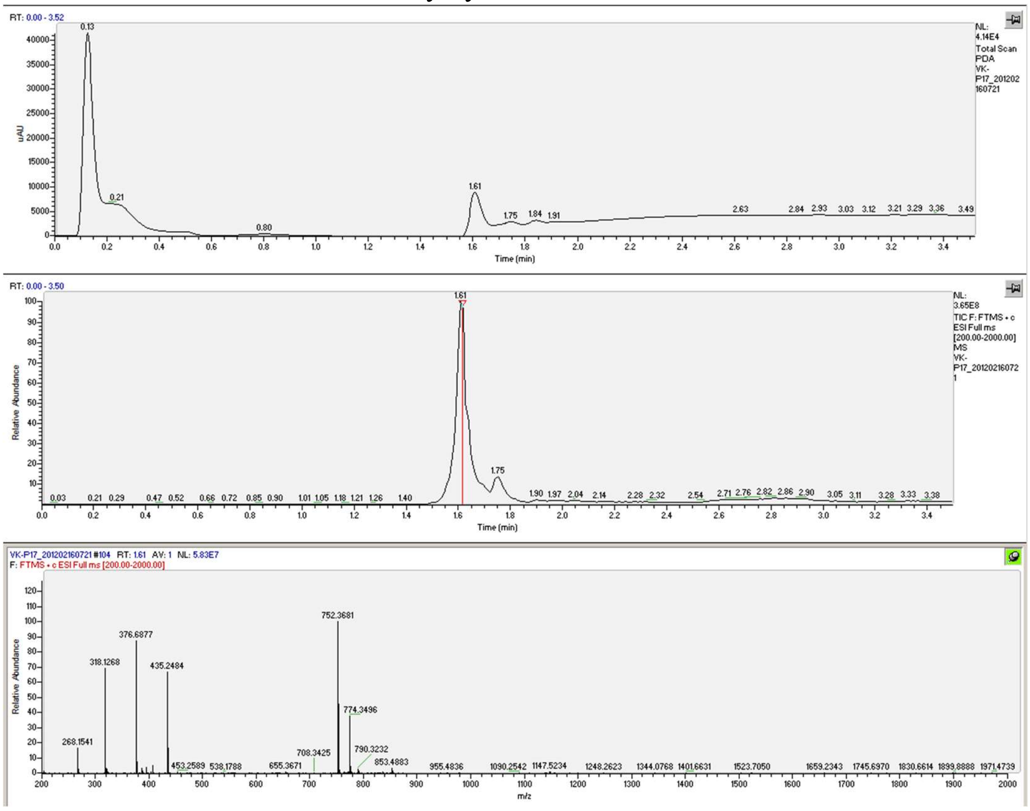

XVIII. VK-P18 (**18**)

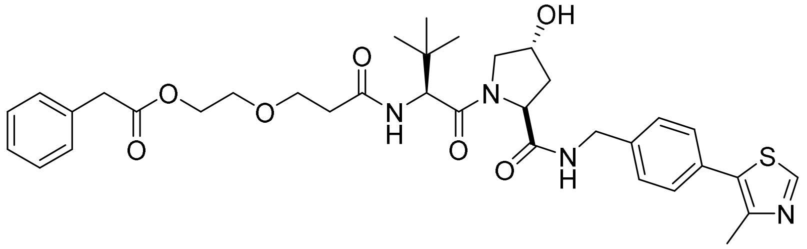

Purity by HPLC: >95%.

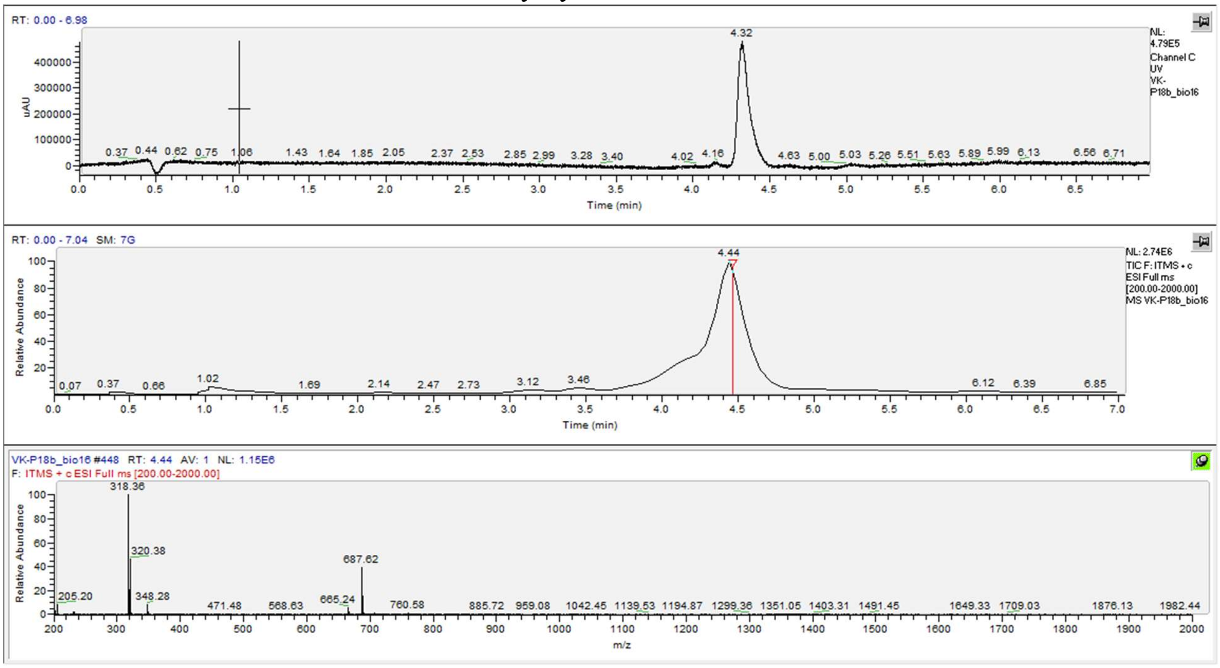

XIX. VK-P19 (**19**)

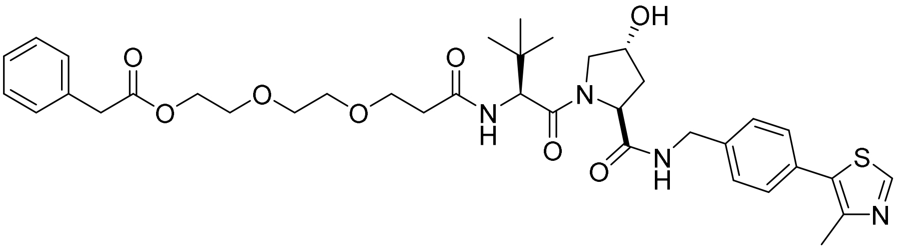

Purity by HPLC ~ 57%.

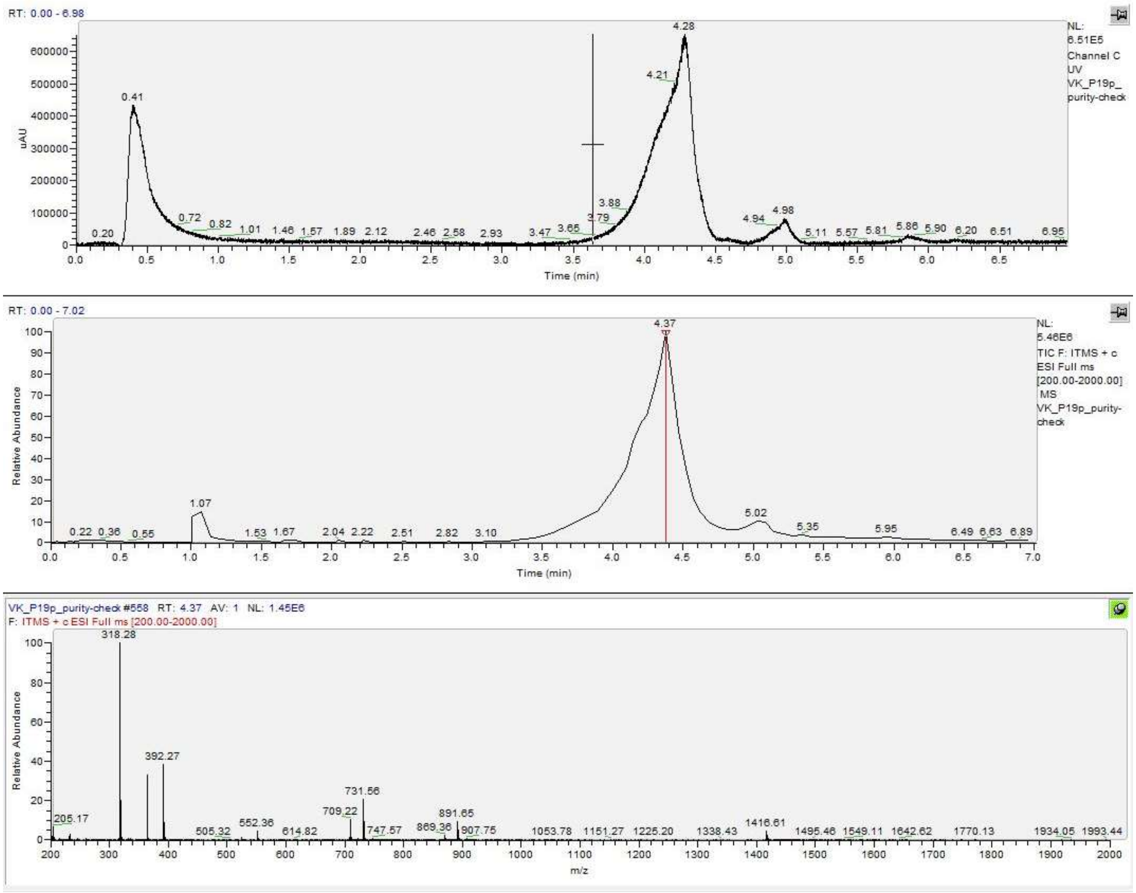

XX. VK-P20 (**20**)

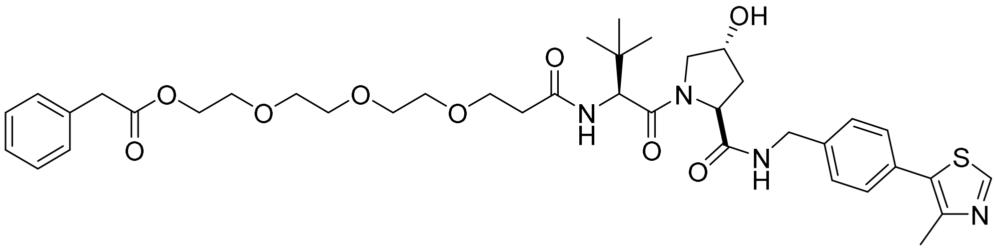

Purity by HPLC ~ 61%.

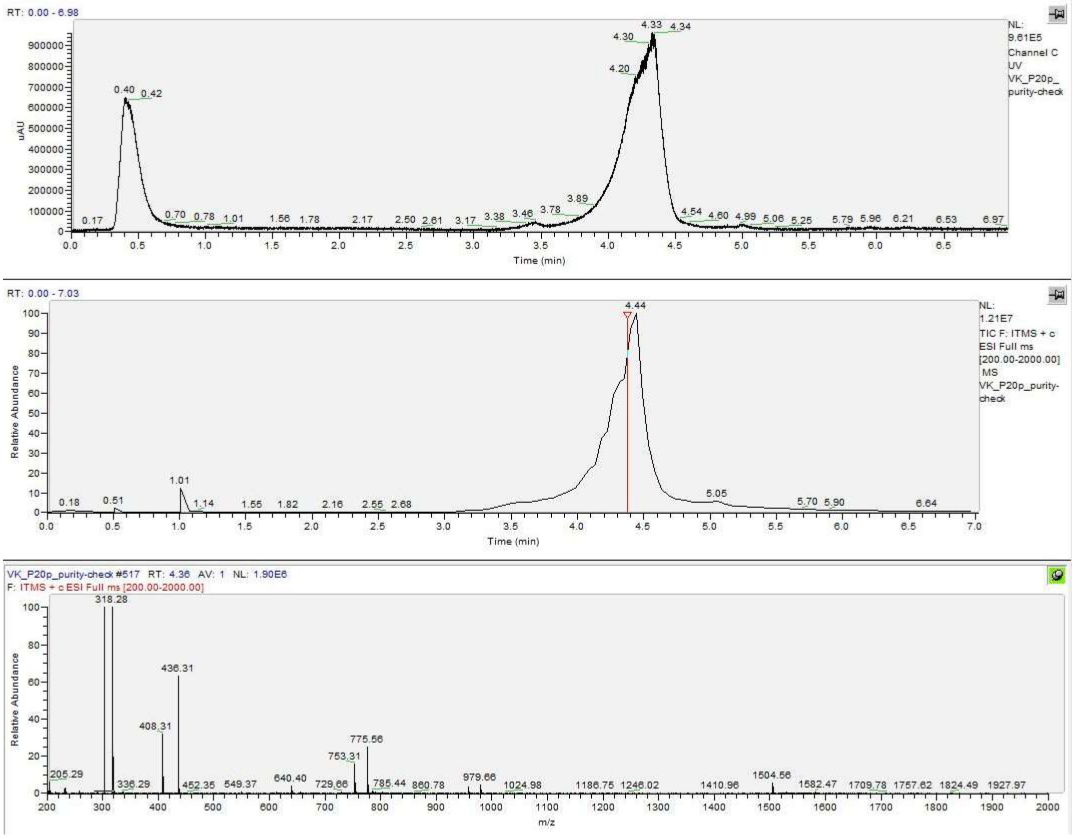

XXI. <u>AB1 (**23**)</u>

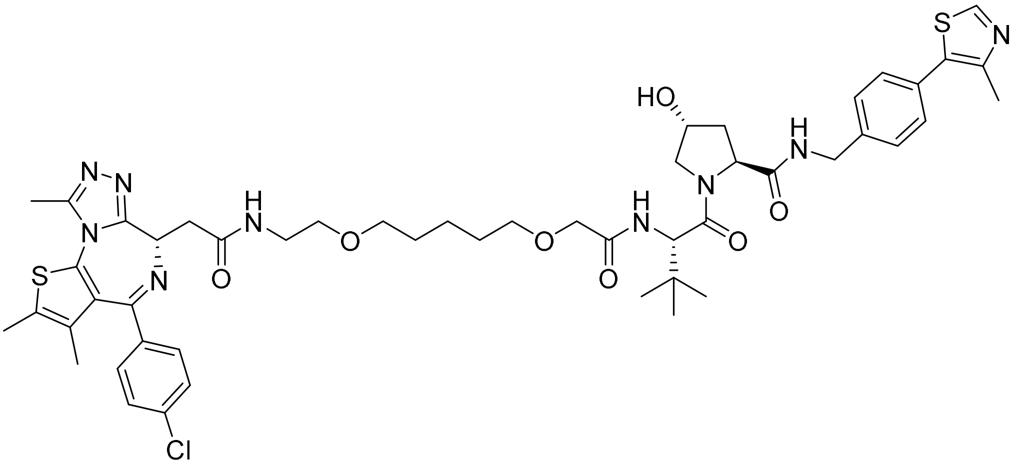

Purity by HPLC: >95%

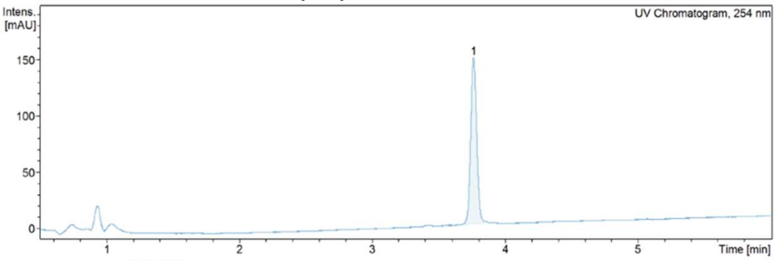

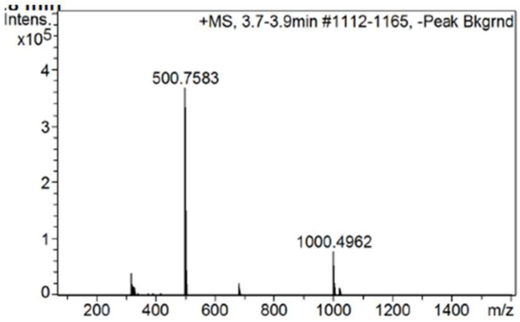

XXII. AB2 (**24**)

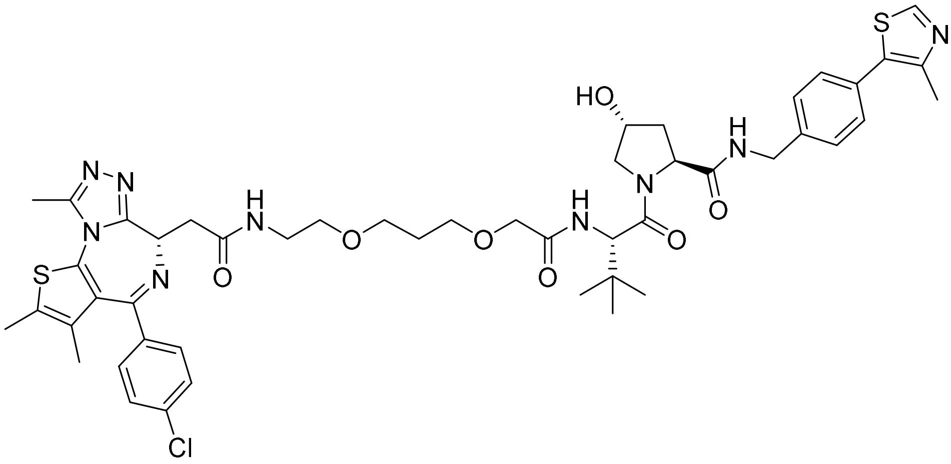

Purity by HPLC: >95%

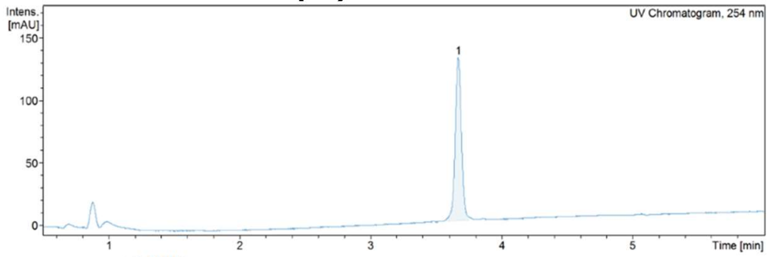

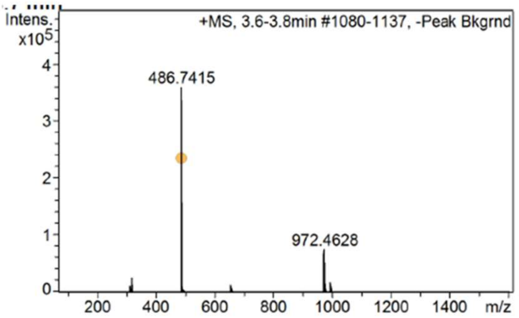

XXIII. OMZ1 (**25**)

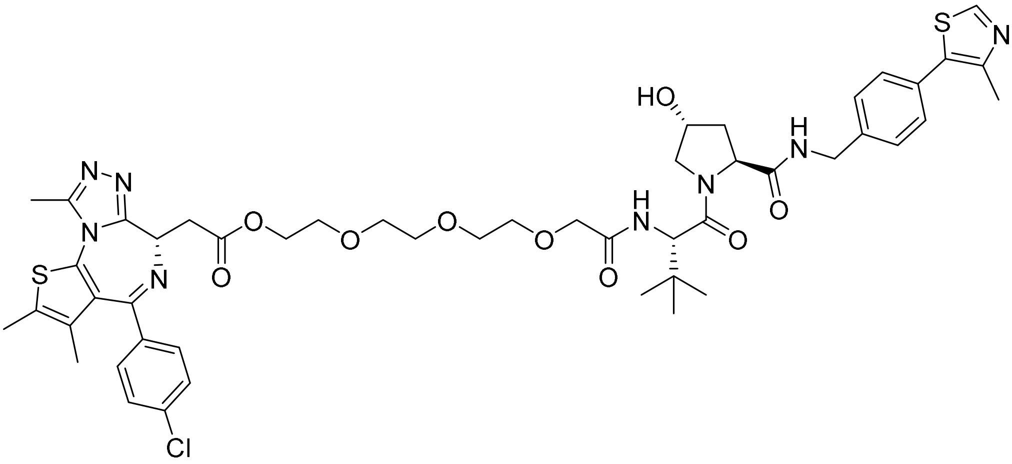

Purity by HPLC: >95%

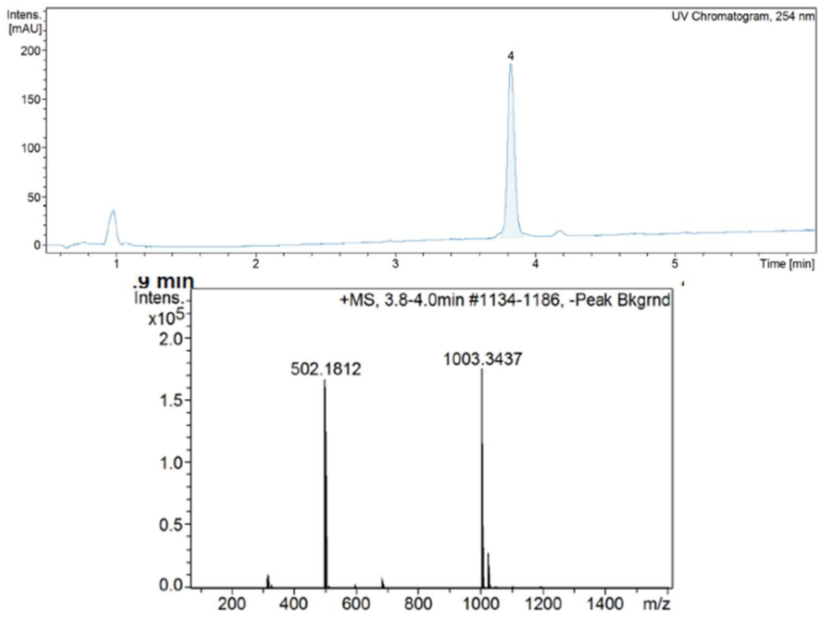

XXIV. OARV-771 (**26**)

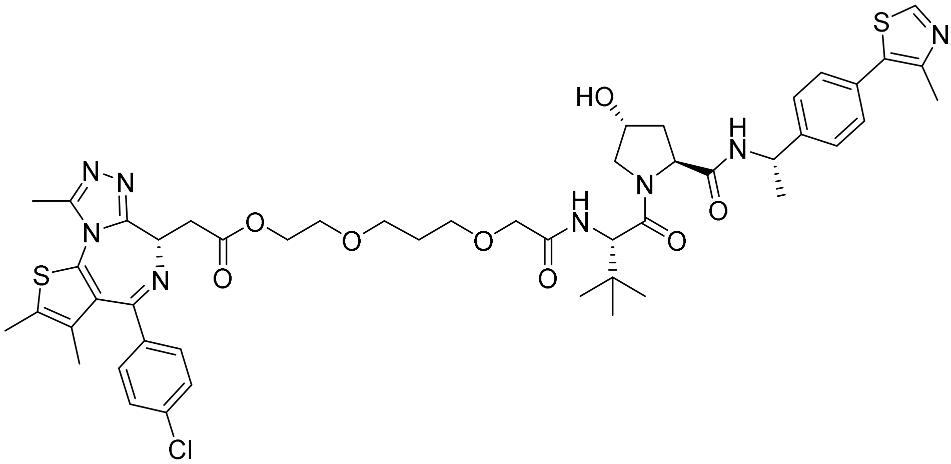

Purity by HPLC: >95%.

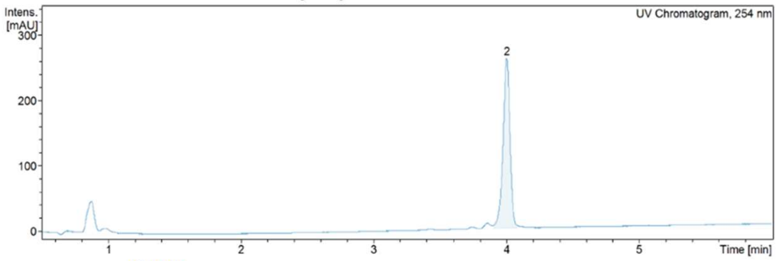

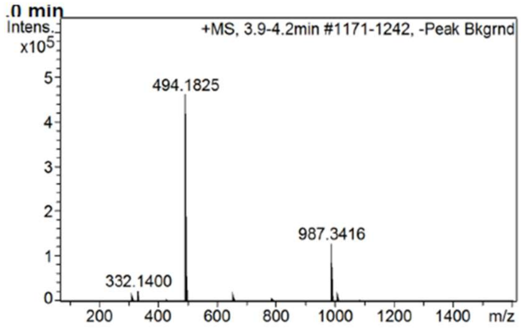

XXV. OAB1 (**27**)

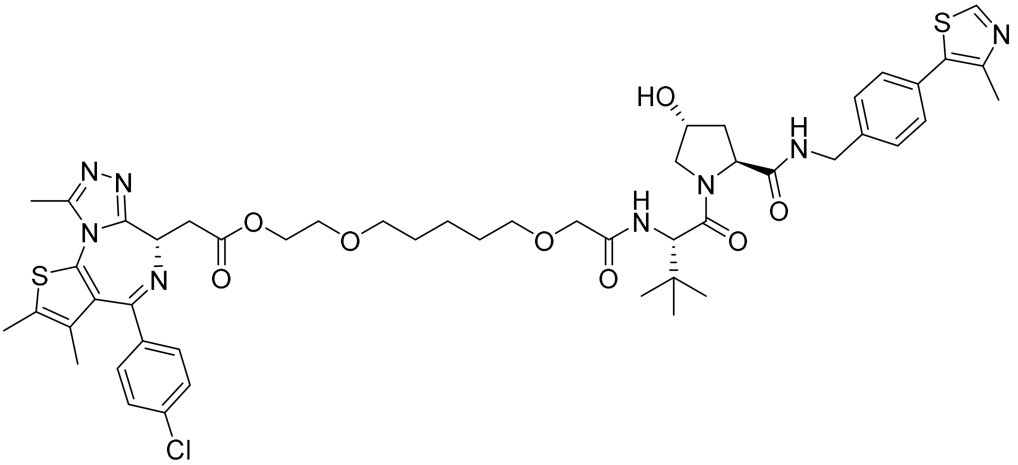

Purity by HPLC: >95%

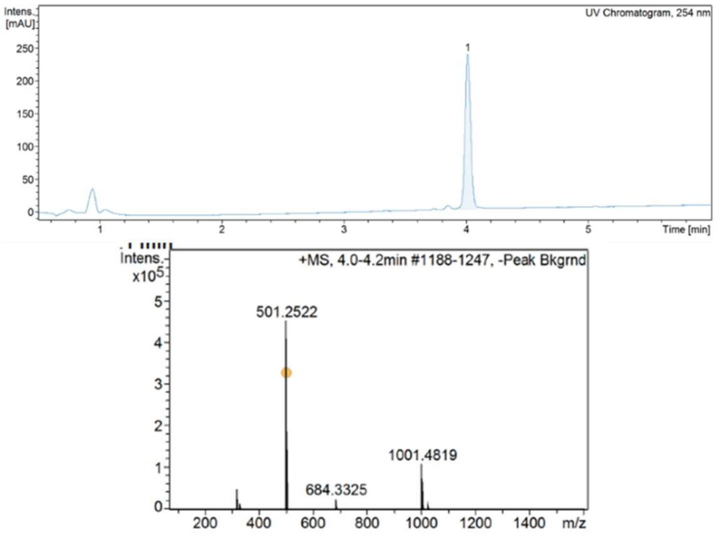

XXVI. OAB2 (**28**)

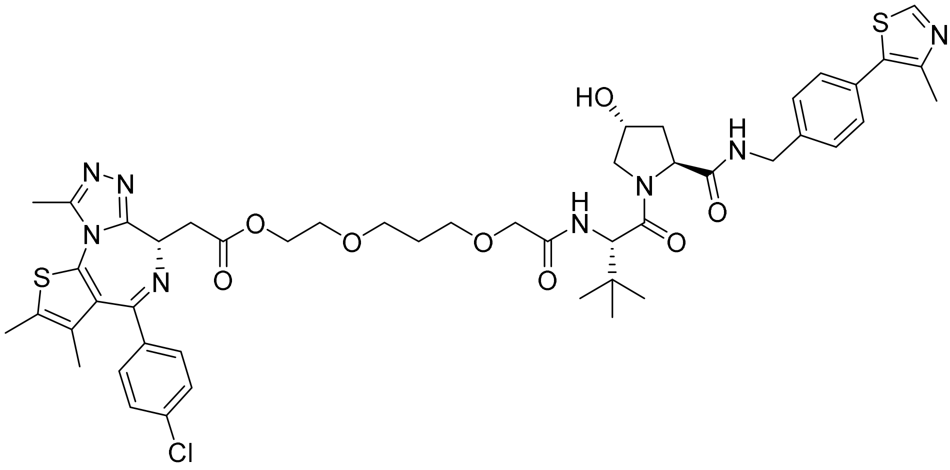

Purity by HPLC: >95%.

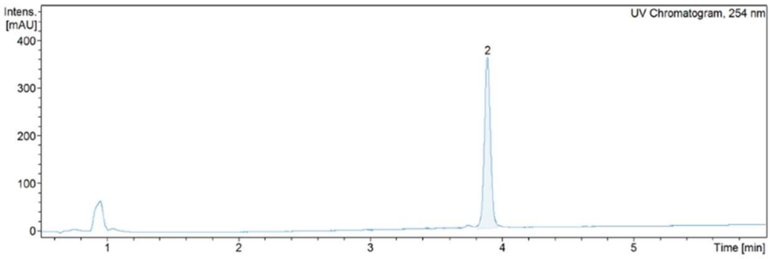

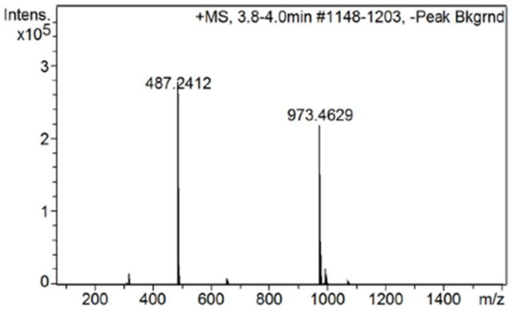

Liposcan Selected Ion Chromatograms for Compounds 1 – 20

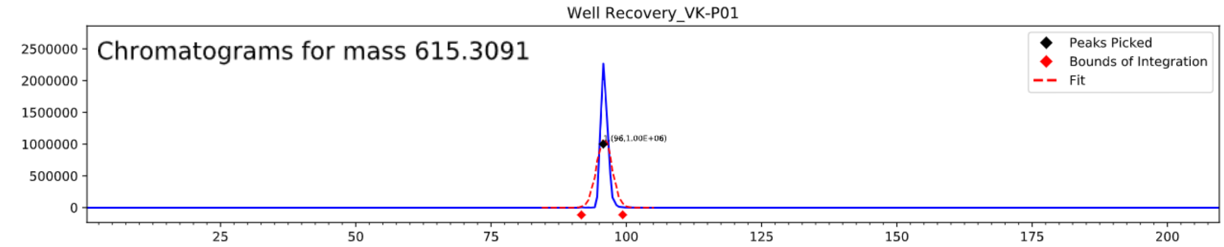

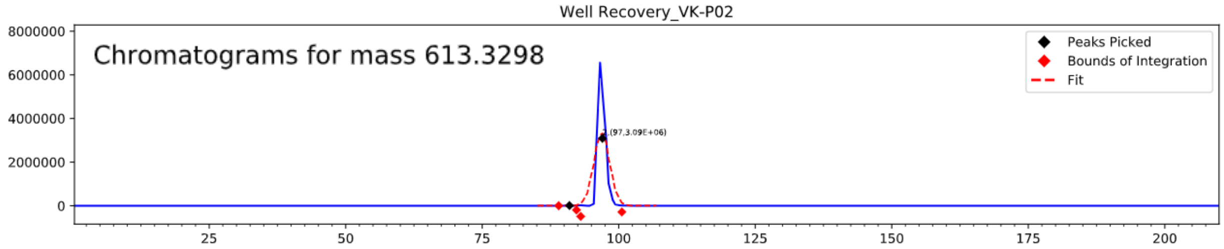

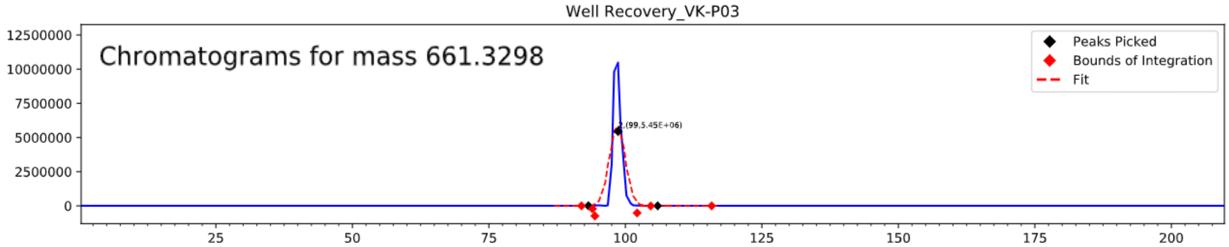

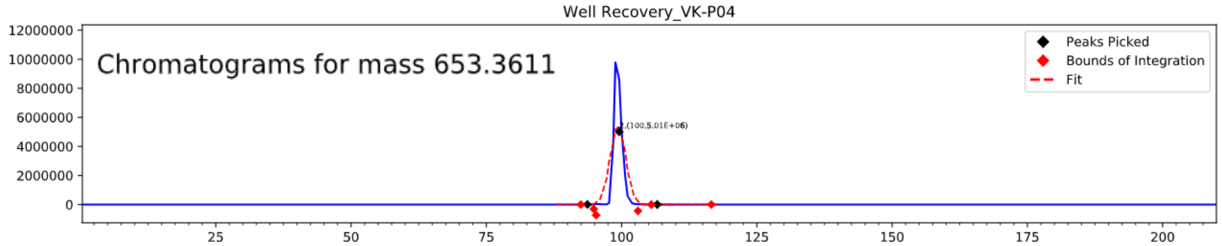

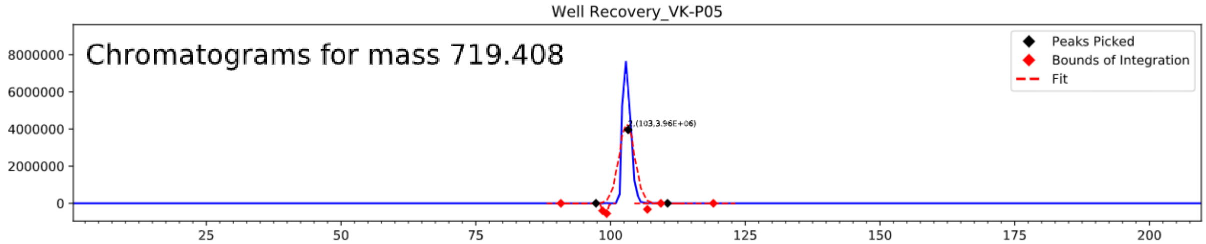

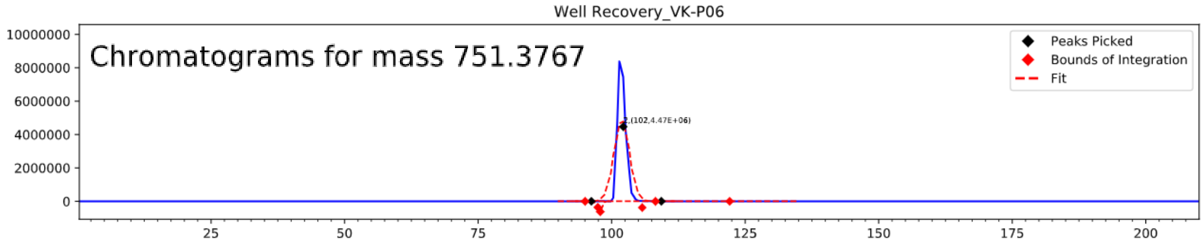

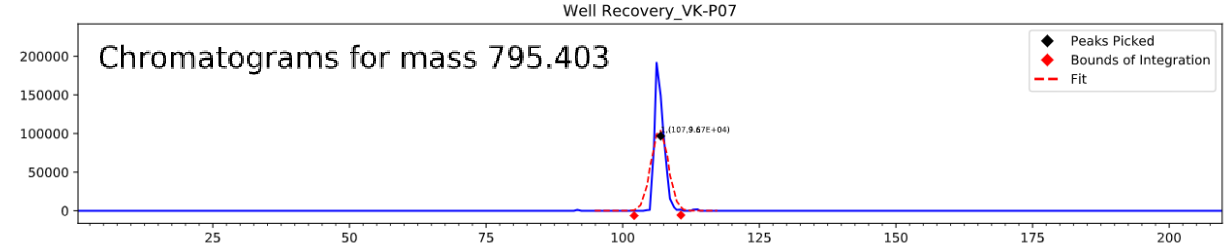
Well Recovery_VK-P07
Chromatograms for mass 795.403
Peaks Picked
Bounds of Integration
Fit
(107,9.67E+04)

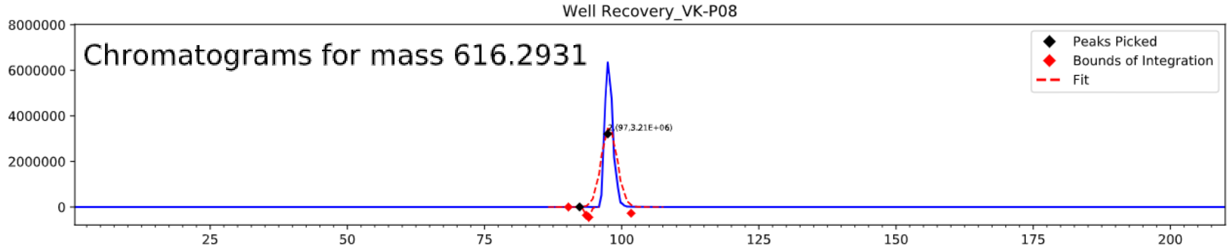
Well Recovery_VK-P08
Chromatograms for mass 616.2931
Peaks Picked
Bounds of Integration
Fit
(97,3.21E+06)

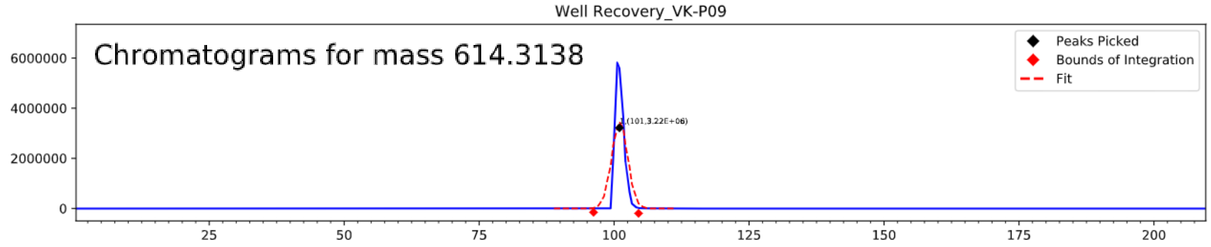
Well Recovery_VK-P09
Chromatograms for mass 614.3138
Peaks Picked
Bounds of Integration
Fit
(101,3.22E+06)

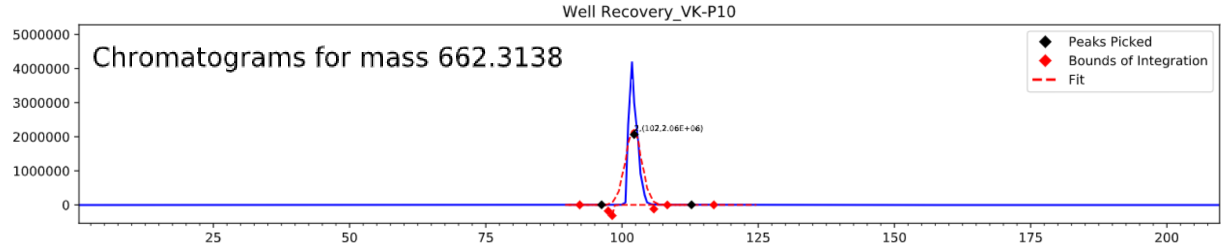
Well Recovery_VK-P10
Chromatograms for mass 662.3138
Peaks Picked
Bounds of Integration
Fit
(102,2.06E+06)

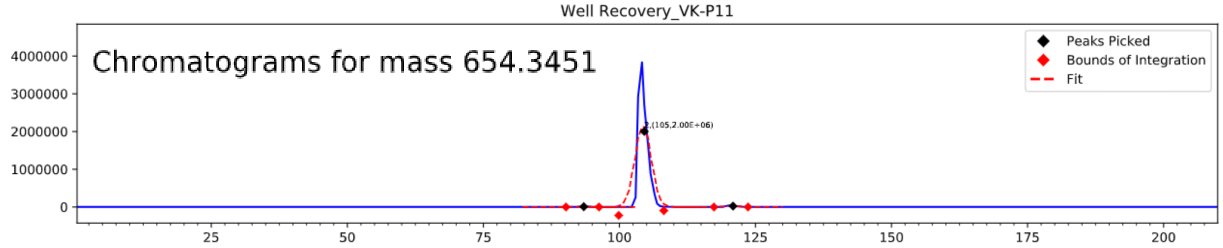
Well Recovery_VK-P11
Chromatograms for mass 654.3451
Peaks Picked
Bounds of Integration
Fit
(105,2.00E+06)

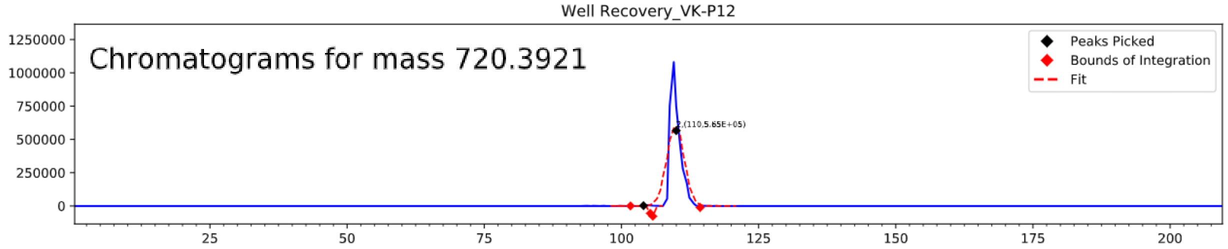
Well Recovery_VK-P12
Chromatograms for mass 720.3921
Peaks Picked
Bounds of Integration
Fit
(110,5.65E+05)

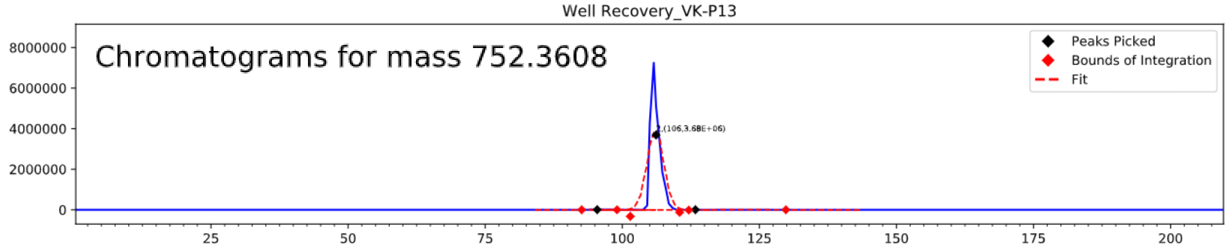
Well Recovery_VK-P13
Chromatograms for mass 752.3608
Peaks Picked
Bounds of Integration
Fit
(106,3.68E+06)

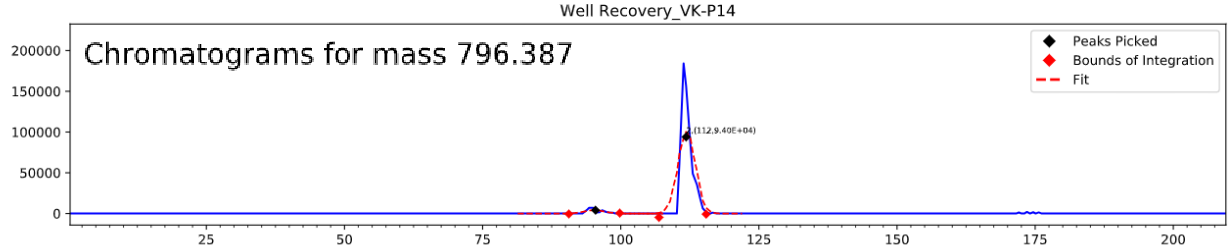
Well Recovery_VK-P14
Chromatograms for mass 796.387
Peaks Picked
Bounds of Integration
Fit
(112,9.40E+04)

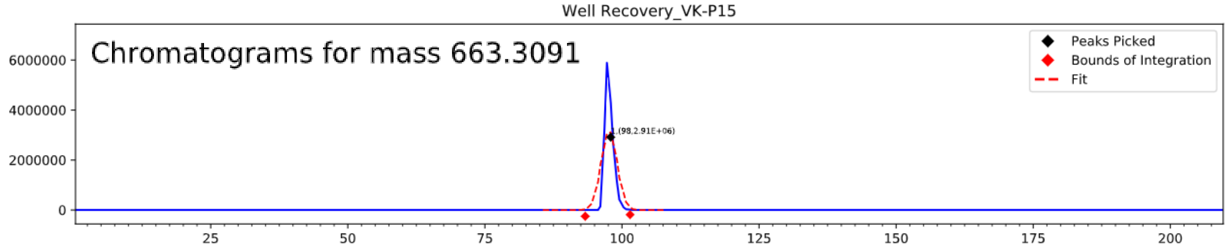
Well Recovery_VK-P15
Chromatograms for mass 663.3091
Peaks Picked
Bounds of Integration
Fit
(98,2.91E+06)

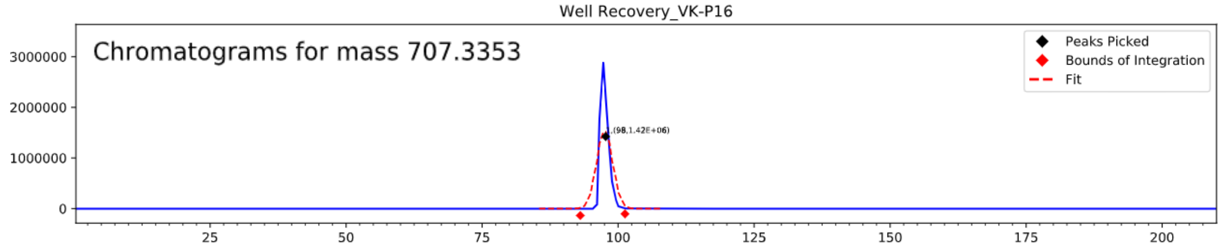
Well Recovery_VK-P16
Chromatograms for mass 707.3353
Peaks Picked
Bounds of Integration
Fit
(98,1.42E+06)

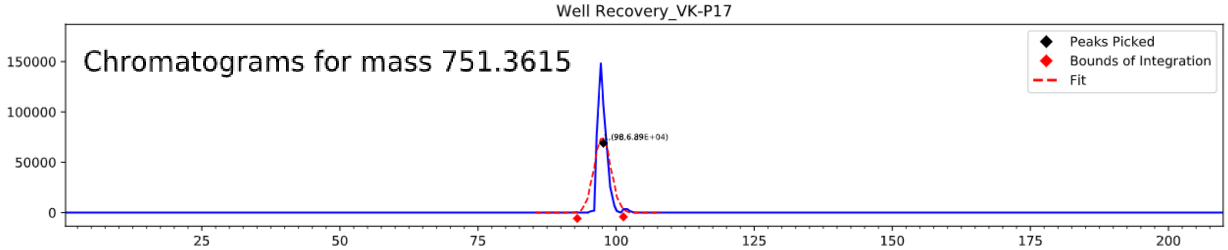
Well Recovery_VK-P17
Chromatograms for mass 751.3615
Peaks Picked
Bounds of Integration
Fit
(98,6.89E+04)

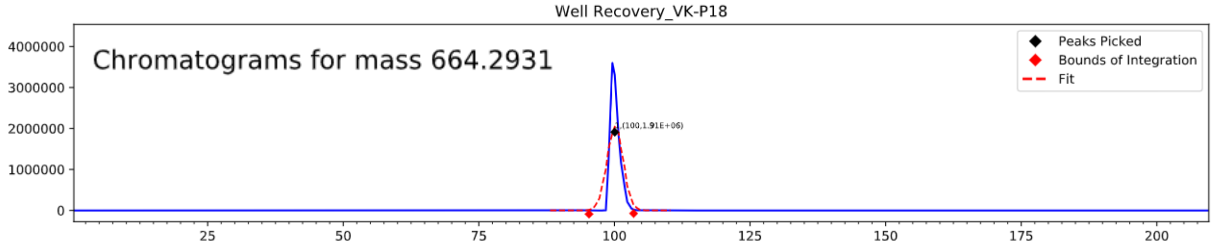
Well Recovery_VK-P18
Chromatograms for mass 664.2931
Peaks Picked
Bounds of Integration
Fit
(100,1.91E+06)

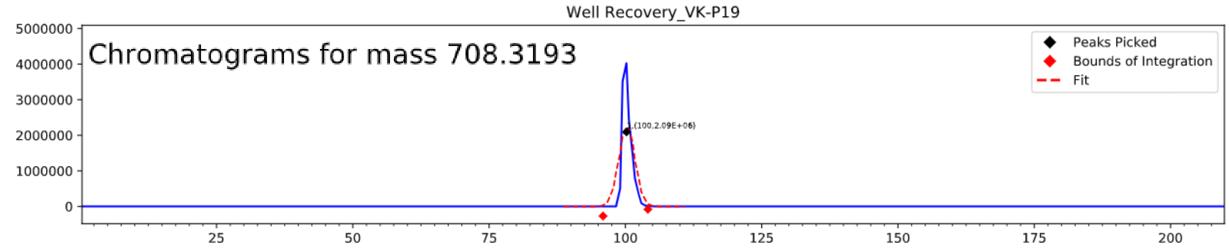
Well Recovery_VK-P19
Chromatograms for mass 708.3193
Peaks Picked
Bounds of Integration
Fit
(100,2.09E+06)

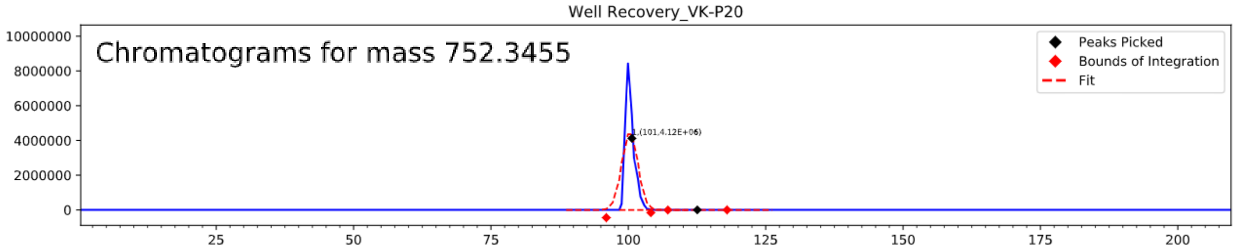
Well Recovery_VK-P20
Chromatograms for mass 752.3455
Peaks Picked
Bounds of Integration
Fit
(101,4.12E+06)
